# Restoring expression of tumour suppressor PTEN by engineered circular RNA‐enhanced Osimertinib sensitivity in non‐small cell lung cancer

**DOI:** 10.1002/ctm2.1792

**Published:** 2024-08-21

**Authors:** Haoran Li, Zheng Liu, Shaoyi Chen, Jingsheng Cai, Peiyu Wang, Kezhong Chen, Mantang Qiu

**Affiliations:** ^1^ Thoracic Oncology Institute/Research Unit of Intelligence Diagnosis and Treatment in Early Non‐Small Cell Lung Cancer Peking University People's Hospital Beijing China; ^2^ Department of Thoracic Surgery Peking University People's Hospital Beijing China; ^3^ Institute of Advanced Clinical Medicine Peking University Beijing China

Dear Editor,

This study provides a new strategy to construct circular RNA (circRNA) in vitro named NeoAna, with splicing sites concealed in CVB3_IRES. Re‐storing phosphatase and tensin homologue deleted on chromosome 10 (PTEN) expression by engineered circRNA enhances sensitivity to Osimertinib in non‐small lung cancer (NSCLC).

Previous Anabaena permuted intron‐exon system could permit the circularisation of sequences up to 5 kb in length, significantly longer than previously reported; however, it is important to acknowledge the presence of ‘scar sequences’ in the final products (Figure 1A).^1^, ^2^, ^3^ We designed the NeoAna systems to synthesise circRNAs (Figure 1B) without scar sequences (Figure S1). As shown in Figure 1C, the circRNA (enhanced green fluorescent protein [EGFP], as an example) is clearly observed and resistance to RNase R treatment. Indeed, the formation of circRNA was further confirmed by PCR and the exact splicing site was determined by Sanger sequencing (Figure 1D,E). Successful protein translation was confirmed in cells (Figure 1F,H). Then, in vitro transcription (IVT) products of NeoAna system were subjected to high‐performance liquid chromatography and each fraction was transfected into 293T cells, and the main peak showed strongest protein expression (Figure 1G,I‒K). Then, we synthesised pseudo‐uridine‐modified linear EGFP (m1ψ‐EGFP), cEGFP_Ana and cEGFP_NeoAna (Figure S2A) and transfected three RNAs into 293T and H1299 cells and green fluorescence and protein expression were observed in cells (Figure S2B,C). Compared with cEGFP_Ana, cEGFP_NeoAna induced weaker innate immunity response in 293T cells (Figure 1L). Besides, we found that the stability of cEGFP_NeoAna is comparable to that of cEGFP_Ana (Figure S3A).

**FIGURE 1:**
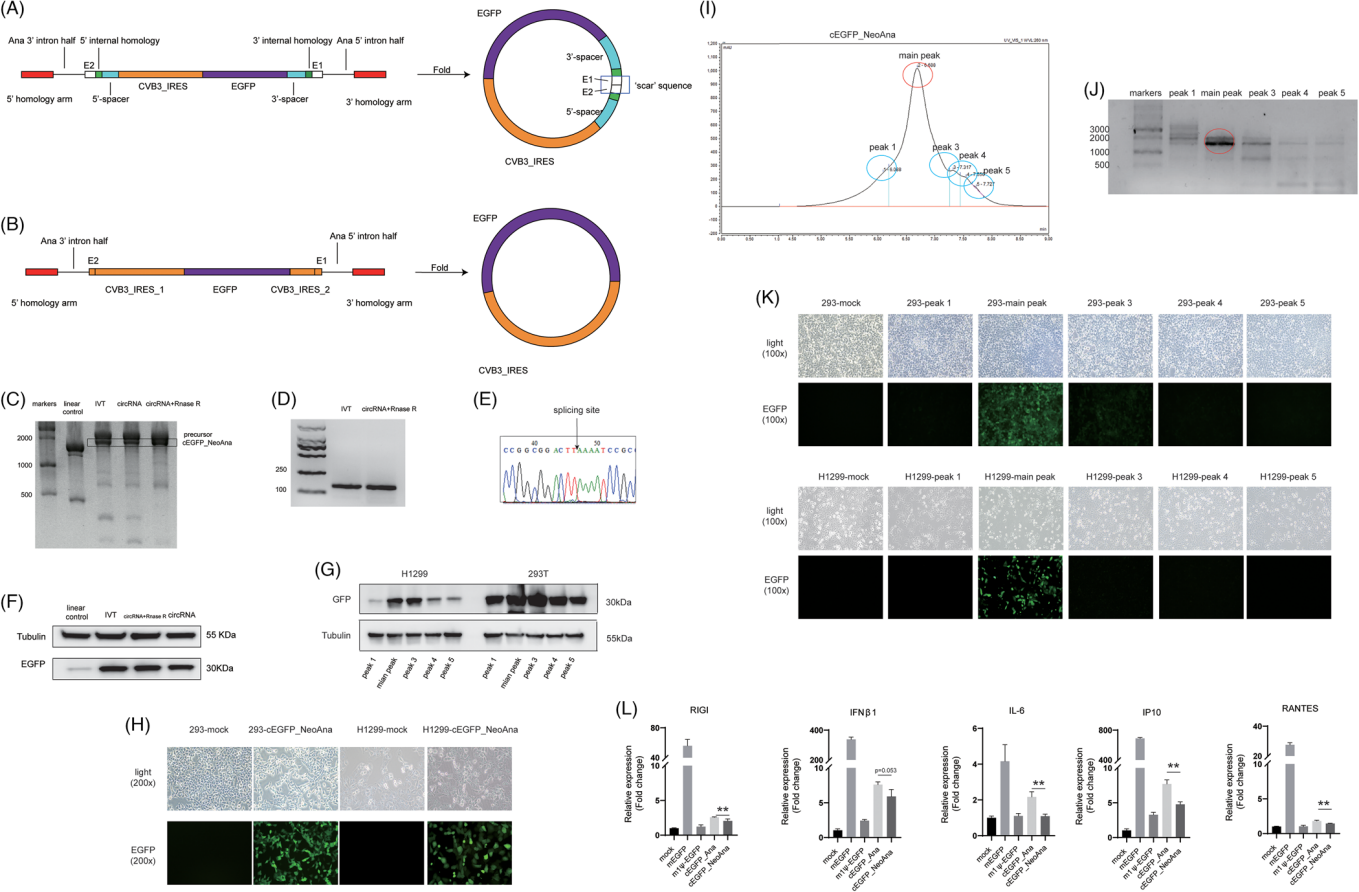
The design of group I permuted intron‐exon splicing systems of NeoAna. (A) The schematic graph of Ana (Anabaena). (B) The schematic graph of NeoAna. (C) The results of agarose gel electrophoresis showed cEGFP_NeoAna. (D) The PCR amplification products of splicing sites. (E) The arrow directly showed the splicing site. (F) Western blots showed the expression of green fluorescent protein (GFP). (G) Western blots showed the expression of GFP in each peak of high‐performance liquid chromatography (HPLC). (H) cEGFP_NeoAna expression in H1299 and 293 cells. (I) The schematic graph of HPLC of cEGFP_NeoAna. (J) The results of agarose gel electrophoresis showed the HPLC of cEGFP_NeoAna. (K) GFP of each peak of HPLC in H1299 and 293 cells. (L) The makers expression of innate immunity (^**^ *p* < .01).

The well‐known tumour suppressor, PTEN is a negative regulator of epidermal growth factor receptor (EGFR) signalling pathway,^4^ and PTEN protein expression is often lost in lung cancer.^5^ Thus, restoring PTEN expression might reverse EGFR‐TKI resistance.^6^ We synthesised PTEN protein template with NeoAna system (Figure S3B‒E) and long‐lasting PTEN protein expression was observed. Osimertinib‐resistant cells were established in HCC827 and PC9 cells, since they harbour EGFR exon 19 deletion (Figure S4A‒E).

Cell Counting Kit‐8 (CCK‐8), colony formation and 5‐ethynyl‐2'‐deoxyuridine (EdU) assays showed that cPTEN_NeoAna increased sensitivity to Osimertinib compared with control group (Figures 2A‒D, S4A and S3D). Annexin V‐FITC and TUNEL assay both confirmed that apoptosis rate was increased by elevating the concentration of Osimertinib and the transfection of cPTEN_NeoAna (Figures 2E,F and S5) in PC9 Osimertinib‐resistance (PC9OR) and HCC827 Osimertinib‐resistance (HCC827OR) cells.

**FIGURE 2:**
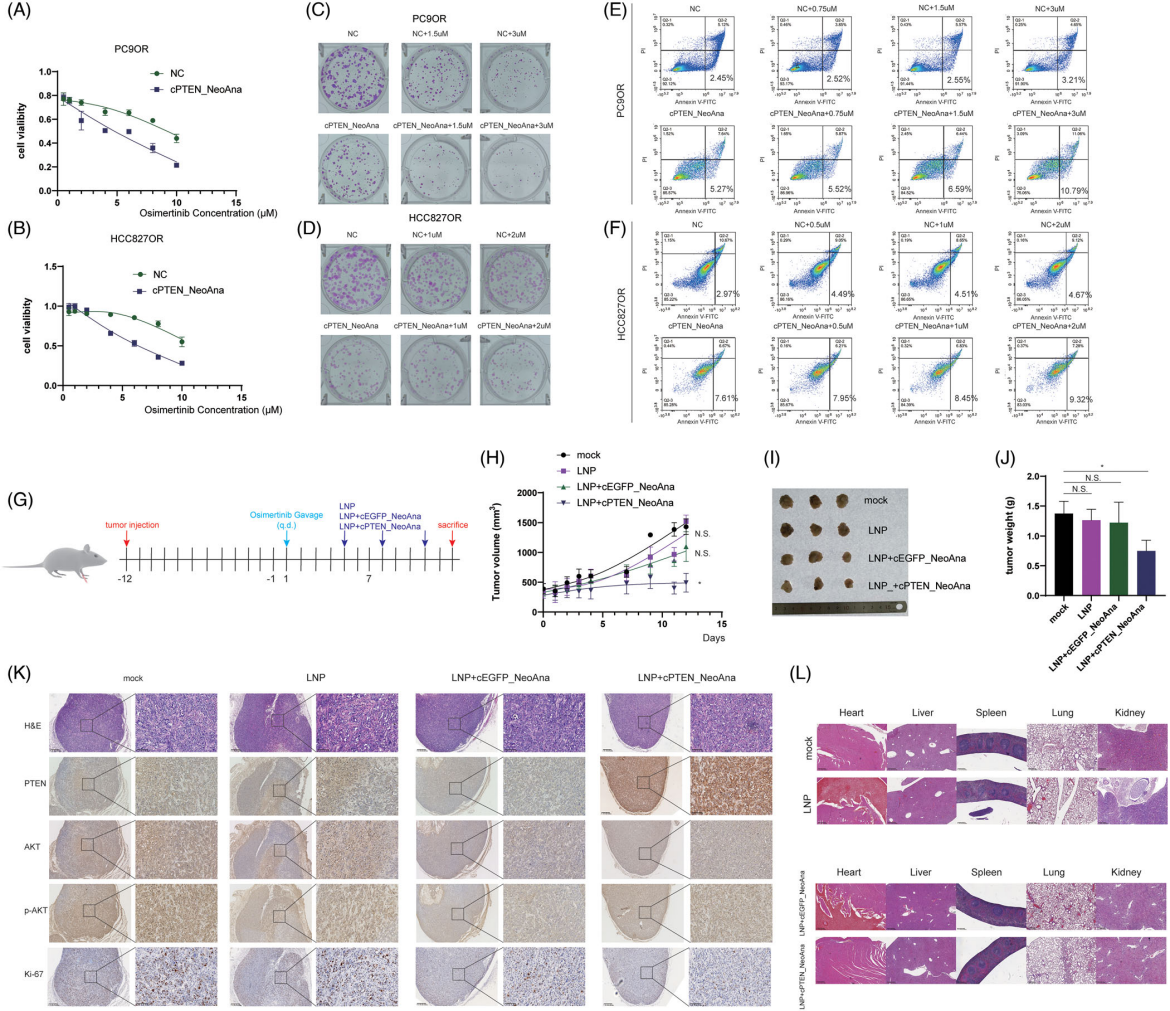
cPTEN_NeoAna can elevate non‐small cell lung cancer cells to Osimertinib sensitivity. CCK‐8 results showed cells with cPTEN_NeoAna is more sensitive to Osimertinib in PC9 Osimertinib‐resistance (PC9OR) (A) and HCC827 Osimertinib‐resistance (HCC827OR) cells (B). Colony formation assays showed that cells with cPTEN_NeoAna is more sensitive to Osimertinib in PC9OR (C) and HCC827OR (D). The apoptosis rate is increasing by the transfection of cPTEN_NeoAna in PC9OR (E) and HCC827OR (F). (G) The schematic graph of animal experiments. (H) The growth curve of tumour volume in different four groups. (I and J) Lipid nanoparticle (LNP) + cPTEN_NeoAna had smallest tumour volumes. (K) The representative photos of hematoxylin‐eosin (HE), phosphatase and tensin homologue deleted on chromosome 10 (PTEN), Protein Kinase B (AKT), p‐AKT and Ki‐67. (L) The representative photographs of main organ.

The cEGFP_NeoAna was encapsulated by lipid nanoparticles (LNP) to form the stable complex and observed under electron microscope (Figure S6A). The average diameter of LNP_ cEGFP_NeoAna was measured to be 90.6 nm. The LNP encapsulation rates of cEGFP_NeoAna and cPTEN_NeoAna are 91.8% and 90.6%, respectively. Then, we transfected LNP_cEGFP_NeoAna into A549 and H1299 cells, and the green fluorescence was observed (Figure S6C,D). The xenograft mice models were built with PC9OR cells and cPTEN_NeoAna was administered through intratumour injection (Figure 2G). Mice in the cPTEN_NeoAna group had lowest tumour volume, Ki‐67 expression and p‐AKT expression (Figure 2H‒K). Besides, we found both LNP and cPTEN_NeoAna are non‐toxic (Figures 2L and S4E). Thus, these data demonstrated that cPTEN_NeoAna can effectively increase sensitivity to Osimertinib of NSCLC cells in vitro and in vivo.

We synthesised m1ψ‐PTEN, cPTEN_Ana and cPTEN_NeoAna (Figure S2G) and treated cells with these RNAs in combination with Osimertinib. Then, CCK‐8 and colony formation assays showed that cPTEN_NeoAna was more effective than cPTEN_Ana and m1ψ‐PTEN (Figure 3A‒D), although with no significant statistical difference. The apoptosis rate was highest in cPTEN_NeoAna group and increased along with Osimertinib concentration (Figure 3E,F). Taken together, these results illustrated that cells with cPTEN_NeoAna plus Osimertinib had lowest proliferation ability and highest apoptosis rates compared with other groups. Then, mouse model bearing xenograft tumour was established to assess the therapeutic efficacy of m1ψ‐PTEN, cPTEN_Ana and cPTEN_NeoAna in vivo (Figure 3G). We observed that the tumour volume of cPTEN_NeoAna is smallest among four groups, which was in line with in vitro experiments (Figure 3H‒J). The results of immunohistochemistry illustrated that PTEN was successfully restored in the three groups (Figure 3K). The expression of p‐AKT and Ki‐67 was lowest in cPTEN_NeoAna. We found that LNP and engineered RNA are non‐toxic (Figure S6G).

**FIGURE 3:**
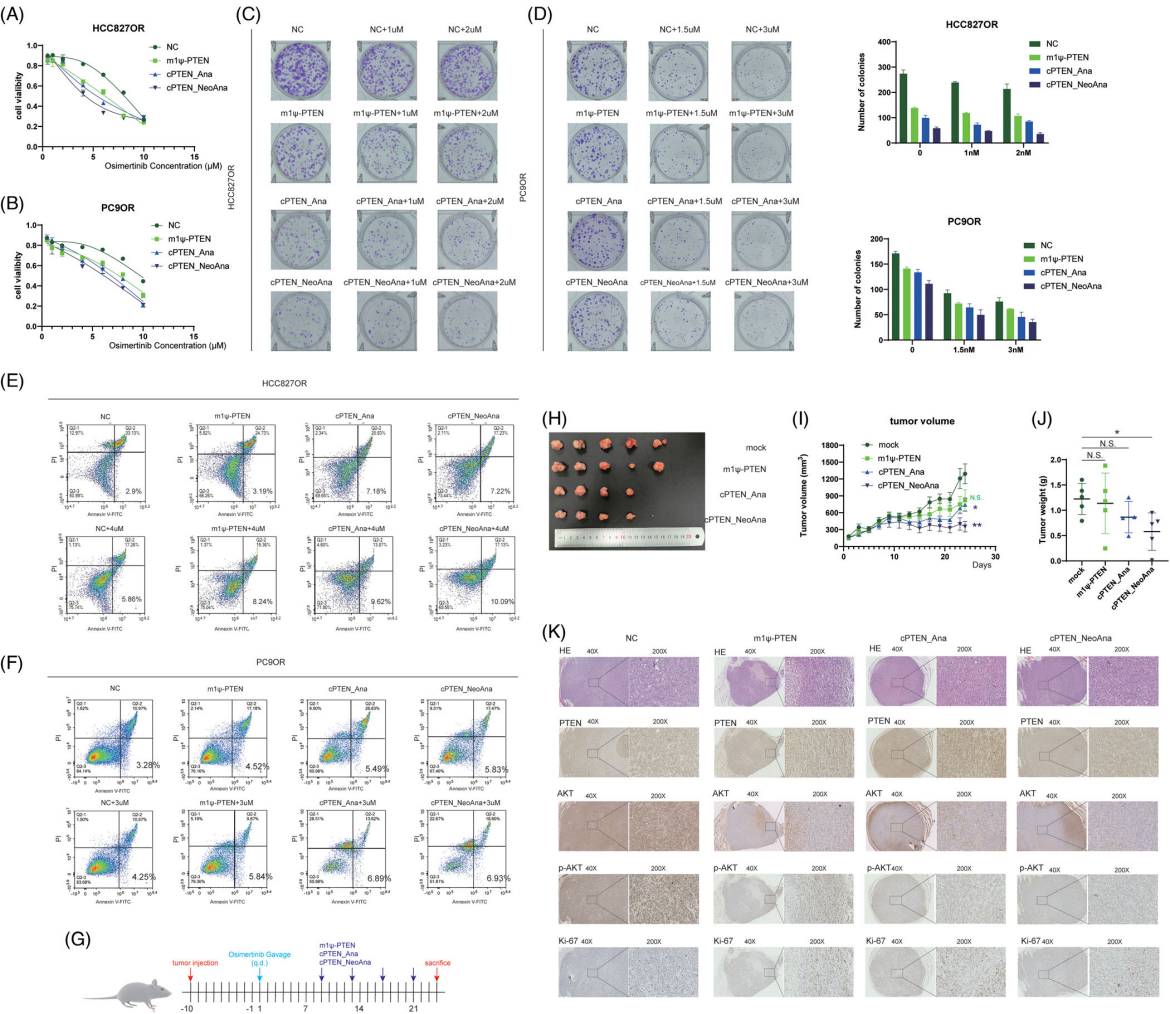
The efficacy of cPTEN_NeoAna to elevate non‐small cell lung cancer (NSCLC) to Osimertinib sensitivity is slightly superior to cPTEN_Ana and m1ψ‐PTEN. CCK‐8 results showed the proliferative ability of cells of cPTEN_NeoAna is slightly inferior to those of cPTEN_Ana and m1ψ‐PTEN in HCC827 Osimertinib‐resistance (HCC827OR) (A) and PC9 Osimertinib‐resistance (PC9OR) cells (B). Colony formation assays (C and D) also confirmed that finding. The apoptosis rate is highest in cells of cPTEN_NeoAna compared with those of cPTEN_Ana and m1ψ‐PTEN in HCC827OR (E) and PC9OR (F). (G) The schematic graph of animal experiments. (H and J) cPTEN_NeoAna had smallest tumour volumes. (I) The growth curve of tumour volume in different four groups. (K) The representative photos of HE, phosphatase and tensin homologue deleted on chromosome 10 (PTEN), AKT, p‐AKT and Ki‐67.

Compared with PC9 and HCC827 cells, PTEN protein was slightly decreased in PC9OR and HCC827OR cells. When PTEN protein expression was restored, p‐AKT and Kirsten rats arcomaviral oncogene homolog (KRAS) were significantly down‐regulated (Figure 4A). Simultaneously, the expression of p‐AKT but not KRAS decreased in an Osimertinib concentration‐dependent manner. Then, we performed RNA‐seq to reveal the potential mechanisms of PTEN enhancing Osimertinib sensitivity (Tables S2 and S3; Figures 4C,D and S7). Herein, we found that there were 64 common genes between the up‐regulated differentially expression genes (DEGs) of PC9OR versus PC9 and the down‐regulated DEGs of PC9OR_ cPTEN_NeoAna versus PC9OR (Figure 4E). Glutathione peroxidase 2 (GPX2), transient receptor potential cation channel subfamily V member 4 (TRPV4) and aldo‐keto reductase family 1 member C2 (AKR1C2) drew our attention. The RT‐qPCR results showed that only the expression of AKR1C2 was in line with RNA‐seq (Figure 4F,G). Compared with treatment‐naïve cells, AKR1C2 protein was increased in PC9OR and HCC827OR cells and subsequently decreased when transfected with cPTEN_NeoAna (Figure 4H,I). Co‐immunoprecipitation assays revealed that AKR1C2 could be immunoprecipitated by PTEN protein and vice versa (Figure 4J). Previous study reported that AKR1C2 could promote drug resistance of cancer cells by eliminating reactive oxygen species (ROS).^7^ Accordingly, we found that ROS level increased significantly after cPTEN_NeoAna treatment in PC9OR and HCC827OR cells but not Osimertinib treatment (Figures 4K,L and S8).

**FIGURE 4:**
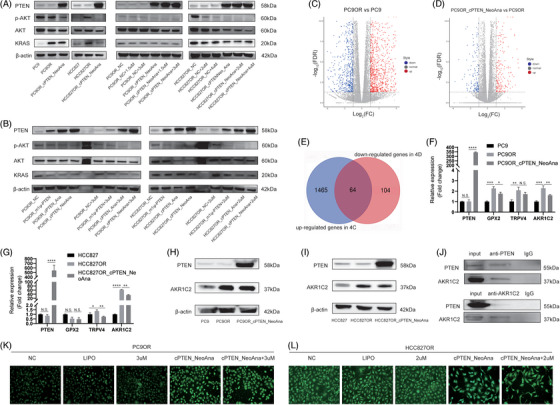
Phosphatase and tensin homologue deleted on chromosome 10 (PTEN) could oppose Phosphatidylinositol‐4,5‐bisphosphate 3‐kinase (PI3K)‒AKT signal pathway and decrease the expression of KRAS. (A and B) Western blots showed that different groups of PTEN, AKT, p‐AKT, KRAS and β‐actin. Volcano plots showed the differentially expression genes (DEGs) in the group of PC9 Osimertinib‐resistance (PC9OR) versus PC9 (C) and PC9OR_cPTEN_NeoAna versus PC9OR (D). (E) Venn diagram plot showed there is 64 common genes in two groups. (F and G) The expression of PTEN, glutathione peroxidase 2 (GPX2), transient receptor potential cation channel subfamily V member 4 (TRPV4) and aldo‐keto reductase family 1 member C2 (AKR1C2) in RNA level in PC9OR and HCC827 Osimertinib‐resistance (HCC827OR) cells. (H and I) Western blot showed PTEN and AKR1C2 protein expression in PC9OR and HCC827OR cells. (J) Co‐immunoprecipitation assay showed that PTEN can mutually interact with AKR1C2 in HCC827OR cells. (K and L) Intracellular reactive oxygen species (ROS) level detection in different cells. 2 µM/3 µM, Osimertinib concentration. N.S., *p* > .05; ^*^ *p* < .05; ^**^ *p* < .01; ^***^ *p* < .001; ^****^ *p* < .0001.

Besides, our strategy also successfully restored PTEN expression in colon cancer cells, DLD1 and DLD1 PTEN^−/−^ (Figure S9A). CCK‐8 and colony formation assays showed that the proliferative ability of DLD1 PTEN^−/−^ cells was compromised after restoring PTEN expression with cPTEN_NeoAna (Figure S9B,C).

Overall, our NeoAna circRNA platform could synthesise circRNA without scar sequences in the final products. Restoring PTEN expression with NeoAna could be a promising strategy to overcome Osimertinib resistance in NSCLC. Therefore, NeoAna circRNA platform may be an alternative choice for mRNA and can be widely used in RNA‐based therapeutics.

## AUTHOR CONTRIBUTIONS

Mantang Qiu contributed to the study conception and design. Material preparation, data collection and analysis were performed by Haoran Li, Zheng Liu, Shaoyi Chen, Jingsheng Cai and Peiyu Wang. Haoran Li and Mantang Qiu wrote and revised the manuscript. Mantang Qiu and Kezhong Chen provided funding for this manuscript. All authors read and approved the final manuscript.

## CONFLICT OF INTEREST STATEMENT

M.Q., H.L., Z.L., and J.C. have applied for the patent (202310162756.5) related to the NeoAna system. All other authors declared no competing interests.

## ETHICS STATEMENT

This study was approved by the Ethics Committee of Peking University People's Hospital (2022PHE073). Studies on animals were conducted in accordance with relevant guidelines and regulations and were approved by the Animal Research Ethics Committee of Peking University People's Hospital. All methods were carried out in accordance with relevant guidelines and regulations.

## Supporting information

FIGURE S1. Predictive secondary structure of NeoAna.
FIGURE S2. Results of agarose gel electrophoresis showed m1ψ‐EGFP, cEGFP_Ana and cEGFP_NeoAna (A). The EGFP expression of m1ψ‐EGFP, cEGFP_Ana and cEGFP_NeoAna in 293 (B) and H1299 cells (C).
FIGURE S3. Characterisations of cPTEN_NeoAna. (A) The results of agarose gel electrophoresis showed the stability of cEGFP_NeoAna and cEGFP_Ana. (B) The results of agarose gel electrophoresis showed cPTEN_NeoAna. (C) The PCR amplification products of splicing sites. (D) The schematic graph of high‐performance liquid chromatography (HPLC) of cPTEN_NeoAna. (E) The arrow directly showed the splicing site. (F) Western blots showed the expression of phosphatase and tensin homologue deleted on chromosome 10 (PTEN) in H1299 cells. (G) The results of agarose gel electrophoresis showed the HPLC of m1ψ‐PTEN, cPTEN_Ana and cPTEN_NeoAna.
FIGURE S4. HCC827 Osimertinib‐resistance (HCC827OR) and PC9 Osimertinib‐resistance (PC9OR) cells were resistance to Osimertinib. (A) The mutation sites of HCC827, HCC827OR, PC9 and PC9OR. (B and C) CCK‐8 showed HCC827OR and PC9OR were resistance to Osimertinib compared with HCC827 and PC9. (D) LIPO and cEGFP_NeoAna had no significant effect on cells. (E) The most suitable transfection of RNA volume was explored.
FIGURE S5. EdU and TUNEL results of HCC827 Osimertinib‐resistance (HCC827OR) and PC9 Osimertinib‐resistance (PC9OR) cells.
FIGURE S6. (A) Representative photograph of electronic microscope of lipid nanoparticle (LNP). (B) The diameter of LNP. (C and D) The expression of LNP_cEGFP_NeoAna in A549 and H1299. (E and F) The curve of body weight in animal experiments. (G) The main organs were not changed in m1ψ‐PTEN, cPTEN_Ana and cPTEN_NeoAna compared to negative control (NC).
FIGURE S7. Gene Ontology (GO) analysis and Kyoto Encyclopedia of Genes and Genomes (KEGG) pathway analysis of RNA sequencing. GO analysis in group of PC9 Osimertinib‐resistance (PC9OR) versus PC9 (A) and PC9OR_cPTEN_NeoAna versus PC9OR (B). KEGG pathway analysis in group of PC9OR versus PC9 (C) and PC9OR_cPTEN_NeoAna versus PC9OR (D).
FIGURE S8. The mean fluorescence intensity (MFI) of intracellular reactive oxygen species (ROS) level corresponding to Figure 4K (A) and Figure 4L (B).
FIGURE S9. cPTEN_NeoAna could decrease the proliferation ability of DLD1 PTEN^−/−^ cells. Western blots confirmed PTEN protein expression in DLD1 PTEN^−/−^ cells transfected with cPTEN_NeoAna (A). CCK‐8 (B) and colony formation (C) assays showed cPTEN_NeoAna could decrease the proliferation ability of DLD1 PTEN^−/−^ cells. ^****^ *p* < .0001.

TABLE S1. Primers, primary antibodies and sequences included in this study.

TABLE S2. List of up‐regulated genes in the group of PC9 Osimertinib‐resistance (PC9OR) versus PC9.

TABLE S3. List of down‐regulated genes in the group of PC9OR_cPTEN_NeoAna versus PC9 Osimertinib‐resistance (PC9OR).

Supporting Information

## Data Availability

The datasets generated and/or analysed during the current study are available from the corresponding author upon reasonable request.
