# Supplementary material for: Restoring expression of tumour suppressor PTEN by engineered circular RNA‐enhanced Osimertinib sensitivity in non‐small cell lung cancer
Source: Clin Transl Med. 2024 Aug 21;14(8):e1792. doi: 10.1002/ctm2.1792 (PMC11337465; doi:10.1002/ctm2.1792)
Supplement: Supplementary file 1 — FIGURE S1. Predictive secondary structure of NeoAna. FIGURE S2. Results of agarose gel electrophoresis showed m1ψ‐EGFP, cEGFP_Ana and cEGFP_NeoAna (A). The EGFP expression of m1ψ‐EGFP, cEGFP_Ana and cEGFP_NeoAna in 293 (B) and H1299 cells (C). FIGURE S3. Characterisations of cPTEN_NeoAna. (A) The results of agarose gel electrophoresis showed the stability of cEGFP_NeoAna and cEGFP_Ana. (B) The results of agarose gel electrophoresis showed cPTEN_NeoAna. (C) The PCR amplification products of splicing sites. (D) The schematic graph of high‐performance liquid chromatography (HPLC) of cPTEN_NeoAna. (E) The arrow directly showed the splicing site. (F) Western blots showed the expression of phosphatase and tensin homologue deleted on chromosome 10 (PTEN) in H1299 cells. (G) The results of agarose gel electrophoresis showed the HPLC of m1ψ‐PTEN, cPTEN_Ana and cPTEN_NeoAna. FIGURE S4. HCC827 Osimertinib‐resistance (HCC827OR) and PC9 Osimertinib‐resistance (PC9OR) cells were resistance to Osimertinib. (A) The mutation sites of HCC827, HCC827OR, PC9 and PC9OR. (B and C) CCK‐8 showed HCC827OR and PC9OR were resistance to Osimertinib compared with HCC827 and PC9. (D) LIPO and cEGFP_NeoAna had no significant effect on cells. (E) The most suitable transfection of RNA volume was explored. FIGURE S5. EdU and TUNEL results of HCC827 Osimertinib‐resistance (HCC827OR) and PC9 Osimertinib‐resistance (PC9OR) cells. FIGURE S6. (A) Representative photograph of electronic microscope of lipid nanoparticle (LNP). (B) The diameter of LNP. (C and D) The expression of LNP_cEGFP_NeoAna in A549 and H1299. (E and F) The curve of body weight in animal experiments. (G) The main organs were not changed in m1ψ‐PTEN, cPTEN_Ana and cPTEN_NeoAna compared to negative control (NC). FIGURE S7. Gene Ontology (GO) analysis and Kyoto Encyclopedia of Genes and Genomes (KEGG) pathway analysis of RNA sequencing. GO analysis in group of PC9 Osimertinib‐resistance (PC9OR) versus PC9 (A) and PC9OR_cPTEN_NeoAna versus [file CTM2-14-e1792-s004.docx]

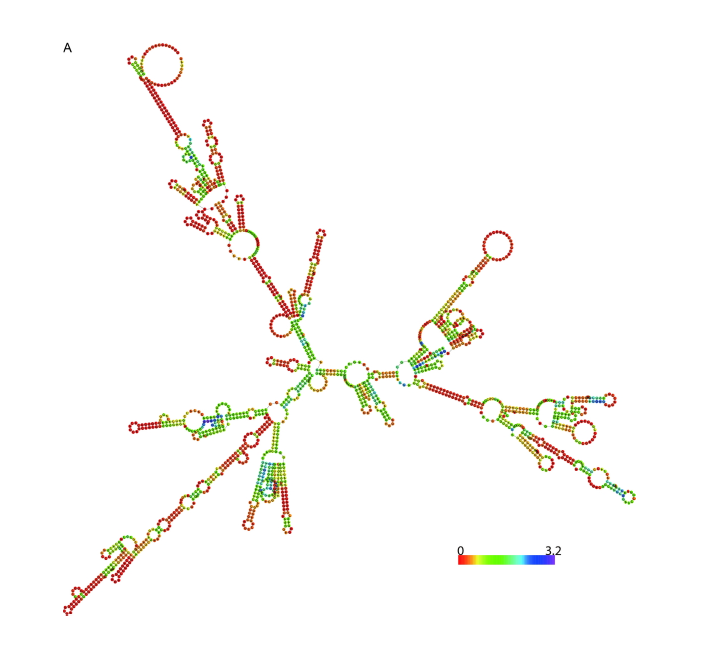


Figure S1. The predictive secondary structure of NeoAna.


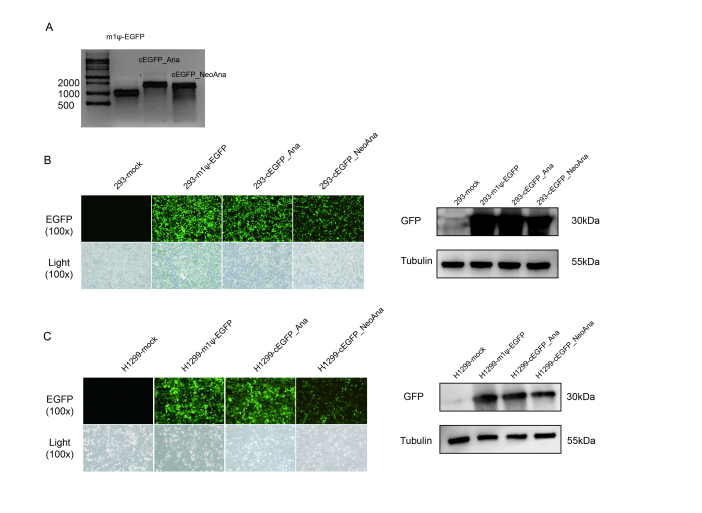


Figure S2. The results of agarose gel electrophoresis showed m1ψ-EGFP, cEGFP_Ana and cEGFP_NeoAna (A). The EGFP expression of m1ψ-EGFP, cEGFP_Ana and cEGFP_NeoAna in 293 (B) and H1299 cells (C).


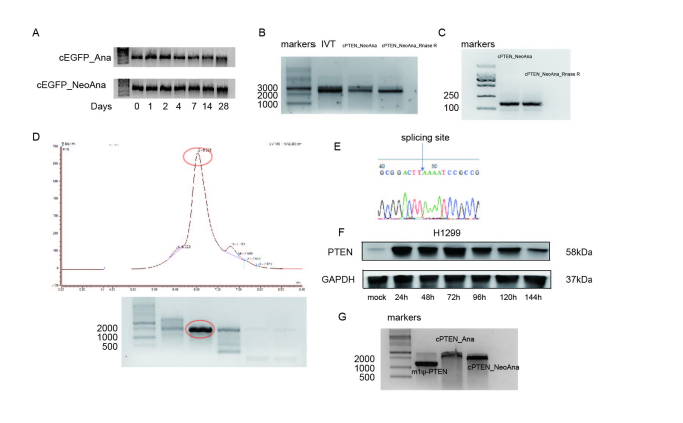


Figure S3.The characterizations of cPTEN_NeoAna. (A) the results of agarose gel electrophoresis showed the stability of cEGFP_NeoAna and cEGFP_Ana. (B) (A) the results of agarose gel electrophoresis showed cPTEN_NeoAna. (C) the PCR amplification products of splicing sites. (D) the schematic graph of HPLC of cPTEN_NeoAna. (E) the arrow directly showed the splicing site. (F) western blots showed the expression of PTEN in H1299 cells. (F) western blots showed the expression of GFP in each peak of HPLC. (G) the results of agarose gel electrophoresis showed the HPLC of m1ψ-PTEN, cPTEN_Ana and cPTEN_NeoAna.


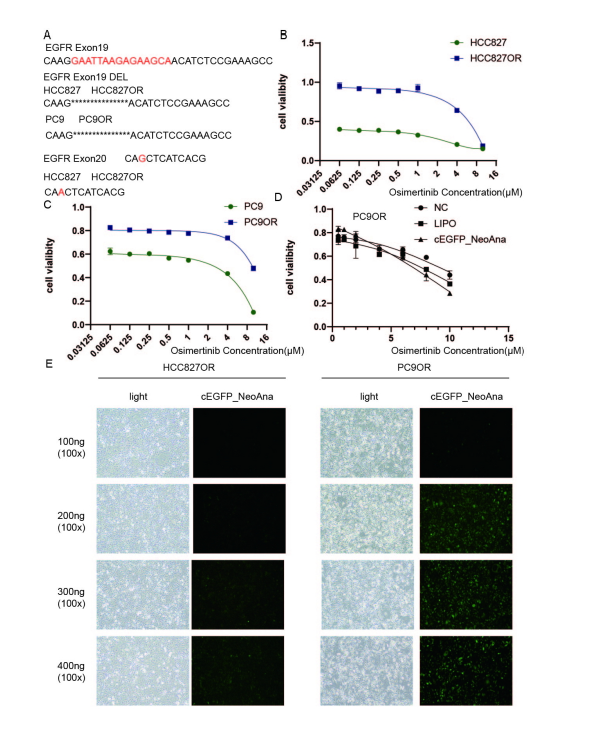


Figure S4. HCC827OR and PC9OR were resistance to Osimertinib. (A) the mutation sites of HCC827, HCC827OR, PC9 and PC9OR. (B, C) CCK-8 showed HCC827OR and PC9OR were resistance to Osimertinib compared with HCC827 and PC9. (D) LIPO and cEGFP_NeoAna had no significant effect on cells. (E) the most suitable transfection of RNA volume was explored.


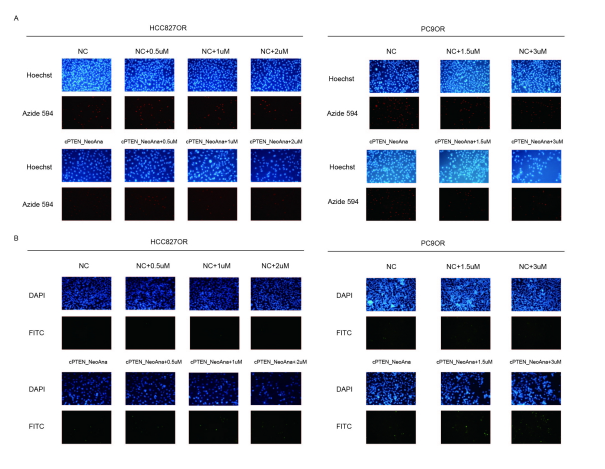


Figure S5. The EdU and TUNEL results of HCC827OR and PC9OR.


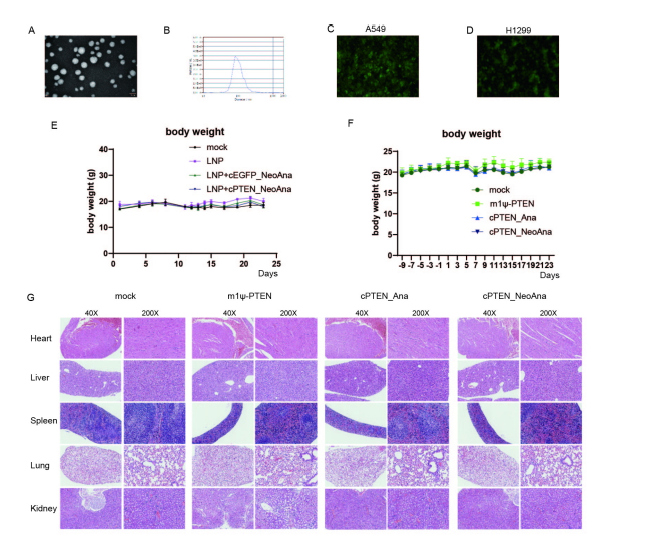


Figure S6. (A) the representative photo of electronic microscope of lipid nanoparticle (LNP). (B) the diameter of LNP. (C, D) the expression of LNP_cEGFP_NeoAna in A549 and H1299. (E, F) the curve of body weight in animal experiments. (G) the main organs were not changed in m1ψ-PTEN, cPTEN_Ana and cPTEN_NeoAna compared to negative control (NC).


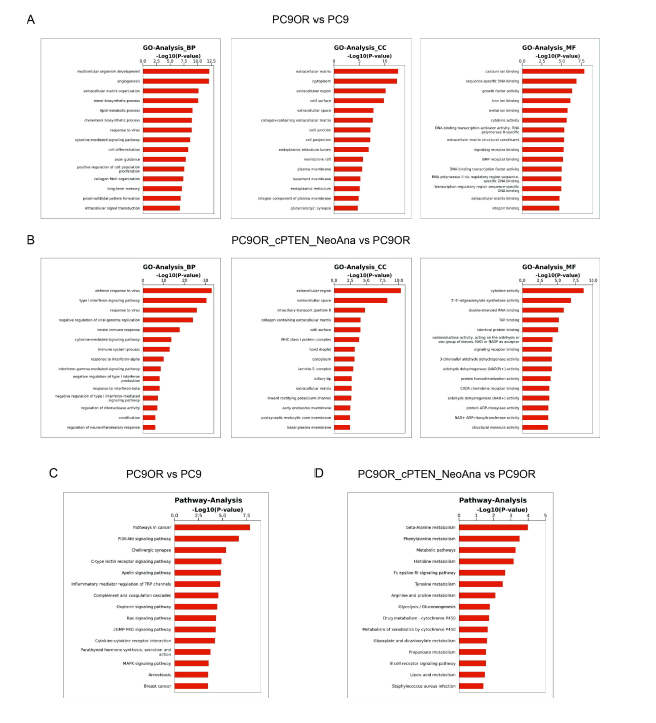


Figure S7. GO analysis and KEGG pathway analysis of RNA sequencing. GO analysis in group of PC9OR vs. PC9 (A) and PC9OR_cPTEN_NeoAna vs. PC9OR (B). KEGG pathway analysis in group of PC9OR vs. PC9 (C) and PC9OR_cPTEN_NeoAna vs. PC9OR (D).


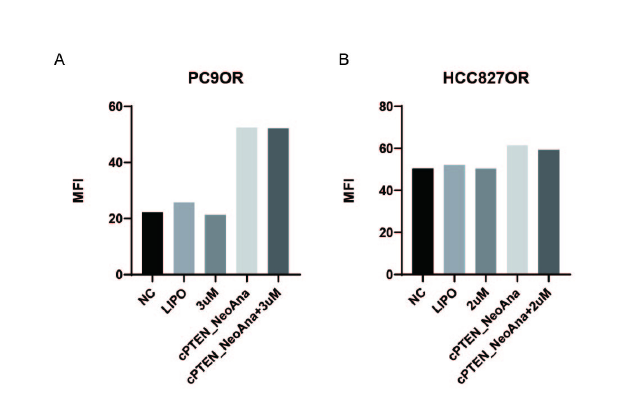


Figure S8. The Mean Fluorescence Intensity (MFI) of intracellular ROS level corresponding to Figure 4K (A) and 4L (B).


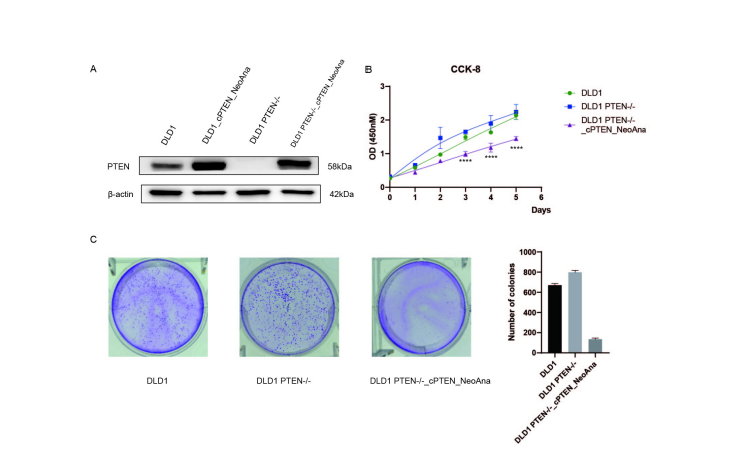


Figure S9. cPTEN_NeoAna could decrease the proliferation ability of DLD1 PTEN^-/-^ cells. Western blots confirmed PTEN protein expression in DLD1 PTEN^-/-^ cells transfected with cPTEN_NeoAna (A). CCK-8 (B) and colony formation (C) assays showed cPTEN_NeoAna could decrease the proliferation ability of DLD1 PTEN^-/-^ cells. ****, p<0.0001.
