# Supplementary material for: Restoring expression of tumour suppressor PTEN by engineered circular RNA‐enhanced Osimertinib sensitivity in non‐small cell lung cancer
Source: Clin Transl Med. 2024 Aug 21;14(8):e1792. doi: 10.1002/ctm2.1792 (PMC11337465; doi:10.1002/ctm2.1792)
Supplement: Supplementary file 2 — TABLE S1. Primers, primary antibodies and sequences included in this study. [file CTM2-14-e1792-s001.docx]

Table S1. The primers, primary antibodies and sequences included in this study.

1. Primers

| RIGI-F | TGTGGGCAATGTCATCAAAA |
| --- | --- |
| RIGI-R | GAAGCACTTGCTACCTCTTGC |
| IP10-F | GCCTCTCCCATCACTTCCCTAC |
| IP10-R | GAAGCAGGGTCAGAACATCCAC |
| IFNβ-1-F | CTCTCCTGTTGTGCTTCTCC |
| IFNβ-1-R | GTCAAAGTTCATCCTGTCCTTG |
| IL6-F | GCAATAACCACCCCTGACCC |
| IL6-R | AAGCTGCGCAGAATGAGATG |
| RANTES-R | GGCAGCCCTCGCTGTCATCCTCA |
| RANTES-F | CTTGATGTGGGCACGGGGCAGTG |
| GADPH-F | CGCTCTCTGCTCCTCCTGTTC |
| GADPH-R | ATCCGTTGACTCCGACCTTCAC |
| NeoAna-J-cEGFP-F | GGATCACTCTCGGCATGGAC |
| NeoAna-J-cEGFP-R | GCTAGCGCCCAATGGTAAGA |
| NeoAna-F | TGCATCTAGATTAATACGACTCACT |
| NeoAna-R | CTAGATATGCTGTTATCCGTCGATT |
| NeoAna-J-cPTEN-F | ATGAACCTTTTGATGAAGATCAGC |
| NeoAna-J-cPTEN-R | TGCTAGCGCCCAATGGTAAG |

1. Primary antibody

| Antibody | Application | Company |
| --- | --- | --- |
| PTEN | IHC/WB | Proteintech, 22034-1-AP |
| AKT | IHC/WB | Proteintech, 10176-2-AP |
| p-AKT | IHC/WB | Proteintech, 28731-1-AP |
| Ki-67 | IHC | Abcam, ab16667 |
| KRAS | WB | Proteintech, 12063-1-AP |
| GAPDH | WB | Proteintech, 60004-1-Ig |
| β-actin | WB | Proteintech, 60008-1-Ig |
| β-tubulin | WB | TDYbio, TDY043 |
| GFP | WB | Proteintech, 50430-2-AP |

1. Sequences

| T7 promoter | TAATACGACTCACTATAGGG |
| --- | --- |
| 3'UTR | GGGAAATAAGAGAGAAAAGAAGAGTAAGAAGAAATATAAGAGCCACC |
| 5'UTR | GCTGGAGCCTCGGTGGCCATGCTTCTTGCCCCTTGGGCCTCCCCCCAGCCCCTCCTCCCCTTCCTGCACCCGTACCCCCGTGGTCTTTGAATAAAGTCTGAGTGGGCGGCA |
| PTEN CDS | ATGACAGCCATCATCAAAGAGATCGTTAGCAGAAACAAAAGGAGATATCAAGAGGATGGATTCGACTTAGACTTGACCTATATTTATCCAAACATTATTGCTATGGGATTTCCTGCAGAAAGACTTGAAGGCGTATACAGGAACAATATTGATGATGTAGTAAGGTTTTTGGATTCAAAGCATAAAAACCATTACAAGATATACAATCTTTGTGCTGAAAGACATTATGACACCGCCAAATTTAATTGCAGAGTTGCACAATATCCTTTTGAAGACCATAACCCACCACAGCTAGAACTTATCAAACCCTTTTGTGAAGATCTTGACCAATGGCTAAGTGAAGATGACAATCATGTTGCAGCAATTCACTGTAAAGCTGGAAAGGGACGAACTGGTGTAATGATATGTGCATATTTATTACATCGGGGCAAATTTTTAAAGGCACAAGAGGCCCTAGATTTCTATGGGGAAGTAAGGACCAGAGACAAAAAGGGAGTAACTATTCCCAGTCAGAGGCGCTATGTGTATTATTATAGCTACCTGTTAAAGAATCATCTGGATTATAGACCAGTGGCACTGTTGTTTCACAAGATGATGTTTGAAACTATTCCAATGTTCAGTGGCGGAACTTGCAATCCTCAGTTTGTGGTCTGCCAGCTAAAGGTGAAGATATATTCCTCCAATTCAGGACCCACACGACGGGAAGACAAGTTCATGTACTTTGAGTTCCCTCAGCCGTTACCTGTGTGTGGTGATATCAAAGTAGAGTTCTTCCACAAACAGAACAAGATGCTAAAAAAGGACAAAATGTTTCACTTTTGGGTAAATACATTCTTCATACCAGGACCAGAGGAAACCTCAGAAAAAGTAGAAAATGGAAGTCTATGTGATCAAGAAATCGATAGCATTTGCAGTATAGAGCGTGCAGATAATGACAAGGAATATCTAGTACTTACTTTAACAAAAAATGATCTTGACAAAGCAAATAAAGACAAAGCCAACCGATACTTTTCTCCAAATTTTAAGGTGAAGCTGTACTTCACAAAAACAGTAGAGGAGCCGTCAAATCCAGAGGCTAGCAGTTCAACTTCTGTAACACCAGATGTTAGTGACAATGAACCTGATCATTATAGATATTCTGACACCACTGACTCTGATCCAGAGAATGAACCTTTTGATGAAGATCAGCATACACAAATTACAAAAGTCTGA |
| 5’homology arm | AGACCCTCGACCGTCGATTGTCCACTGGTC |
| Ana 3' intron half | AACAATAGATGACTTACAACTAATCGGAAGGTGCAGAGACTCGACGGGAGCTACCCTAACGTCAAGACGAGGGTAAAGAGAGAGTCCAATTCTCAAAGCCAATAGGCAGTAGCGAAAGCTGCAAGAGAATG |
| CVB3_IRES_1 | aaaaTcCGCCGGAAACGCAATAGCCGAAAAACAAAAAACtctta CCATTGGGCGCTAGCACTCTGGTATCACGGTACCTTTGTGCGCCTGTTTTATACCCCCTCCCCCAACTGTAACTTAGAAGTAACACACACCGATCAACAGTCAGCGTGGCACACCAGCCACGTTTTGATCAAGCACTTCTGTTACCCCGGACTGAGTATCAATAGACTGCTCACGCGGTTGAAGGAGAAAGCGTTCGTTATCCGGCCAACTACTTCGAAAAACCTAGTAACACCGTGGAAGTTGCAGAGTGTTTCGCTCAGCACTACCCCAGTGTAGATCAGGTCGATGAGTCACCGCATTCCCCACGGGCGACCGTGGCGGTGGCTGCGTTGGCGGCCTGCCCATGGGGAAACCCATGGGACGCTCTAATACAGACATGGTGCGAAGAGTCTATTGAGCTAGTTGGTAGTCCTCCGGCCCCTGAATGCGGCTAATCCTAACTGCGGAGCACACACCCTCAAGCCAGAGGGCAGTGTGTCGTAACGGGCAACTCTGCAGCGGAACCGACTACTTTGGGTGTCCGTGTTTCATTTTATTCCTATACTGGCTGCTTATGGTGACAATTGAGAGATCGTTACCATATAGCTATTGGATTGGCCATCCGGTGACTAATAGAGCTATTATATATCCCTTTGTTGGGTTTATACCACTTAGCTTGAAAGAGGTTAAAACATTACAATTCATTGTTAAGTTGAATACAGCAAA |
| CVB3_IRES_2 | tttatAAAAAACAAAACGGCTATTATGCGTTACCGGCGGActt |
| Ana 5' intron half | AAATAATTGAGCCTTAAAGAAGAAATTCTTTAAGTGGATGCTCTCAAACTCAGGGAAACCTAAATCTAGTTATAGACAAGGCAATCCTGAGCCAAGCCGAAGTAGTAATTAGTAAG |
| 3’homology arm | ACCAGTGGACAATCGACGGATAACAGCATATCTAG |
| EGFP CDS | ATGGTGAGCAAGGGCGAGGAGCTGTTCACCGGGGTGGTGCCCATCCTGGTCGAGCTGGACGGCGACGTAAACGGCCACAAGTTCAGCGTGTCTGGCGAGGGCGAGGGCGATGCCACCTACGGCAAGCTGACCCTGAAGTTCATCTGCACCACCGGCAAGCTGCCCGTGCCCTGGCCCACCCTCGTGACCACCCTGACCTACGGCGTGCAGTGCTTCAGCCGCTACCCCGACCACATGAAGCAGCACGACTTCTTCAAGTCCGCCATGCCCGAAGGCTACGTCCAGGAGCGCACCATCTTCTTCAAGGACGACGGCAACTACAAGACCCGCGCCGAGGTGAAGTTCGAGGGCGACACCCTGGTGAACCGCATCGAGCTGAAGGGCATCGACTTCAAGGAGGACGGCAACATCCTGGGGCACAAGCTGGAGTACAACTACAACAGCCACAACGTCTATATCATGGCCGACAAGCAGAAGAACGGCATCAAGGCGAACTTCAAGATCCGCCACAACATCGAGGACGGCAGCGTGCAGCTCGCCGACCACTACCAGCAGAACACCCCCATCGGCGACGGCCCCGTGCTGCTGCCCGACAACCACTACCTGAGCACCCAGTCCGCCCTGAGCAAAGACCCCAACGAGAAGCGCGATCACATGGTCCTGCTGGAGTTCGTGACCGCCGCCGGGATCACTCTCGGCATGGACGAGCTGTACAAGTAA |
