# Supplementary material for: Restoring expression of tumour suppressor PTEN by engineered circular RNA‐enhanced Osimertinib sensitivity in non‐small cell lung cancer
Source: Clin Transl Med. 2024 Aug 21;14(8):e1792. doi: 10.1002/ctm2.1792 (PMC11337465; doi:10.1002/ctm2.1792)
Supplement: Supplementary file 3 — TABLE S2. List of up‐regulated genes in the group of PC9 Osimertinib‐resistance (PC9OR) versus PC9. [file CTM2-14-e1792-s005.docx]

Table S2. The list of up-regulated genes in the group of PC9OR vs. PC9.

| gene | style | FDR | Pvalue | Fold Change | log2FC |
| --- | --- | --- | --- | --- | --- |
| CFH | up | 0.003733656 | 0.001126319 | 6.619639375 | 2.726752624 |
| SEMA3F | up | 3.30024E-74 | 2.22086E-76 | 4.249800159 | 2.087395002 |
| MYH16 | up | 1.236E-106 | 4.4187E-109 | 6.695240108 | 2.743135795 |
| HS3ST1 | up | 6.51772E-06 | 1.15682E-06 | 2.163946155 | 1.113664602 |
| HECW1 | up | 4.37255E-08 | 5.72861E-09 | 92.40613878 | 6.529916792 |
| CYP26B1 | up | 3.42123E-64 | 2.85987E-66 | 7.19249115 | 2.846491541 |
| MEOX1 | up | 0.043560619 | 0.01771544 | 3.146330459 | 1.653670205 |
| CRLF1 | up | 0.000106029 | 2.33229E-05 | 2.112656354 | 1.079058117 |
| TMEM98 | up | 1.91017E-19 | 9.3997E-21 | 8.852174969 | 3.146031967 |
| CX3CL1 | up | 0.026092841 | 0.009896102 | 5.348190101 | 2.419050747 |
| CCL26 | up | 0.000126425 | 2.81882E-05 | 2.927539766 | 1.549688768 |
| USH1C | up | 1.62408E-06 | 2.63067E-07 | 59.53561189 | 5.895680986 |
| ARHGAP44 | up | 0.012868188 | 0.004469126 | 2.743473787 | 1.456003792 |
| GAS7 | up | 2.54709E-29 | 7.27128E-31 | 17.30291891 | 4.112943528 |
| IL32 | up | 6.49525E-06 | 1.15112E-06 | 2.687140925 | 1.426071984 |
| IFFO1 | up | 2.18141E-12 | 1.85789E-13 | 4.396409339 | 2.136325718 |
| STAB1 | up | 0.006255587 | 0.002006578 | 6.820023845 | 2.769776783 |
| MRC2 | up | 5.93351E-23 | 2.33647E-24 | 3.292828601 | 1.719327418 |
| ABHD5 | up | 1.07027E-11 | 9.61056E-13 | 2.02752657 | 1.019720821 |
| EHD3 | up | 3.29698E-12 | 2.84094E-13 | 2.876642693 | 1.524386035 |
| POLR3B | up | 2.70973E-10 | 2.78366E-11 | 2.035308492 | 1.02524748 |
| DDX11 | up | 8.9104E-189 | 1.1243E-191 | 5.900038614 | 2.560724396 |
| MAMLD1 | up | 2.02964E-07 | 2.91839E-08 | 2.228958603 | 1.156369823 |
| CNTN1 | up | 2.9364E-117 | 9.7257E-120 | 46.63293522 | 5.543277335 |
| CYP24A1 | up | 2.04135E-14 | 1.45957E-15 | 18.32681509 | 4.195884185 |
| PRDM11 | up | 3.12539E-48 | 4.04209E-50 | 5.153635567 | 2.365590523 |
| SYT13 | up | 5.92247E-09 | 6.94966E-10 | 60.08259576 | 5.908875238 |
| SNAI2 | up | 1.66772E-54 | 1.8237E-56 | 5.873587834 | 2.554242031 |
| ADGRA2 | up | 1.49948E-15 | 9.57031E-17 | 4.020105742 | 2.007233449 |
| NRXN3 | up | 0.00617865 | 0.001980507 | 56.38738742 | 5.817300595 |
| CPS1 | up | 2.09951E-60 | 2.00889E-62 | 58.42957409 | 5.868626868 |
| FHL1 | up | 4.4673E-09 | 5.1599E-10 | 12.78491124 | 3.676370239 |
| DEF6 | up | 3.03011E-08 | 3.89179E-09 | 2.001973933 | 1.00142319 |
| PLEKHO1 | up | 5.44756E-06 | 9.55993E-07 | 2.956104154 | 1.563697102 |
| TOMM34 | up | 7.29281E-60 | 7.05471E-62 | 2.644543029 | 1.403018449 |
| KCNG1 | up | 6.7781E-102 | 2.6525E-104 | 6.319607456 | 2.659834948 |
| SH2D2A | up | 1.63826E-36 | 3.39348E-38 | 4.946522596 | 2.306414667 |
| TNFRSF1B | up | 1.74964E-20 | 8.04867E-22 | 6.715972584 | 2.747596341 |
| POU2F2 | up | 1.48705E-07 | 2.09922E-08 | 3.133069105 | 1.647576591 |
| ARNTL2 | up | 1.299E-278 | 5.4635E-282 | 6.665399968 | 2.736691449 |
| ANK1 | up | 3.57817E-17 | 2.01849E-18 | 6.590467085 | 2.720380717 |
| ARHGAP31 | up | 0.001287064 | 0.000349695 | 2.131408612 | 1.091807198 |
| METTL1 | up | 1.92457E-07 | 2.76124E-08 | 2.757314996 | 1.46326409 |
| HOXC8 | up | 0.000352655 | 8.59899E-05 | 3.14112036 | 1.651279225 |
| TLL1 | up | 9.17375E-05 | 1.98417E-05 | 8.807560314 | 3.138742449 |
| TNC | up | 9.8368E-53 | 1.11705E-54 | 3.722374999 | 1.896223402 |
| TG | up | 1.09523E-07 | 1.51609E-08 | 13.34518309 | 3.738247194 |
| CC2D2A | up | 7.27147E-05 | 1.54826E-05 | 3.403858007 | 1.767170856 |
| CELF2 | up | 3.54613E-05 | 7.13476E-06 | 9.303320277 | 3.217745694 |
| TNFRSF9 | up | 1.15827E-38 | 2.19829E-40 | 8.24182369 | 3.042963602 |
| DKK3 | up | 4.09541E-25 | 1.41459E-26 | 4.038260744 | 2.013734066 |
| LIMA1 | up | 4.37555E-44 | 6.41805E-46 | 2.300560646 | 1.201985489 |
| LAMC3 | up | 2.12015E-38 | 4.06842E-40 | 7.8199976 | 2.967168165 |
| ALX4 | up | 3.72543E-19 | 1.86262E-20 | 4.711290818 | 2.236122389 |
| MCF2L2 | up | 4.20747E-05 | 8.55606E-06 | 2.152599676 | 1.106080043 |
| LAMA3 | up | 8.38158E-23 | 3.33131E-24 | 2.053992878 | 1.038431179 |
| LY75 | up | 3.17846E-12 | 2.73213E-13 | 12.19250816 | 3.607923032 |
| CYFIP2 | up | 2.70126E-31 | 7.21435E-33 | 12.68170504 | 3.664676822 |
| TRAF1 | up | 1.23741E-05 | 2.29384E-06 | 3.673956229 | 1.877334438 |
| LAMC2 | up | 8.34277E-29 | 2.44744E-30 | 2.103917723 | 1.073078287 |
| RASGRF1 | up | 1.0281E-151 | 1.9459E-154 | 9.136512088 | 3.191643514 |
| TBXAS1 | up | 0.002152901 | 0.000613805 | 7.359920088 | 2.879690102 |
| BCAT1 | up | 0 | 0 | 13.27449342 | 3.7305849 |
| LZTS1 | up | 3.06356E-05 | 6.08976E-06 | 86.41698955 | 6.433243069 |
| MRPS35 | up | 3.603E-249 | 2.0836E-252 | 5.638666181 | 2.495353935 |
| DGAT2 | up | 0.000133585 | 2.99603E-05 | 2.047106244 | 1.03358598 |
| CA11 | up | 1.12817E-05 | 2.07769E-06 | 2.082680878 | 1.058441797 |
| INTS13 | up | 2.967E-130 | 7.9553E-133 | 3.808442522 | 1.929201122 |
| TM7SF3 | up | 1.5319E-247 | 9.6645E-251 | 6.115750921 | 2.612529651 |
| ATP2C2 | up | 2.31865E-33 | 5.65613E-35 | 3.694340006 | 1.88531665 |
| NGFR | up | 6.78218E-23 | 2.68135E-24 | 15.37979244 | 3.942964128 |
| FAR2 | up | 1.81611E-33 | 4.4016E-35 | 3.39684934 | 1.764197232 |
| ANKRD44 | up | 0.026102817 | 0.009904002 | 2.059081242 | 1.042000753 |
| COL17A1 | up | 1.80873E-68 | 1.41686E-70 | 163.4884803 | 7.353045174 |
| PRKCQ | up | 1.55389E-15 | 9.93389E-17 | 3.791567709 | 1.922794487 |
| FGFR2 | up | 2.71867E-42 | 4.2736E-44 | 16.30627238 | 4.027355114 |
| SYT1 | up | 4.51824E-07 | 6.80789E-08 | 3.550556126 | 1.828045013 |
| NAV3 | up | 1.19959E-16 | 7.01303E-18 | 2.632195174 | 1.396266467 |
| SRBD1 | up | 7.78446E-50 | 9.69936E-52 | 3.760946646 | 1.91109584 |
| PYGM | up | 0.001142561 | 0.00030701 | 4.172919433 | 2.061057066 |
| MAST4 | up | 1.68423E-29 | 4.74605E-31 | 2.451442374 | 1.293630849 |
| MAOB | up | 3.59951E-08 | 4.67419E-09 | 6.471512978 | 2.69410304 |
| HES2 | up | 7.39752E-19 | 3.77635E-20 | 2.233891758 | 1.159559282 |
| CLTCL1 | up | 4.07638E-09 | 4.68266E-10 | 2.33638705 | 1.224279294 |
| ASNS | up | 6.0229E-23 | 2.378E-24 | 7.960193756 | 2.992803547 |
| ST6GALNAC2 | up | 2.77293E-08 | 3.53085E-09 | 2.166166792 | 1.115144333 |
| CAMK2A | up | 2.61932E-05 | 5.1337E-06 | 4.600303944 | 2.201729184 |
| OSBPL3 | up | 3.79098E-23 | 1.48283E-24 | 2.130919562 | 1.091476135 |
| VASH1 | up | 1.49371E-97 | 6.518E-100 | 11.81820174 | 3.562938627 |
| MYO3B | up | 6.29044E-05 | 1.31986E-05 | 5.009470078 | 2.324657997 |
| LIMS2 | up | 0.000852043 | 0.00022263 | 2.57060009 | 1.362105187 |
| EVC | up | 2.69068E-36 | 5.6159E-38 | 4.696887341 | 2.23170499 |
| MOV10L1 | up | 0.00482488 | 0.001500657 | 60.20049558 | 5.911703459 |
| TP63 | up | 7.8363E-285 | 2.8839E-288 | 98.80637934 | 6.626532286 |
| CLCN4 | up | 4.20592E-14 | 3.05588E-15 | 2.479300999 | 1.309933432 |
| PTGS2 | up | 3.41275E-51 | 4.09077E-53 | 4.411795082 | 2.141365782 |
| GLI2 | up | 1.76153E-24 | 6.35302E-26 | 2.491803209 | 1.317190135 |
| SNCB | up | 0.00100764 | 0.000267154 | 6.59856257 | 2.722151782 |
| CA12 | up | 5.61593E-70 | 4.19253E-72 | 18.47695935 | 4.207655455 |
| NUAK1 | up | 1.13682E-75 | 7.47079E-78 | 4.433003074 | 2.148284363 |
| ZNF532 | up | 5.71779E-42 | 9.16842E-44 | 2.529560193 | 1.33888657 |
| SCARF1 | up | 0.005839972 | 0.001858131 | 2.347537165 | 1.231147998 |
| TXK | up | 0.014414788 | 0.005087349 | 2.506667296 | 1.325770523 |
| PLXNA2 | up | 2.55969E-84 | 1.43992E-86 | 3.555508596 | 1.830055944 |
| ACACB | up | 5.18912E-15 | 3.47833E-16 | 2.115343588 | 1.080892015 |
| PAG1 | up | 1.81052E-47 | 2.39867E-49 | 8.7704374 | 3.132648795 |
| IL4R | up | 4.19053E-36 | 8.85649E-38 | 2.07346426 | 1.05204318 |
| SMC1B | up | 0.000382705 | 9.37799E-05 | 5.104543008 | 2.351781807 |
| MAP2 | up | 2.44713E-27 | 7.53914E-29 | 2.306460096 | 1.205680333 |
| HOXA9 | up | 0.003939137 | 0.001195554 | 3.007799768 | 1.588708529 |
| ZCWPW1 | up | 1.36182E-05 | 2.54093E-06 | 3.286555608 | 1.716576395 |
| ITCH | up | 3.03475E-35 | 6.71695E-37 | 2.180806423 | 1.124861716 |
| TP73 | up | 1.13234E-38 | 2.14311E-40 | 3.902921444 | 1.964554425 |
| UBE2D4 | up | 1.79472E-08 | 2.21356E-09 | 2.158155572 | 1.109798866 |
| FKBP7 | up | 0.035792273 | 0.014170154 | 2.377394318 | 1.249381211 |
| OSBPL6 | up | 0.000194531 | 4.5071E-05 | 2.019231651 | 1.01380643 |
| SP140 | up | 5.83239E-05 | 1.21854E-05 | 10.61007598 | 3.407363083 |
| TNS1 | up | 2.97585E-49 | 3.77046E-51 | 3.122292046 | 1.642605487 |
| RAPGEF3 | up | 1.24646E-39 | 2.26736E-41 | 5.528168499 | 2.46680159 |
| DUSP13 | up | 1.78371E-07 | 2.54414E-08 | 2.117620979 | 1.082444393 |
| MID2 | up | 8.69328E-06 | 1.57495E-06 | 2.122384215 | 1.085685851 |
| MOK | up | 3.0271E-193 | 3.6604E-196 | 7.64052189 | 2.933671186 |
| RBL1 | up | 3.57058E-15 | 2.35961E-16 | 2.181086702 | 1.12504712 |
| IGSF9B | up | 0.00596431 | 0.001900514 | 32.51176933 | 5.022890166 |
| TCF7 | up | 0.005235335 | 0.001645108 | 4.347514298 | 2.120190772 |
| ARG2 | up | 0.000402752 | 9.92004E-05 | 2.369852159 | 1.244797061 |
| SMARCD3 | up | 1.76257E-15 | 1.13448E-16 | 2.149451066 | 1.103968267 |
| MPP4 | up | 0.040171469 | 0.016181788 | 4.65483769 | 2.218730863 |
| TRAF5 | up | 7.93242E-26 | 2.63566E-27 | 3.715588214 | 1.893590622 |
| SSH1 | up | 4.1307E-46 | 5.64629E-48 | 2.264087424 | 1.178929666 |
| CD82 | up | 1.06916E-47 | 1.41086E-49 | 3.420413777 | 1.774170863 |
| MECOM | up | 6.21107E-07 | 9.53163E-08 | 2.242668857 | 1.165216614 |
| FAT2 | up | 8.92843E-87 | 4.69399E-89 | 322.0483968 | 8.3311337 |
| LAT2 | up | 3.61593E-13 | 2.86484E-14 | 3.552442259 | 1.8288112 |
| ACHE | up | 0.006108453 | 0.001952868 | 3.143761205 | 1.652491637 |
| KLHL42 | up | 0 | 0 | 8.546606404 | 3.095351684 |
| PTHLH | up | 3.4849E-278 | 1.6489E-281 | 36.37905371 | 5.185036111 |
| ERGIC2 | up | 6.32963E-97 | 2.7953E-99 | 3.248574517 | 1.699806798 |
| DNMT3B | up | 2.14525E-23 | 8.23319E-25 | 2.428035204 | 1.279789339 |
| TMEM40 | up | 3.60053E-37 | 7.13633E-39 | 2.751957055 | 1.460457957 |
| C20orf194 | up | 4.75407E-16 | 2.90928E-17 | 2.100187622 | 1.070518218 |
| EBF4 | up | 4.07362E-15 | 2.70489E-16 | 2.52087689 | 1.333925664 |
| LHX5 | up | 4.14133E-67 | 3.28763E-69 | 6.223210081 | 2.637658949 |
| ANKRD24 | up | 7.584E-05 | 1.6176E-05 | 2.345896555 | 1.230139397 |
| EFNB1 | up | 1.28472E-89 | 6.34897E-92 | 5.340124086 | 2.416873266 |
| DLL3 | up | 0.000993034 | 0.000262863 | 2.034130884 | 1.024412511 |
| PUS7 | up | 1.85704E-15 | 1.19891E-16 | 2.534405161 | 1.341647178 |
| ITGA6 | up | 5.81E-50 | 7.20867E-52 | 2.500940335 | 1.32247064 |
| NLRP1 | up | 4.9683E-98 | 2.1418E-100 | 11.38429103 | 3.508972544 |
| RGS17 | up | 7.3228E-06 | 1.3101E-06 | 2.006858994 | 1.004939254 |
| SLC7A8 | up | 8.71415E-15 | 5.98322E-16 | 3.25756265 | 1.703792925 |
| TGM1 | up | 3.2123E-23 | 1.2531E-24 | 2.49363134 | 1.318248192 |
| PHGDH | up | 5.79968E-32 | 1.49405E-33 | 2.022986249 | 1.016486514 |
| TLL2 | up | 2.33631E-45 | 3.26722E-47 | 29.27613883 | 4.871653387 |
| IL11 | up | 7.90073E-17 | 4.56075E-18 | 2.150267385 | 1.10451607 |
| PCSK5 | up | 2.08075E-31 | 5.51337E-33 | 9.66347317 | 3.272541804 |
| PRTFDC1 | up | 6.1542E-18 | 3.34224E-19 | 2.02330531 | 1.016714034 |
| IGF2-AS | up | 0.000852552 | 0.000222808 | 2.92017333 | 1.546054004 |
| ARVCF | up | 1.82824E-08 | 2.26067E-09 | 2.019088781 | 1.013704348 |
| MMP11 | up | 4.36515E-10 | 4.58294E-11 | 2.293371226 | 1.197469901 |
| CECR2 | up | 0.001190572 | 0.000320849 | 16.2281314 | 4.020424984 |
| SMARCB1 | up | 4.02169E-35 | 8.96481E-37 | 2.137580505 | 1.095978755 |
| P2RX6 | up | 6.31086E-08 | 8.45385E-09 | 3.354670065 | 1.746170883 |
| GGT5 | up | 0.000217777 | 5.1041E-05 | 2.025861634 | 1.018535642 |
| CYTH4 | up | 2.82476E-21 | 1.2534E-22 | 10.87319551 | 3.442704089 |
| MFNG | up | 1.15042E-07 | 1.59672E-08 | 2.36275777 | 1.240471732 |
| TTC28 | up | 4.40323E-08 | 5.77343E-09 | 2.244321888 | 1.166279607 |
| SLC5A4 | up | 1.43408E-07 | 2.01881E-08 | 13.4653393 | 3.751178678 |
| CYP2D6 | up | 0.029538169 | 0.011384486 | 2.1389062 | 1.096873216 |
| XBP1 | up | 2.55183E-34 | 5.94323E-36 | 2.196106322 | 1.134947902 |
| RAB36 | up | 1.09279E-08 | 1.32255E-09 | 2.28190186 | 1.190236746 |
| TIMP3 | up | 1.64548E-51 | 1.94644E-53 | 2.491198564 | 1.316840018 |
| MIOX | up | 0.013496484 | 0.004722811 | 3.696473839 | 1.886149703 |
| ACR | up | 0.027666762 | 0.01055558 | 2.60121412 | 1.37918516 |
| PNPLA3 | up | 9.00006E-08 | 1.23117E-08 | 2.038854134 | 1.027758564 |
| NCF4 | up | 0.000294859 | 7.07965E-05 | 2.344467066 | 1.229260013 |
| IL2RB | up | 0.00459956 | 0.001422597 | 2.784495019 | 1.477415712 |
| MLC1 | up | 0.000330705 | 8.01161E-05 | 14.80938383 | 3.888439711 |
| COCH | up | 8.96713E-06 | 1.6288E-06 | 2.270548618 | 1.183040928 |
| CDKL1 | up | 0.000468071 | 0.000116446 | 2.237863499 | 1.16212204 |
| PLEK2 | up | 1.01844E-70 | 7.33536E-73 | 4.062842001 | 2.022489261 |
| ISM2 | up | 0.01029704 | 0.003496587 | 2.797121243 | 1.48394279 |
| ASB2 | up | 2.95559E-05 | 5.85959E-06 | 157.8702748 | 7.302595744 |
| DHRS2 | up | 1.50297E-52 | 1.72256E-54 | 11.08077621 | 3.469987041 |
| PCK2 | up | 1.64669E-43 | 2.4673E-45 | 2.984815738 | 1.577641872 |
| NFATC4 | up | 2.91859E-40 | 5.10957E-42 | 3.191889815 | 1.67441085 |
| MMP9 | up | 1.00223E-22 | 4.0045E-24 | 19.03647429 | 4.250694399 |
| PYGB | up | 1.73707E-34 | 4.00912E-36 | 2.043646157 | 1.031145426 |
| PROCR | up | 3.11222E-26 | 1.01444E-27 | 2.586218551 | 1.370844197 |
| NINL | up | 9.81439E-17 | 5.7067E-18 | 2.273116924 | 1.184671895 |
| NFATC2 | up | 2.35478E-22 | 9.70584E-24 | 2.027778153 | 1.019899824 |
| BMP7 | up | 0.002486131 | 0.000718352 | 20.23763628 | 4.33896889 |
| NTSR1 | up | 5.82982E-05 | 1.2177E-05 | 16.34093529 | 4.030418655 |
| BIRC7 | up | 9.39994E-09 | 1.12774E-09 | 3.085241795 | 1.62538356 |
| NKAIN4 | up | 0.004508504 | 0.001391353 | 5.594339578 | 2.483967828 |
| ISM1 | up | 1.86515E-09 | 2.07881E-10 | 14.51832612 | 3.859803223 |
| RASSF2 | up | 2.33056E-14 | 1.67125E-15 | 2.323819894 | 1.216498258 |
| FERMT1 | up | 1.7494E-142 | 3.8628E-145 | 5.090571618 | 2.347827665 |
| MYL9 | up | 6.90825E-36 | 1.47819E-37 | 4.928595981 | 2.301176722 |
| JAG1 | up | 3.9306E-183 | 5.5794E-186 | 7.289131539 | 2.865746935 |
| RNF125 | up | 0.000312522 | 7.53003E-05 | 4.044209748 | 2.015857823 |
| RBBP8 | up | 8.30246E-14 | 6.21124E-15 | 2.075788843 | 1.053659695 |
| VSIG1 | up | 1.94125E-10 | 1.9636E-11 | 76.51629342 | 6.257695084 |
| SMARCA1 | up | 1.80873E-68 | 1.41365E-70 | 3.090331585 | 1.627761644 |
| ASB9 | up | 2.70123E-05 | 5.3056E-06 | 2.399315446 | 1.262622846 |
| FGD1 | up | 1.20951E-12 | 1.00914E-13 | 2.146379216 | 1.10190499 |
| RUBCNL | up | 1.56729E-07 | 2.2198E-08 | 9.230123411 | 3.206349937 |
| PLLP | up | 2.71248E-28 | 8.14271E-30 | 3.132905971 | 1.64750147 |
| CCL22 | up | 4.03739E-06 | 6.93028E-07 | 2.150729572 | 1.104826135 |
| HAS3 | up | 1.1808E-195 | 1.3657E-198 | 12.67491207 | 3.663903834 |
| TANGO6 | up | 4.66318E-36 | 9.87992E-38 | 4.359470805 | 2.124153017 |
| NECAB2 | up | 0.046787781 | 0.019273581 | 2.514845417 | 1.330469723 |
| FOXF1 | up | 1.80798E-06 | 2.9409E-07 | 5.368856752 | 2.424614913 |
| SLC7A5 | up | 6.20652E-58 | 6.26493E-60 | 2.358664802 | 1.237970407 |
| MEFV | up | 0.001024153 | 0.000271801 | 2.39476161 | 1.259882048 |
| HMOX2 | up | 2.80348E-21 | 1.24248E-22 | 2.075256633 | 1.053289756 |
| CORO2B | up | 6.8619E-39 | 1.28789E-40 | 6.260688587 | 2.646321342 |
| AP3B2 | up | 5.24378E-06 | 9.17751E-07 | 9.976169802 | 3.318486021 |
| HOMER2 | up | 4.01965E-59 | 3.93068E-61 | 4.682904793 | 2.227403707 |
| SFRP1 | up | 2.10013E-61 | 1.96532E-63 | 2.833052614 | 1.502357395 |
| PLAT | up | 3.62531E-22 | 1.51332E-23 | 2.116690551 | 1.08181037 |
| SPAG1 | up | 8.55905E-06 | 1.54793E-06 | 2.06233294 | 1.044277258 |
| NCALD | up | 0.02068474 | 0.007610105 | 4.412641299 | 2.141642476 |
| MAP4K1 | up | 2.06886E-08 | 2.58321E-09 | 8.789687041 | 3.135811799 |
| LHB | up | 0.031460435 | 0.012214674 | 2.566509586 | 1.359807649 |
| TUBB4A | up | 2.0184E-21 | 8.89238E-23 | 6.261681645 | 2.646550161 |
| KCNA7 | up | 0.021606268 | 0.007999124 | 41.3923872 | 5.37129355 |
| RELB | up | 7.42734E-17 | 4.26796E-18 | 2.240505443 | 1.163824231 |
| SLC17A7 | up | 0.018192066 | 0.006583039 | 2.323995199 | 1.216607088 |
| AMH | up | 0.013689433 | 0.004802565 | 4.879127201 | 2.286623096 |
| IL4I1 | up | 0.031665052 | 0.012315759 | 3.510919641 | 1.811848976 |
| NOVA2 | up | 2.42617E-09 | 2.73854E-10 | 118.6293121 | 6.890316719 |
| IL27RA | up | 1.94173E-17 | 1.078E-18 | 2.427401636 | 1.279412835 |
| OLFM2 | up | 1.41758E-18 | 7.35582E-20 | 2.773092719 | 1.471495854 |
| EBI3 | up | 2.08798E-06 | 3.43478E-07 | 3.383671199 | 1.758589385 |
| FSD1 | up | 0.018442477 | 0.006686259 | 8.484597461 | 3.084846215 |
| MEIS3 | up | 4.81953E-21 | 2.15879E-22 | 2.249727607 | 1.169750333 |
| RASIP1 | up | 9.42416E-05 | 2.04477E-05 | 3.075667104 | 1.620899361 |
| FGF21 | up | 0.000603376 | 0.000153628 | 5.90313938 | 2.561482406 |
| PLEKHA4 | up | 6.26515E-08 | 8.38274E-09 | 2.031052381 | 1.022227448 |
| MAG | up | 0.000314539 | 7.5869E-05 | 2.475154097 | 1.307518346 |
| TMEM59L | up | 6.16935E-19 | 3.13965E-20 | 5.643649335 | 2.49662835 |
| GRIK5 | up | 0.000146707 | 3.30882E-05 | 2.703377495 | 1.434762981 |
| RASA4 | up | 0.000334751 | 8.12194E-05 | 3.680648884 | 1.87996013 |
| CDK6 | up | 2.75559E-51 | 3.27408E-53 | 2.490538543 | 1.316457738 |
| GSDME | up | 8.376E-104 | 3.1705E-106 | 7.866489659 | 2.975719991 |
| CAV1 | up | 1.20592E-75 | 7.98831E-78 | 2.66700795 | 1.415222125 |
| MET | up | 3.79228E-41 | 6.39989E-43 | 2.312177061 | 1.20925188 |
| CPED1 | up | 2.62857E-11 | 2.45293E-12 | 10.26894974 | 3.360216733 |
| GRB10 | up | 2.43137E-70 | 1.76399E-72 | 3.400798414 | 1.765873491 |
| TFR2 | up | 3.98922E-05 | 8.09128E-06 | 2.219659138 | 1.150338146 |
| SERPINE1 | up | 5.92517E-85 | 3.30197E-87 | 2.97358872 | 1.572205121 |
| GLI3 | up | 3.83848E-58 | 3.81407E-60 | 78.32659012 | 6.291430248 |
| AEBP1 | up | 0.003649522 | 0.001098061 | 14.66356379 | 3.87416387 |
| CLIP2 | up | 1.66698E-23 | 6.37135E-25 | 2.122978163 | 1.086089532 |
| LHX2 | up | 9.93393E-06 | 1.81799E-06 | 2.041693249 | 1.029766127 |
| PRUNE2 | up | 0.006284109 | 0.002017615 | 56.42410885 | 5.818239823 |
| C5 | up | 0.000171726 | 3.93002E-05 | 2.127755148 | 1.089332142 |
| ECM2 | up | 0.000364415 | 8.91065E-05 | 2.994188962 | 1.582165272 |
| TLE4 | up | 4.79315E-11 | 4.57115E-12 | 3.256419547 | 1.703286584 |
| LHX6 | up | 6.09489E-18 | 3.30363E-19 | 6.447876901 | 2.688824201 |
| SUSD1 | up | 5.96874E-11 | 5.73621E-12 | 2.226316448 | 1.154658671 |
| LHX3 | up | 0.011211362 | 0.003839483 | 48.8348603 | 5.609839464 |
| DNMBP | up | 1.10668E-26 | 3.55494E-28 | 2.028176475 | 1.020183189 |
| PALD1 | up | 3.47544E-62 | 3.14271E-64 | 3.308792939 | 1.726305012 |
| UNC5B | up | 1.8218E-08 | 2.25175E-09 | 2.944793843 | 1.558166638 |
| ACTA2 | up | 4.78839E-08 | 6.29859E-09 | 3.460681764 | 1.791056281 |
| TWNK | up | 1.48057E-16 | 8.69456E-18 | 2.143888027 | 1.100229558 |
| ARHGAP21 | up | 7.54663E-37 | 1.52353E-38 | 2.094317344 | 1.066480065 |
| GLRX3 | up | 1.99461E-35 | 4.36232E-37 | 2.124236839 | 1.086944626 |
| PPIF | up | 1.09321E-63 | 9.42569E-66 | 2.447907937 | 1.291549301 |
| KRT23 | up | 0.034186393 | 0.013436595 | 2.840695485 | 1.506244187 |
| RUNDC3A | up | 3.82706E-27 | 1.19313E-28 | 8.836969646 | 3.143551729 |
| WNT3 | up | 9.15057E-19 | 4.70494E-20 | 2.545421839 | 1.347904766 |
| P2RX1 | up | 0.034195461 | 0.013441957 | 20.78563479 | 4.377514903 |
| ASIC2 | up | 0.010224996 | 0.003469973 | 3.021606335 | 1.595315714 |
| KRT32 | up | 0.021898257 | 0.008123343 | 2.110504512 | 1.077587913 |
| HLF | up | 0.000236356 | 5.57307E-05 | 21.27334943 | 4.410975295 |
| NFKB1 | up | 3.39122E-29 | 9.77018E-31 | 2.341828815 | 1.22763562 |
| AREG | up | 5.0231E-152 | 9.2428E-155 | 10.53608914 | 3.397267551 |
| INPP4B | up | 1.92251E-16 | 1.1401E-17 | 2.069804823 | 1.049494732 |
| CPE | up | 1.22193E-41 | 1.99147E-43 | 7.1051354 | 2.828862143 |
| AADAT | up | 0.000533039 | 0.000134038 | 2.460584536 | 1.299001082 |
| SLC2A9 | up | 3.34998E-31 | 9.01734E-33 | 69.25625878 | 6.11387255 |
| FAM149A | up | 0.028460318 | 0.010894253 | 41.50739757 | 5.375296575 |
| CRYAB | up | 8.69394E-05 | 1.86896E-05 | 5.711802991 | 2.513946219 |
| VWA5A | up | 1.14388E-11 | 1.03256E-12 | 6.736995758 | 2.752105391 |
| NRXN2 | up | 4.5866E-34 | 1.08751E-35 | 20.58856523 | 4.36377139 |
| GALNT18 | up | 1.72655E-54 | 1.8971E-56 | 21.63370824 | 4.435209074 |
| NECTIN1 | up | 7.4552E-241 | 6.2711E-244 | 9.357449187 | 3.226115309 |
| KIAA1549L | up | 1.04031E-35 | 2.25334E-37 | 2.477727251 | 1.309017384 |
| MDK | up | 3.35286E-41 | 5.62306E-43 | 2.199022883 | 1.136862617 |
| ELMOD1 | up | 1.26084E-18 | 6.52923E-20 | 11.04727048 | 3.465618052 |
| VWF | up | 1.79845E-08 | 2.22005E-09 | 5.085439111 | 2.346372352 |
| P3H3 | up | 2.72549E-14 | 1.96162E-15 | 21.41704949 | 4.420687836 |
| PPFIBP1 | up | 0 | 0 | 15.85434933 | 3.986806764 |
| CAPRIN2 | up | 3.131E-241 | 2.4691E-244 | 7.38992369 | 2.885559467 |
| IL23A | up | 0.000565806 | 0.000143171 | 4.565206148 | 2.190680008 |
| CYP27B1 | up | 3.94791E-15 | 2.61935E-16 | 8.537882251 | 3.093878266 |
| GLI1 | up | 7.03366E-06 | 1.25542E-06 | 5.196240288 | 2.377468146 |
| TRPV4 | up | 5.72564E-06 | 1.00748E-06 | 5.320309453 | 2.411510162 |
| ALDH2 | up | 4.81097E-49 | 6.1209E-51 | 26.15871193 | 4.709219598 |
| VDR | up | 1.50382E-21 | 6.53836E-23 | 2.106331502 | 1.074732511 |
| FZD10 | up | 8.52841E-05 | 1.82979E-05 | 2.479258549 | 1.30990873 |
| GNB3 | up | 0.009578918 | 0.003223524 | 16.71380868 | 4.062968622 |
| FGFR1OP2 | up | 4.8865E-186 | 6.6794E-189 | 5.929789628 | 2.567980923 |
| ADTRP | up | 6.08435E-05 | 1.27247E-05 | 2.0321423 | 1.02300143 |
| UST | up | 5.09249E-39 | 9.47763E-41 | 8.081638772 | 3.014647868 |
| ULBP1 | up | 6.37889E-52 | 7.41147E-54 | 6.602647823 | 2.723044696 |
| MDGA1 | up | 3.42698E-24 | 1.25758E-25 | 3.143219919 | 1.652243216 |
| BACH2 | up | 3.1395E-19 | 1.55977E-20 | 13.29822382 | 3.73316166 |
| PTP4A1 | up | 0.024263512 | 0.009124489 | 4.385493181 | 2.132739093 |
| BVES | up | 1.9771E-10 | 2.00402E-11 | 2.916492048 | 1.544234141 |
| WASF1 | up | 5.09628E-36 | 1.08511E-37 | 5.364340153 | 2.423400722 |
| ADGRG6 | up | 2.18426E-49 | 2.74453E-51 | 2.550591568 | 1.350831895 |
| HBEGF | up | 7.9496E-57 | 8.27518E-59 | 4.822586159 | 2.269807013 |
| SPARC | up | 8.2697E-210 | 8.2606E-213 | 31.31684611 | 4.968867022 |
| POLR3G | up | 9.25618E-11 | 9.09024E-12 | 2.103818213 | 1.073010049 |
| IRX4 | up | 2.97256E-35 | 6.56366E-37 | 4.956421497 | 2.30929888 |
| PDE4D | up | 0.000496466 | 0.000124162 | 2.106589785 | 1.074909406 |
| FGF1 | up | 0.001510881 | 0.000417258 | 2.980002704 | 1.57531364 |
| STC2 | up | 5.9007E-51 | 7.13507E-53 | 2.468999925 | 1.303926793 |
| CLDN16 | up | 5.40924E-06 | 9.48984E-07 | 10.38736213 | 3.376757423 |
| COL7A1 | up | 2.26988E-79 | 1.42009E-81 | 2.8169496 | 1.494133752 |
| HES1 | up | 1.39368E-05 | 2.60771E-06 | 2.402838151 | 1.264739475 |
| GNB4 | up | 4.36015E-16 | 2.65675E-17 | 3.31758276 | 1.730132455 |
| PLXNA1 | up | 1.27765E-57 | 1.30982E-59 | 2.423329408 | 1.276990527 |
| UPK1B | up | 0.033588244 | 0.013164416 | 2.600641778 | 1.378867691 |
| EFCC1 | up | 0.016517088 | 0.005907458 | 4.63487462 | 2.212530312 |
| AADAC | up | 0.00018168 | 4.18645E-05 | 2.534632374 | 1.341776512 |
| ADAM23 | up | 6.44513E-32 | 1.66372E-33 | 7.988424356 | 2.997910973 |
| IL1A | up | 1.0148E-290 | 3.2012E-294 | 134.5894268 | 7.072421268 |
| LOXL3 | up | 1.84504E-05 | 3.52208E-06 | 3.062711786 | 1.614809609 |
| EVA1A | up | 1.20534E-12 | 1.00503E-13 | 2.563032204 | 1.357851606 |
| EFEMP1 | up | 3.51913E-37 | 6.95648E-39 | 2.281741637 | 1.190135444 |
| CHST10 | up | 4.37055E-08 | 5.7237E-09 | 2.856164129 | 1.514078886 |
| PDCL3 | up | 2.62606E-13 | 2.05849E-14 | 2.169344765 | 1.117259353 |
| PLCD4 | up | 7.84424E-08 | 1.06688E-08 | 8.016881898 | 3.003041221 |
| IL1RL1 | up | 9.45297E-10 | 1.02228E-10 | 3.870966614 | 1.952693865 |
| ODC1 | up | 8.91987E-27 | 2.85121E-28 | 2.118573599 | 1.083093248 |
| QPCT | up | 9.46358E-08 | 1.30055E-08 | 3.420148025 | 1.774058767 |
| DLX2 | up | 0.047040632 | 0.019393966 | 2.340365165 | 1.22673365 |
| SLC1A4 | up | 1.59989E-30 | 4.38222E-32 | 6.130683474 | 2.61604792 |
| EPAS1 | up | 2.31942E-58 | 2.29248E-60 | 2.509873094 | 1.32761442 |
| MSH6 | up | 1.56027E-75 | 1.04177E-77 | 2.83220336 | 1.501924858 |
| MARK1 | up | 2.50496E-62 | 2.23881E-64 | 17.24790863 | 4.108349536 |
| ELAPOR1 | up | 0.00111616 | 0.000299329 | 2.900154299 | 1.536129659 |
| RHOU | up | 3.40402E-05 | 6.83094E-06 | 2.112908133 | 1.079230042 |
| C1orf21 | up | 2.91818E-28 | 8.79092E-30 | 53.79177164 | 5.7493136 |
| PLA2G4A | up | 0.001944677 | 0.000549225 | 2.107428742 | 1.07548385 |
| GADD45A | up | 4.69851E-45 | 6.64476E-47 | 2.896878373 | 1.534499114 |
| WLS | up | 2.76795E-31 | 7.42156E-33 | 2.420786889 | 1.275476079 |
| RGS2 | up | 5.32323E-20 | 2.53274E-21 | 7.607261613 | 2.92737722 |
| CFHR3 | up | 0.004704901 | 0.00145963 | 60.16366564 | 5.910820565 |
| KIF21B | up | 2.63207E-17 | 1.46818E-18 | 2.318327525 | 1.2130844 |
| NID1 | up | 5.40393E-27 | 1.7103E-28 | 303.6820749 | 8.246417944 |
| NT5C1A | up | 9.05047E-06 | 1.64442E-06 | 9.24337932 | 3.208420388 |
| HPCAL4 | up | 3.78398E-74 | 2.56629E-76 | 7.366998637 | 2.881076976 |
| MYCL | up | 4.88755E-10 | 5.16481E-11 | 2.510930913 | 1.328222334 |
| MFAP2 | up | 0.001675889 | 0.00046785 | 75.12223219 | 6.231168027 |
| RGS4 | up | 0.029534401 | 0.011381481 | 37.57931756 | 5.231866962 |
| ARTN | up | 2.55441E-23 | 9.87062E-25 | 2.553143205 | 1.35227446 |
| IRF6 | up | 1.03764E-98 | 4.3642E-101 | 4.050047097 | 2.017938685 |
| UTP25 | up | 1.23874E-24 | 4.42198E-26 | 2.173547115 | 1.120051368 |
| POLR1G | up | 6.22066E-16 | 3.85255E-17 | 3.103268647 | 1.633788595 |
| CHRNB4 | up | 2.31589E-05 | 4.5037E-06 | 46.95885722 | 5.553325395 |
| B4GALT6 | up | 5.27826E-09 | 6.14932E-10 | 2.526302498 | 1.337027397 |
| CFAP94 | up | 0.038658859 | 0.015497282 | 2.553615452 | 1.352541286 |
| IRAG2 | up | 0.007504903 | 0.002452183 | 19.76713798 | 4.305032098 |
| UBE3D | up | 0.000137894 | 3.09774E-05 | 2.080135799 | 1.056677716 |
| TNFAIP3 | up | 7.046E-26 | 2.33743E-27 | 2.478633544 | 1.30954499 |
| SGK1 | up | 1.50422E-14 | 1.06207E-15 | 2.316972887 | 1.212241162 |
| ZNF430 | up | 7.24608E-07 | 1.12228E-07 | 7.812128993 | 2.965715771 |
| SPP1 | up | 4.98945E-84 | 2.85921E-86 | 3.631643015 | 1.860622395 |
| KLF12 | up | 5.50776E-14 | 4.04518E-15 | 2.540381736 | 1.345045303 |
| CCND2 | up | 3.94811E-05 | 7.99959E-06 | 150.2812655 | 7.23152136 |
| CYP20A1 | up | 0.000205234 | 4.77712E-05 | 2.455254732 | 1.295872712 |
| PGF | up | 4.02326E-65 | 3.29967E-67 | 6.146113118 | 2.61967432 |
| IFI27L2 | up | 0.006069973 | 0.00193897 | 3.286935134 | 1.716742986 |
| LTBP2 | up | 1.11162E-60 | 1.05195E-62 | 2.469183577 | 1.304034101 |
| PPP4R4 | up | 9.62604E-26 | 3.21357E-27 | 119.0846567 | 6.895843733 |
| ESRRB | up | 9.54103E-15 | 6.59109E-16 | 15.84567875 | 3.986017554 |
| RHOQ | up | 1.4886E-149 | 2.8956E-152 | 5.953092888 | 2.573639405 |
| CRIPT | up | 2.58354E-39 | 4.7539E-41 | 2.954146598 | 1.562741421 |
| OGFRL1 | up | 1.68759E-24 | 6.06863E-26 | 2.148051917 | 1.103028863 |
| PPP1R3C | up | 2.36912E-10 | 2.4238E-11 | 6.687594309 | 2.741487331 |
| MTHFD1L | up | 7.53941E-27 | 2.39805E-28 | 2.036570733 | 1.026141922 |
| ARAP3 | up | 1.90121E-21 | 8.3461E-23 | 2.066660189 | 1.047301193 |
| TNFSF18 | up | 0.008934553 | 0.002983665 | 29.5907065 | 4.887072237 |
| KIAA1217 | up | 7.48793E-65 | 6.18056E-67 | 3.552304809 | 1.828755379 |
| SMAD9 | up | 0.000427163 | 0.000105505 | 4.288498406 | 2.100472584 |
| EGR1 | up | 1.05263E-32 | 2.62868E-34 | 2.592849587 | 1.374538517 |
| SOCS2 | up | 6.84526E-41 | 1.16601E-42 | 3.440894218 | 1.782783541 |
| NPPB | up | 0.001213512 | 0.000327891 | 4.98838304 | 2.318572249 |
| TBX4 | up | 0.000272074 | 6.48252E-05 | 21.30752277 | 4.413290969 |
| TSHZ3 | up | 6.62866E-22 | 2.81581E-23 | 55.39953721 | 5.791802019 |
| CSTA | up | 3.8455E-118 | 1.213E-120 | 33.55582824 | 5.068491462 |
| PLAU | up | 5.6345E-24 | 2.10024E-25 | 5.174897901 | 2.371530399 |
| CHST3 | up | 2.15304E-22 | 8.82904E-24 | 2.162490783 | 1.112693984 |
| EGR2 | up | 2.43842E-07 | 3.54591E-08 | 5.850700824 | 2.548609448 |
| HVCN1 | up | 0.000554282 | 0.00014002 | 10.83292644 | 3.437351125 |
| RASSF8 | up | 1.4254E-100 | 5.6954E-103 | 4.388154143 | 2.133614204 |
| BHLHE41 | up | 3.74667E-30 | 1.04397E-31 | 2.175010546 | 1.121022396 |
| SSPN | up | 4.85792E-35 | 1.088E-36 | 3.812244736 | 1.930640739 |
| ITPR2 | up | 1.2838E-156 | 2.2948E-159 | 5.306295476 | 2.407705013 |
| CCDC91 | up | 9.96719E-88 | 5.1353E-90 | 3.90812936 | 1.966478221 |
| SARDH | up | 9.73451E-05 | 2.11978E-05 | 2.64695281 | 1.404332474 |
| NDUFAF4 | up | 6.28669E-08 | 8.41817E-09 | 2.060272806 | 1.042835381 |
| BATF3 | up | 0.0128069 | 0.004447168 | 3.4862705 | 1.801684512 |
| G0S2 | up | 4.60252E-15 | 3.06819E-16 | 5.506267402 | 2.461074673 |
| RAB38 | up | 1.72189E-27 | 5.27765E-29 | 2.721999273 | 1.444666681 |
| TTPAL | up | 2.52349E-22 | 1.04278E-23 | 2.423563896 | 1.277130119 |
| TOX2 | up | 2.13084E-44 | 3.10311E-46 | 3.129159203 | 1.645775061 |
| GDAP1L1 | up | 0.000124969 | 2.78307E-05 | 17.83156708 | 4.156361591 |
| CDH26 | up | 3.57634E-06 | 6.08811E-07 | 3.987709528 | 1.995560325 |
| HIF3A | up | 0.013103367 | 0.004564582 | 7.544523324 | 2.915429752 |
| EREG | up | 2.36601E-27 | 7.26435E-29 | 2.560384542 | 1.356360504 |
| SSUH2 | up | 1.21755E-12 | 1.01777E-13 | 2.545996983 | 1.348230709 |
| SH3TC1 | up | 5.2705E-20 | 2.50488E-21 | 3.160344085 | 1.660081642 |
| MT2A | up | 6.4923E-104 | 2.4234E-106 | 4.39322711 | 2.135281082 |
| EFNB2 | up | 4.82509E-27 | 1.51442E-28 | 2.04572 | 1.032608695 |
| IL1B | up | 8.6335E-129 | 2.4056E-131 | 106.176637 | 6.730322542 |
| POLR1B | up | 4.07624E-20 | 1.92872E-21 | 2.26418409 | 1.178991261 |
| PSD4 | up | 2.07734E-12 | 1.76597E-13 | 2.127744945 | 1.089325224 |
| FOXA2 | up | 9.45463E-43 | 1.46136E-44 | 9.008657673 | 3.171312155 |
| FLRT3 | up | 8.92436E-06 | 1.62056E-06 | 5.84908918 | 2.548211986 |
| MKKS | up | 1.42291E-14 | 1.00317E-15 | 2.147114675 | 1.102399246 |
| LRRN4 | up | 8.10963E-13 | 6.62124E-14 | 8.607417607 | 3.105580466 |
| ID1 | up | 1.01492E-22 | 4.06053E-24 | 2.577478794 | 1.365960558 |
| PLAGL2 | up | 1.86732E-21 | 8.17769E-23 | 2.013471871 | 1.009685317 |
| TSKS | up | 0.046951746 | 0.019347447 | 7.397166489 | 2.886972747 |
| FLRT1 | up | 3.6645E-05 | 7.39026E-06 | 16.37604523 | 4.033515087 |
| HIVEP3 | up | 1.45099E-17 | 7.97164E-19 | 2.288148316 | 1.19418057 |
| ECHS1 | up | 1.74103E-94 | 7.87174E-97 | 3.396810934 | 1.76418092 |
| GNG11 | up | 3.31797E-13 | 2.62179E-14 | 2.607160494 | 1.382479396 |
| ADM2 | up | 1.06508E-50 | 1.29349E-52 | 4.503003014 | 2.170887443 |
| ASPHD2 | up | 9.48088E-22 | 4.06729E-23 | 8.014472966 | 3.002607652 |
| ATF4 | up | 1.62295E-41 | 2.67917E-43 | 2.126288531 | 1.08833738 |
| CDC42EP1 | up | 1.1195E-26 | 3.60199E-28 | 2.053134698 | 1.03782828 |
| TPST2 | up | 1.30268E-46 | 1.7601E-48 | 3.433035844 | 1.779484921 |
| BAIAP2L2 | up | 7.27577E-21 | 3.28196E-22 | 5.486185131 | 2.455803307 |
| LIF | up | 6.34441E-26 | 2.09468E-27 | 2.122904648 | 1.086039573 |
| KRT17 | up | 4.74585E-58 | 4.74061E-60 | 3.324564358 | 1.733165306 |
| CPA4 | up | 1.22653E-15 | 7.796E-17 | 3.027279392 | 1.59802183 |
| VGF | up | 6.7587E-177 | 1.0305E-179 | 20.24813394 | 4.339717051 |
| PDE11A | up | 0.013773866 | 0.004835807 | 2.566887422 | 1.360020024 |
| ARHGAP22 | up | 3.95199E-52 | 4.57094E-54 | 20.71515257 | 4.372614541 |
| CGNL1 | up | 1.89232E-12 | 1.6067E-13 | 6.587354232 | 2.719699133 |
| TMOD2 | up | 2.60486E-25 | 8.90152E-27 | 3.735285037 | 1.901218338 |
| CHAC1 | up | 4.87694E-70 | 3.61521E-72 | 11.32631911 | 3.501607177 |
| ATP1B2 | up | 0.002236709 | 0.000640286 | 4.489850108 | 2.166667282 |
| KLK14 | up | 0.013059205 | 0.004546451 | 3.055141806 | 1.611239345 |
| AJUBA | up | 3.0323E-31 | 8.14629E-33 | 2.142443928 | 1.099257447 |
| CNN1 | up | 0.024472078 | 0.009214501 | 4.591843341 | 2.199073423 |
| APOE | up | 2.63393E-07 | 3.8413E-08 | 3.152856198 | 1.65665937 |
| APOC1 | up | 0.0179816 | 0.006502153 | 3.298270324 | 1.721709646 |
| MAS1 | up | 4.98609E-58 | 5.0068E-60 | 387.5225704 | 8.598136529 |
| UNC13A | up | 9.07707E-14 | 6.83369E-15 | 8.761229598 | 3.13113336 |
| GDF15 | up | 7.6078E-118 | 2.4398E-120 | 7.21241139 | 2.850481689 |
| IQCN | up | 0.002001635 | 0.000567416 | 3.445238897 | 1.784604025 |
| LSP1 | up | 2.10646E-06 | 3.46628E-07 | 6.073954365 | 2.602636069 |
| TNNT3 | up | 3.64328E-15 | 2.40957E-16 | 464.1890286 | 8.858568614 |
| TNNI2 | up | 4.78397E-26 | 1.56942E-27 | 9.823946231 | 3.296302664 |
| COL5A1 | up | 1.70554E-83 | 9.95297E-86 | 4.068378297 | 2.024453834 |
| TUBGCP2 | up | 2.2557E-62 | 2.00417E-64 | 2.841614051 | 1.50671062 |
| PNPLA7 | up | 0.004617081 | 0.001428744 | 2.040058801 | 1.028610736 |
| GATA5 | up | 1.72086E-05 | 3.2597E-06 | 2.43783355 | 1.285599625 |
| SESN2 | up | 2.23163E-71 | 1.56042E-73 | 4.346352285 | 2.119805114 |
| SLC6A8 | up | 8.43705E-34 | 2.02266E-35 | 2.302777692 | 1.203375141 |
| PNCK | up | 1.30213E-05 | 2.42476E-06 | 3.528841867 | 1.819194782 |
| DUSP9 | up | 1.72609E-89 | 8.6209E-92 | 5.560720106 | 2.475271722 |
| AKAP12 | up | 3.16038E-68 | 2.49228E-70 | 2.690394408 | 1.427817685 |
| SYNE1 | up | 9.94607E-29 | 2.9387E-30 | 10.00903362 | 3.323230782 |
| SH3BGRL | up | 3.2405E-56 | 3.42433E-58 | 29.45066266 | 4.880228191 |
| NFATC1 | up | 2.4291E-28 | 7.25371E-30 | 3.024160066 | 1.596534502 |
| RFTN1 | up | 2.82956E-12 | 2.42478E-13 | 46.58761714 | 5.541874636 |
| LRRC4B | up | 0.004770404 | 0.001482034 | 2.625497124 | 1.392590615 |
| PDLIM4 | up | 3.60082E-32 | 9.21929E-34 | 3.633003857 | 1.861162897 |
| TNS4 | up | 1.12751E-25 | 3.77001E-27 | 2.157751354 | 1.109528627 |
| PPP1R1B | up | 0.035237134 | 0.013914416 | 2.838996108 | 1.505380871 |
| BRME1 | up | 7.15092E-06 | 1.2771E-06 | 2.766623238 | 1.46812619 |
| RTBDN | up | 0.010000852 | 0.003383391 | 2.361320506 | 1.239593874 |
| MATN3 | up | 9.2503E-05 | 2.00267E-05 | 3.522981165 | 1.816796762 |
| FCRLA | up | 0.000233812 | 5.50695E-05 | 29.84619337 | 4.899475034 |
| SERPINF1 | up | 8.93198E-42 | 1.44163E-43 | 23.85543921 | 4.576246343 |
| RIN2 | up | 9.31027E-36 | 2.01174E-37 | 2.522498386 | 1.334853346 |
| NES | up | 1.35479E-38 | 2.57837E-40 | 2.804403135 | 1.487693753 |
| ACY3 | up | 0.001101113 | 0.000294495 | 7.021913647 | 2.811864255 |
| ZBED3 | up | 2.23841E-36 | 4.66016E-38 | 6.13397446 | 2.61682216 |
| KANK4 | up | 1.46622E-19 | 7.14574E-21 | 11.45408273 | 3.517790024 |
| ALOX5AP | up | 0.000229752 | 5.40528E-05 | 5.791580466 | 2.533957099 |
| WASF3 | up | 1.13145E-40 | 1.94513E-42 | 6.03756512 | 2.593966845 |
| CHRM3 | up | 0.000285848 | 6.83776E-05 | 15.14829763 | 3.921083767 |
| DCLK1 | up | 8.2675E-47 | 1.11271E-48 | 7.054280712 | 2.818498986 |
| EPSTI1 | up | 0.042473646 | 0.017225262 | 2.030137538 | 1.02157747 |
| MORC4 | up | 4.85585E-15 | 3.24983E-16 | 2.269735822 | 1.18252439 |
| BEX2 | up | 0.000506687 | 0.000126905 | 2.871538194 | 1.521823751 |
| FAM83F | up | 0.001427294 | 0.000391397 | 7.906650217 | 2.983066603 |
| SEC14L4 | up | 5.75807E-06 | 1.01382E-06 | 2.462153276 | 1.299920576 |
| KRBA1 | up | 0.002745596 | 0.000800396 | 2.577657066 | 1.366060339 |
| DYDC2 | up | 0.00230879 | 0.000662741 | 24.43897718 | 4.611112002 |
| TMTC1 | up | 5.82654E-09 | 6.82484E-10 | 113.3077934 | 6.824103284 |
| KRAS | up | 2.1953E-254 | 1.1541E-257 | 6.945977571 | 2.796177752 |
| IPO8 | up | 7.5259E-134 | 1.9388E-136 | 3.756264487 | 1.90929865 |
| IRAK2 | up | 4.12743E-23 | 1.6166E-24 | 2.553147076 | 1.352276648 |
| CAMK1 | up | 1.48302E-08 | 1.8221E-09 | 4.077406288 | 2.02765172 |
| GSTM1 | up | 2.89495E-64 | 2.40472E-66 | 11.76678936 | 3.556648821 |
| NGF | up | 5.60666E-47 | 7.51642E-49 | 3.458016932 | 1.789944933 |
| FST | up | 1.24439E-05 | 2.31005E-06 | 2.687349642 | 1.426184038 |
| ECHDC3 | up | 5.36288E-05 | 1.11368E-05 | 42.32355445 | 5.403388889 |
| KCTD1 | up | 8.24743E-16 | 5.16846E-17 | 2.116857034 | 1.081923838 |
| SOX5 | up | 0.016073932 | 0.005737974 | 45.02161784 | 5.492545995 |
| LRP4 | up | 1.73098E-40 | 3.01222E-42 | 5.164266116 | 2.368563345 |
| SPOCD1 | up | 4.36281E-09 | 5.03004E-10 | 2.036138786 | 1.025835901 |
| YARS1 | up | 6.20423E-37 | 1.246E-38 | 2.151930234 | 1.105631306 |
| FHOD3 | up | 4.69162E-25 | 1.62792E-26 | 3.342911191 | 1.741105028 |
| SLC43A3 | up | 1.0258E-41 | 1.66643E-43 | 3.620260644 | 1.856093569 |
| DZIP1 | up | 0.002142238 | 0.000610202 | 2.041111595 | 1.029355062 |
| ETS1 | up | 2.47745E-44 | 3.6209E-46 | 2.737964302 | 1.453103637 |
| SLC37A2 | up | 8.50329E-12 | 7.58193E-13 | 2.335423643 | 1.223684277 |
| PSAT1 | up | 3.52799E-56 | 3.74667E-58 | 3.2432034 | 1.697419506 |
| MSI1 | up | 1.0027E-16 | 5.83561E-18 | 12.57773801 | 3.652800585 |
| ANKRD6 | up | 0.007630709 | 0.002498104 | 4.384322953 | 2.132354072 |
| AVIL | up | 2.0228E-05 | 3.88799E-06 | 6.829037163 | 2.771682185 |
| GLS2 | up | 5.54536E-17 | 3.15737E-18 | 3.795594977 | 1.924326052 |
| ITGA7 | up | 0.01079792 | 0.003684838 | 2.278317147 | 1.187968587 |
| AGAP2 | up | 2.29429E-10 | 2.34362E-11 | 7.333425708 | 2.874487291 |
| B4GALNT1 | up | 5.8456E-15 | 3.93989E-16 | 2.039944441 | 1.02852986 |
| SLC26A10 | up | 0.025863926 | 0.009795685 | 6.765934796 | 2.758289274 |
| KCNH3 | up | 4.59304E-12 | 3.99636E-13 | 3.858465194 | 1.948027092 |
| NHSL1 | up | 1.74591E-25 | 5.90201E-27 | 2.961760882 | 1.566455169 |
| REPS1 | up | 8.1486E-16 | 5.09796E-17 | 2.075835779 | 1.053692315 |
| EGR4 | up | 0.012193757 | 0.004218869 | 5.461948538 | 2.449415721 |
| EMX1 | up | 0.002217599 | 0.000634345 | 7.79675452 | 2.962873712 |
| CCDC102A | up | 1.03944E-14 | 7.20249E-16 | 2.239126401 | 1.162935972 |
| NIBAN1 | up | 4.0622E-59 | 3.99365E-61 | 28.86108615 | 4.85105369 |
| EDAR | up | 0.031530344 | 0.012248447 | 6.72045445 | 2.748558794 |
| EPC2 | up | 6.08733E-13 | 4.93169E-14 | 2.178529707 | 1.123354786 |
| ARHGEF4 | up | 2.10746E-20 | 9.77225E-22 | 2.019646777 | 1.014102997 |
| THSD1 | up | 1.19927E-45 | 1.65821E-47 | 46.56364617 | 5.541132128 |
| RCBTB2 | up | 0.00047435 | 0.000118187 | 4.430291258 | 2.147401548 |
| LCP1 | up | 8.80569E-49 | 1.13422E-50 | 13.62339739 | 3.768014621 |
| ZFHX2 | up | 0.008172043 | 0.002694226 | 2.192082161 | 1.132301872 |
| CIB2 | up | 0.004168546 | 0.001273717 | 2.163562486 | 1.113408788 |
| GATA4 | up | 0.000178551 | 4.10778E-05 | 6.795599304 | 2.764600788 |
| IL1RN | up | 0.000996651 | 0.000263926 | 2.414247075 | 1.271573329 |
| ZFP37 | up | 0.04392908 | 0.017907455 | 2.231190671 | 1.157813808 |
| KIF12 | up | 0.002247263 | 0.000643425 | 71.30902015 | 6.156012675 |
| LMX1B | up | 9.51076E-11 | 9.36025E-12 | 2.751160112 | 1.460040104 |
| MYC | up | 1.3029E-117 | 4.247E-120 | 3.83150802 | 1.937912326 |
| DMRT1 | up | 5.97962E-06 | 1.05628E-06 | 55.50571213 | 5.794564343 |
| TUBB2B | up | 1.37764E-14 | 9.69801E-16 | 4.596356443 | 2.200490682 |
| TLR2 | up | 0.044786296 | 0.018321435 | 2.204031502 | 1.140144844 |
| SLCO2B1 | up | 0.011361083 | 0.003894938 | 3.395813192 | 1.763757096 |
| SORL1 | up | 4.26187E-48 | 5.55672E-50 | 3.368599312 | 1.752148833 |
| TRIM29 | up | 4.02948E-50 | 4.95714E-52 | 2.695558097 | 1.430584004 |
| MMP13 | up | 3.6886E-05 | 7.44081E-06 | 3.359285525 | 1.748154424 |
| CASP1 | up | 2.13691E-10 | 2.17522E-11 | 5.115912858 | 2.35499169 |
| PLCB2 | up | 2.28862E-08 | 2.87085E-09 | 5.462374844 | 2.449528319 |
| DUOX1 | up | 0.00107189 | 0.000285935 | 82.60141713 | 6.368094628 |
| SPTBN5 | up | 1.68965E-07 | 2.40376E-08 | 3.360100256 | 1.74850428 |
| SLC44A5 | up | 1.52029E-10 | 1.52021E-11 | 22.83817655 | 4.513375562 |
| CLCA2 | up | 1.87956E-18 | 9.79259E-20 | 54.46496714 | 5.767256655 |
| EMILIN1 | up | 0.033945427 | 0.013329394 | 2.462362359 | 1.300043083 |
| FBXO11 | up | 1.86916E-95 | 8.35278E-98 | 3.469594288 | 1.794766973 |
| TRIM54 | up | 1.01212E-12 | 8.35407E-14 | 2.580234127 | 1.36750198 |
| BTBD16 | up | 0.025012869 | 0.009443111 | 6.757271259 | 2.756440771 |
| GPR87 | up | 2.95141E-35 | 6.48592E-37 | 3.611886297 | 1.852752477 |
| PRKG2 | up | 2.01561E-06 | 3.30937E-07 | 244.2481937 | 7.932204083 |
| FGF5 | up | 1.92564E-42 | 3.00675E-44 | 117.5371613 | 6.87697315 |
| FGF2 | up | 0.000277115 | 6.60991E-05 | 15.59415049 | 3.962933057 |
| LEF1 | up | 3.88641E-12 | 3.36518E-13 | 6.547950259 | 2.711043363 |
| EGF | up | 0.000315598 | 7.61743E-05 | 2.613437115 | 1.385948445 |
| SHISAL1 | up | 2.64673E-34 | 6.17817E-36 | 4.695572519 | 2.231301073 |
| PIK3C2G | up | 0.000542516 | 0.000136677 | 3.329461038 | 1.735288658 |
| SINHCAF | up | 3.1038E-212 | 2.9372E-215 | 5.736024502 | 2.520051187 |
| ETFBKMT | up | 1.32789E-06 | 2.13206E-07 | 3.524542951 | 1.817436187 |
| PIANP | up | 1.7316E-19 | 8.48457E-21 | 32.12586379 | 5.00566334 |
| COL2A1 | up | 0.004612116 | 0.001426965 | 15.62758362 | 3.966022817 |
| INHBE | up | 4.68882E-09 | 5.42317E-10 | 17.18810066 | 4.103338226 |
| GLIPR1 | up | 0.006046794 | 0.001930612 | 2.27452371 | 1.185564474 |
| PHLDA1 | up | 7.2661E-183 | 1.0696E-185 | 5.578262193 | 2.479815746 |
| DUSP6 | up | 2.96158E-91 | 1.41688E-93 | 4.42439665 | 2.14548073 |
| SDSL | up | 2.14001E-13 | 1.66062E-14 | 2.946121379 | 1.55881687 |
| SLC7A1 | up | 2.77325E-33 | 6.80883E-35 | 2.018611674 | 1.013363402 |
| N4BP2L1 | up | 1.99158E-05 | 3.82275E-06 | 2.695914776 | 1.43077489 |
| MAP3K12 | up | 1.56844E-31 | 4.13115E-33 | 3.872357073 | 1.953211991 |
| WDFY2 | up | 5.35477E-26 | 1.76231E-27 | 2.321344567 | 1.214960684 |
| HAPLN3 | up | 9.8806E-05 | 2.15834E-05 | 2.068134024 | 1.048329681 |
| SH3GL3 | up | 1.21994E-08 | 1.48348E-09 | 7.887972765 | 2.979654571 |
| ITGAX | up | 2.46536E-28 | 7.37495E-30 | 8.668289905 | 3.115747404 |
| TGFB1I1 | up | 0.007163805 | 0.002330939 | 2.047799397 | 1.034074396 |
| ARMC5 | up | 9.49784E-08 | 1.30626E-08 | 2.064930113 | 1.046092955 |
| CMTM3 | up | 1.49507E-38 | 2.85322E-40 | 4.659889735 | 2.220295818 |
| RPL3L | up | 0.033195604 | 0.012996565 | 37.69081936 | 5.236141253 |
| TAF4B | up | 1.11996E-18 | 5.78201E-20 | 2.764991819 | 1.467275212 |
| PIK3R5 | up | 0.000126655 | 2.82461E-05 | 12.61630933 | 3.657218033 |
| ELAC1 | up | 0.013894463 | 0.004881799 | 3.236743259 | 1.694542935 |
| IGFBP4 | up | 3.55402E-41 | 5.97911E-43 | 2.719758602 | 1.443478608 |
| DUS3L | up | 3.45305E-16 | 2.08043E-17 | 2.490293578 | 1.31631583 |
| EMP3 | up | 1.15852E-08 | 1.4033E-09 | 2.31629466 | 1.211818793 |
| RCN3 | up | 6.23362E-05 | 1.30598E-05 | 2.22300198 | 1.152509233 |
| CFAP74 | up | 0.009971901 | 0.003372024 | 2.31648621 | 1.211938094 |
| PRDM16 | up | 3.03509E-19 | 1.5063E-20 | 13.03172904 | 3.703956607 |
| PADI3 | up | 1.3982E-188 | 1.8378E-191 | 9.249006231 | 3.209298362 |
| PADI1 | up | 7.4476E-06 | 1.334E-06 | 2.26433491 | 1.179087358 |
| CCN1 | up | 6.34572E-34 | 1.51128E-35 | 2.328492703 | 1.219396361 |
| ZNF697 | up | 5.17548E-35 | 1.16184E-36 | 2.766326776 | 1.467971587 |
| CELSR2 | up | 2.49809E-33 | 6.12014E-35 | 2.171924362 | 1.118973861 |
| XPR1 | up | 1.97447E-33 | 4.79577E-35 | 2.77834366 | 1.474225061 |
| RGS16 | up | 1.59875E-05 | 3.01578E-06 | 8.245328654 | 3.043577 |
| HMCN1 | up | 0.000872631 | 0.000228409 | 2.595655016 | 1.37609865 |
| ECM1 | up | 3.96885E-28 | 1.19769E-29 | 2.968398647 | 1.569684854 |
| LSP1P4 | up | 0.000224453 | 5.27236E-05 | 2.594597879 | 1.375510961 |
| SYT14 | up | 1.66197E-15 | 1.0686E-16 | 12.40989624 | 3.633419148 |
| DYRK3 | up | 1.79429E-06 | 2.91675E-07 | 2.01116886 | 1.008034217 |
| SUSD4 | up | 3.71921E-34 | 8.75982E-36 | 7.383507541 | 2.884306333 |
| ATP8B2 | up | 2.99422E-39 | 5.52532E-41 | 13.30264526 | 3.733641252 |
| AQP10 | up | 0.008370294 | 0.002770149 | 52.57447869 | 5.716290735 |
| KCNN3 | up | 0.000233711 | 5.50335E-05 | 6.306411787 | 2.656819376 |
| CAMKMT | up | 1.50728E-17 | 8.30469E-19 | 3.483704323 | 1.800622181 |
| CALM2 | up | 1.07995E-52 | 1.23206E-54 | 2.18091406 | 1.124932921 |
| MEIS1 | up | 6.11998E-08 | 8.16919E-09 | 216.0712668 | 7.755363425 |
| DQX1 | up | 4.00972E-10 | 4.18026E-11 | 11.33922849 | 3.503250579 |
| THNSL2 | up | 9.32275E-15 | 6.4305E-16 | 2.132609387 | 1.092619743 |
| DLX1 | up | 4.70752E-06 | 8.1672E-07 | 11.27404457 | 3.494933271 |
| ABCA12 | up | 1.38578E-39 | 2.52807E-41 | 3.211621536 | 1.683301893 |
| ACKR3 | up | 3.3562E-99 | 1.3939E-101 | 8.973714636 | 3.165705307 |
| ESPNL | up | 0.001046632 | 0.000278372 | 2.822955902 | 1.497206592 |
| MARCHF4 | up | 1.44845E-17 | 7.95009E-19 | 2.14670187 | 1.102121846 |
| LRIG1 | up | 2.09034E-08 | 2.61224E-09 | 24.14652731 | 4.593743814 |
| NXPE3 | up | 7.14891E-42 | 1.15008E-43 | 3.350204004 | 1.744248948 |
| TRPC1 | up | 1.04981E-08 | 1.26722E-09 | 3.550719174 | 1.828111262 |
| UCN2 | up | 1.60178E-05 | 3.02234E-06 | 7.102853735 | 2.828398777 |
| PLAC8 | up | 5.8914E-15 | 3.97385E-16 | 6.537113273 | 2.708653696 |
| ANK2 | up | 5.06448E-08 | 6.6804E-09 | 12.91897477 | 3.691419679 |
| USP53 | up | 3.36876E-15 | 2.2227E-16 | 2.016119861 | 1.011581412 |
| CYP4V2 | up | 2.77038E-12 | 2.37116E-13 | 3.166072849 | 1.662694451 |
| NKD2 | up | 2.4418E-63 | 2.13101E-65 | 4.96552503 | 2.311946269 |
| IQGAP2 | up | 0.002770167 | 0.000808287 | 6.707407087 | 2.745755165 |
| TENM2 | up | 1.52821E-08 | 1.88004E-09 | 12.77359883 | 3.675093142 |
| DOK3 | up | 0.000655649 | 0.000168109 | 3.586606202 | 1.84261935 |
| SCUBE3 | up | 0.000212433 | 4.9632E-05 | 3.91855456 | 1.970321585 |
| SLC22A3 | up | 4.05855E-13 | 3.23045E-14 | 41.11779441 | 5.361690974 |
| SDK1 | up | 5.66051E-16 | 3.48779E-17 | 3.071371505 | 1.618883027 |
| CREB5 | up | 3.95218E-16 | 2.39986E-17 | 2.444383995 | 1.28947094 |
| KDM6A | up | 2.95049E-21 | 1.31229E-22 | 2.232355544 | 1.158566822 |
| MSN | up | 9.2209E-170 | 1.5028E-172 | 4.892867222 | 2.290680133 |
| SLC16A2 | up | 9.25596E-32 | 2.41363E-33 | 4.127690919 | 2.045334946 |
| RADX | up | 3.97298E-13 | 3.16025E-14 | 8.485782896 | 3.085047769 |
| SYBU | up | 1.22286E-09 | 1.33852E-10 | 2.88843646 | 1.530288759 |
| FBXO10 | up | 0.002705409 | 0.000787827 | 2.347830468 | 1.231328238 |
| STOM | up | 1.4679E-159 | 2.5466E-162 | 6.799939867 | 2.765521988 |
| REXO4 | up | 1.67776E-16 | 9.90549E-18 | 2.062436405 | 1.044349635 |
| PDSS1 | up | 6.29349E-12 | 5.53877E-13 | 2.557842731 | 1.354927563 |
| FAM171A1 | up | 4.03539E-46 | 5.49479E-48 | 20.47028557 | 4.355459324 |
| ANKRD1 | up | 4.31107E-06 | 7.43405E-07 | 2.397932212 | 1.261790876 |
| FUOM | up | 1.39309E-31 | 3.65466E-33 | 4.120586954 | 2.042849856 |
| MTG1 | up | 3.79685E-42 | 6.04829E-44 | 7.769517918 | 2.957825085 |
| PPRC1 | up | 2.52506E-34 | 5.86761E-36 | 2.238129122 | 1.16229327 |
| SYT8 | up | 2.23423E-46 | 3.0305E-48 | 7.254910419 | 2.8589578 |
| SERPING1 | up | 1.05824E-40 | 1.81371E-42 | 7.94494772 | 2.990037726 |
| SLC43A1 | up | 3.88701E-08 | 5.06592E-09 | 3.05011008 | 1.608861311 |
| SESN3 | up | 5.38483E-20 | 2.56488E-21 | 11.67014342 | 3.544750386 |
| KLHL35 | up | 1.79947E-07 | 2.56851E-08 | 2.690048982 | 1.427632443 |
| TENM4 | up | 0.035886428 | 0.014216089 | 3.922269583 | 1.971688697 |
| CAPN5 | up | 1.96697E-15 | 1.27402E-16 | 2.239938171 | 1.16345891 |
| PLCH2 | up | 2.72272E-08 | 3.45977E-09 | 2.391358837 | 1.257830631 |
| FEZ1 | up | 1.253E-11 | 1.13568E-12 | 9.840893597 | 3.298789325 |
| TAGLN | up | 6.50813E-05 | 1.37133E-05 | 2.097528533 | 1.068690436 |
| KIAA1755 | up | 4.28235E-14 | 3.11366E-15 | 21.03354823 | 4.394620339 |
| SOGA1 | up | 1.04384E-30 | 2.83722E-32 | 2.146623914 | 1.102069455 |
| FERMT3 | up | 3.42884E-32 | 8.76092E-34 | 11.71447524 | 3.550220424 |
| HMGA2 | up | 2.9248E-147 | 5.8431E-150 | 4.057925688 | 2.020742445 |
| MPP7 | up | 3.38995E-05 | 6.79559E-06 | 2.480840679 | 1.310829087 |
| ADGRL3 | up | 1.3197E-08 | 1.61242E-09 | 5.563547102 | 2.476004983 |
| FAM124A | up | 0.042332516 | 0.017163575 | 2.721320308 | 1.444306776 |
| PIP4K2A | up | 1.31694E-30 | 3.59336E-32 | 2.460490476 | 1.298945932 |
| ITPR1 | up | 6.53944E-26 | 2.16595E-27 | 3.571962576 | 1.836716965 |
| SLC7A11 | up | 2.02457E-12 | 1.72005E-13 | 3.431515823 | 1.778846007 |
| ENKUR | up | 7.56045E-16 | 4.71808E-17 | 8.217299841 | 3.03866441 |
| BTBD11 | up | 4.60626E-19 | 2.31512E-20 | 2.617963213 | 1.388444825 |
| RAD9B | up | 0.024291688 | 0.009137639 | 3.212313959 | 1.683612903 |
| MAT1A | up | 0.015171291 | 0.005381457 | 5.358312637 | 2.421778759 |
| CCDC3 | up | 6.24864E-05 | 1.31077E-05 | 8.966810343 | 3.164594884 |
| FRMD4A | up | 4.6125E-09 | 5.33247E-10 | 2.125344506 | 1.087696713 |
| **AKR1C2** | **up** | **7.71804E-40** | **1.38366E-41** | **2.057591538** | **1.040956614** |
| ADAM8 | up | 1.32269E-66 | 1.07785E-68 | 3.076224897 | 1.62116098 |
| PIGF | up | 2.956E-35 | 6.51157E-37 | 3.101746183 | 1.633080635 |
| INPP1 | up | 2.06266E-14 | 1.47589E-15 | 3.373392046 | 1.754199994 |
| TMEM267 | up | 9.94252E-11 | 9.81132E-12 | 2.26481067 | 1.179390451 |
| PARP8 | up | 4.58702E-64 | 3.88261E-66 | 108.5998193 | 6.762877893 |
| PSTPIP2 | up | 6.08903E-44 | 8.96341E-46 | 6.775984097 | 2.760430489 |
| PLEKHH2 | up | 2.38211E-23 | 9.17978E-25 | 3.062822266 | 1.614861651 |
| SLC16A12 | up | 2.35226E-06 | 3.89921E-07 | 27.63094669 | 4.788213088 |
| PRDM8 | up | 0.009025397 | 0.003017797 | 4.22187325 | 2.077883266 |
| MED21 | up | 1.28472E-89 | 6.34467E-92 | 3.502948359 | 1.80856972 |
| ADGRA3 | up | 1.3296E-21 | 5.74593E-23 | 2.159277491 | 1.110548657 |
| LGI2 | up | 2.28217E-09 | 2.5676E-10 | 6.290252129 | 2.653117845 |
| MR1 | up | 1.16001E-14 | 8.0867E-16 | 2.198749012 | 1.136682929 |
| BMP6 | up | 2.11059E-15 | 1.37148E-16 | 2.305911442 | 1.205337108 |
| MERTK | up | 4.49503E-35 | 1.00436E-36 | 6.147335173 | 2.619961148 |
| PLEKHG4B | up | 0.01018045 | 0.003452715 | 2.128237021 | 1.089658832 |
| GOLGA8F | up | 0.000173191 | 3.97081E-05 | 116.404675 | 6.863005191 |
| PID1 | up | 3.17539E-05 | 6.33685E-06 | 2.82707558 | 1.499310453 |
| CEBPG | up | 1.295E-36 | 2.65523E-38 | 2.690018798 | 1.427616255 |
| KCTD15 | up | 5.88771E-24 | 2.19772E-25 | 2.292483625 | 1.196911429 |
| ANKFN1 | up | 0.000130158 | 2.91094E-05 | 13.2851555 | 3.73174321 |
| SEMA3D | up | 0.003953991 | 0.00120131 | 2.354757922 | 1.235578753 |
| ANKRD29 | up | 1.19992E-06 | 1.91649E-07 | 2.436410262 | 1.284757086 |
| ROBO3 | up | 1.02154E-05 | 1.8738E-06 | 3.015910891 | 1.592593803 |
| NRGN | up | 0.000658353 | 0.000168906 | 3.013616265 | 1.591495725 |
| LRRK1 | up | 7.63722E-09 | 9.05818E-10 | 2.215793412 | 1.147823379 |
| WNT3A | up | 9.04501E-05 | 1.95347E-05 | 2.804635391 | 1.487813229 |
| OBSCN | up | 1.33724E-41 | 2.18644E-43 | 2.214673339 | 1.147093919 |
| PDLIM3 | up | 7.23014E-08 | 9.76511E-09 | 7.830033489 | 2.969018478 |
| C21orf91 | up | 2.83445E-08 | 3.61963E-09 | 3.111634327 | 1.637672528 |
| WNT7A | up | 2.77679E-43 | 4.20438E-45 | 3.170559104 | 1.664737271 |
| PLCL2 | up | 0.013991456 | 0.004923233 | 3.517811155 | 1.814678037 |
| CYP2U1 | up | 0.000336304 | 8.1614E-05 | 2.217051895 | 1.14864254 |
| TTN | up | 0.049560763 | 0.020607543 | 17.24416155 | 4.108036078 |
| KDM8 | up | 0.000270363 | 6.43464E-05 | 4.199514038 | 2.070222391 |
| SLC26A2 | up | 7.31017E-58 | 7.45583E-60 | 3.409640125 | 1.769619476 |
| RAB39B | up | 0.012390381 | 0.004292761 | 28.21069031 | 4.818170064 |
| GRIP1 | up | 8.31206E-06 | 1.49539E-06 | 2.486472296 | 1.314100357 |
| MMP16 | up | 0.034404388 | 0.01354579 | 6.004626978 | 2.586074625 |
| KCNMA1 | up | 2.96002E-20 | 1.38812E-21 | 11.30890424 | 3.499387243 |
| CFAP161 | up | 0.009294747 | 0.003115189 | 31.19704496 | 4.963337476 |
| EXOG | up | 0.009920091 | 0.003352418 | 2.112510127 | 1.078958257 |
| NMNAT2 | up | 2.37449E-16 | 1.41189E-17 | 2.158085354 | 1.109751925 |
| SYN2 | up | 0.000923018 | 0.000242534 | 4.722320246 | 2.239495882 |
| NRG1 | up | 2.9827E-45 | 4.18685E-47 | 10.49304487 | 3.391361474 |
| ETS2 | up | 9.84619E-32 | 2.57271E-33 | 2.229991043 | 1.157037915 |
| CREB3L1 | up | 3.71745E-14 | 2.69511E-15 | 4.534881778 | 2.181064941 |
| TNFRSF14 | up | 0.000397676 | 9.78247E-05 | 2.008033483 | 1.005783326 |
| SLC30A2 | up | 0.00333627 | 0.000992935 | 5.302462406 | 2.406662487 |
| CFAP251 | up | 2.27508E-20 | 1.05974E-21 | 7.230420914 | 2.854079635 |
| GRHL3 | up | 9.58318E-24 | 3.61743E-25 | 4.47443709 | 2.161706194 |
| HPD | up | 2.26923E-32 | 5.72645E-34 | 7.472813624 | 2.901651541 |
| XDH | up | 7.63198E-37 | 1.54477E-38 | 3.070531065 | 1.618488199 |
| MRAS | up | 2.93814E-06 | 4.9399E-07 | 2.136411587 | 1.095189614 |
| GPR153 | up | 1.23723E-32 | 3.09616E-34 | 2.571063317 | 1.362365139 |
| NRG2 | up | 1.27984E-10 | 1.27372E-11 | 40.33948598 | 5.334120796 |
| FAM86C1P | up | 1.04766E-07 | 1.44583E-08 | 2.053752788 | 1.038262534 |
| PKD1L1 | up | 0.019233834 | 0.00700552 | 4.159372103 | 2.056365756 |
| CACHD1 | up | 4.98431E-27 | 1.56963E-28 | 6.539174339 | 2.709108487 |
| KCNE2 | up | 0.029811491 | 0.011507069 | 37.61601371 | 5.233275065 |
| CIART | up | 5.23926E-16 | 3.22272E-17 | 4.035200812 | 2.012640471 |
| CCDC24 | up | 2.7334E-16 | 1.63248E-17 | 2.187798764 | 1.129480044 |
| HLCS | up | 4.34547E-11 | 4.11678E-12 | 2.03873096 | 1.027671404 |
| PLA2G4D | up | 0.001276603 | 0.000346584 | 2.009404454 | 1.00676798 |
| BTG2 | up | 4.79714E-19 | 2.41358E-20 | 2.655643909 | 1.409061711 |
| STARD9 | up | 1.73725E-10 | 1.74812E-11 | 3.727832102 | 1.898336884 |
| THEM4 | up | 7.6775E-07 | 1.19395E-07 | 2.000997008 | 1.00071901 |
| TEPP | up | 0.013766917 | 0.004831919 | 2.889780161 | 1.530959744 |
| TPPP3 | up | 0.021521792 | 0.007964455 | 2.69633174 | 1.430998008 |
| FAM131B | up | 0.038220287 | 0.01529133 | 6.227632416 | 2.638683792 |
| ABR | up | 2.82712E-60 | 2.71996E-62 | 2.480577643 | 1.310676115 |
| ABCG1 | up | 5.03668E-26 | 1.65497E-27 | 4.622715827 | 2.208740678 |
| CBS | up | 4.5844E-11 | 4.36243E-12 | 2.986892263 | 1.578645201 |
| HSF2BP | up | 0.008563361 | 0.002844849 | 3.326593973 | 1.734045789 |
| RALGDS | up | 0.000194531 | 4.50711E-05 | 2.36256845 | 1.240356129 |
| VAV2 | up | 1.36715E-41 | 2.24253E-43 | 2.383183136 | 1.25288982 |
| ADAMTS13 | up | 0.000168183 | 3.84361E-05 | 2.529247216 | 1.338708057 |
| GPSM1 | up | 1.10391E-22 | 4.42238E-24 | 2.445484565 | 1.290120359 |
| BRSK1 | up | 1.04611E-13 | 7.92516E-15 | 2.14852453 | 1.103346249 |
| PLPP7 | up | 0.017977665 | 0.006499785 | 4.273750614 | 2.095502725 |
| MED27 | up | 5.1623E-16 | 3.17267E-17 | 2.202114529 | 1.138889503 |
| PTH1R | up | 0.038720493 | 0.015524025 | 4.585099387 | 2.196953006 |
| LY6K | up | 8.15126E-36 | 1.75273E-37 | 2.586334428 | 1.370908836 |
| ZNF385A | up | 8.76594E-25 | 3.08312E-26 | 2.664812821 | 1.4140342 |
| SHANK1 | up | 1.09506E-06 | 1.73979E-07 | 58.76297906 | 5.876835632 |
| FMNL3 | up | 1.97562E-33 | 4.80897E-35 | 4.125587599 | 2.044599614 |
| IP6K3 | up | 0.001179127 | 0.000317641 | 5.729849001 | 2.51849712 |
| BCL6B | up | 1.28787E-05 | 2.39617E-06 | 6.427361279 | 2.684226568 |
| TAL1 | up | 0.00030635 | 7.37005E-05 | 33.8555777 | 5.081321632 |
| NOL9 | up | 7.04217E-20 | 3.38391E-21 | 3.080709216 | 1.623262515 |
| SLC45A1 | up | 5.21706E-07 | 7.92665E-08 | 3.338164211 | 1.739054926 |
| RBP7 | up | 9.34614E-05 | 2.02587E-05 | 3.958823296 | 1.985071674 |
| FBLIM1 | up | 4.77315E-20 | 2.26349E-21 | 2.026464864 | 1.018965162 |
| LAPTM5 | up | 1.4854E-175 | 2.3427E-178 | 42.18676055 | 5.398718405 |
| ALPL | up | 2.96143E-05 | 5.87583E-06 | 43.36770806 | 5.438549295 |
| WNT4 | up | 1.11877E-07 | 1.55102E-08 | 2.038810028 | 1.027727354 |
| ATXN7L2 | up | 5.36196E-10 | 5.67458E-11 | 2.749666922 | 1.45925687 |
| GFI1 | up | 0.004591353 | 0.001419817 | 2.592702693 | 1.374456781 |
| NLRP3 | up | 0.000346733 | 8.43272E-05 | 57.27834025 | 5.839917783 |
| OLFML2B | up | 0.02256649 | 0.008405635 | 2.86019768 | 1.516114861 |
| MT2P1 | up | 6.13873E-09 | 7.21957E-10 | 4.520820254 | 2.176584558 |
| IL24 | up | 0.010198877 | 0.0034595 | 64.63556446 | 6.014256293 |
| FCMR | up | 0.00164107 | 0.000457008 | 3.178362409 | 1.668283636 |
| TRIM17 | up | 4.15363E-07 | 6.23231E-08 | 2.483466442 | 1.312355252 |
| DISC1 | up | 1.96511E-10 | 1.99083E-11 | 3.329078187 | 1.735122754 |
| KCNF1 | up | 4.41538E-15 | 2.93879E-16 | 8.798846138 | 3.137314344 |
| ACTG2 | up | 9.47147E-07 | 1.49135E-07 | 9.316656844 | 3.219812356 |
| VSNL1 | up | 2.26002E-07 | 3.27698E-08 | 341.9181269 | 8.417507099 |
| SGCB | up | 1.89858E-15 | 1.22673E-16 | 3.578603487 | 1.839396701 |
| CTSS | up | 9.81828E-07 | 1.54854E-07 | 2.564073772 | 1.358437771 |
| MSX1 | up | 4.80342E-09 | 5.56582E-10 | 13.40366014 | 3.744555106 |
| IVL | up | 0.001028132 | 0.000272911 | 9.041194789 | 3.176513437 |
| ARHGAP25 | up | 0.005071755 | 0.001585973 | 33.92913558 | 5.08445277 |
| NIPAL1 | up | 2.96407E-20 | 1.39157E-21 | 2.457495135 | 1.29718856 |
| ANTXR2 | up | 3.7745E-07 | 5.62772E-08 | 2.270943941 | 1.183292093 |
| COL6A3 | up | 0.039876334 | 0.016050324 | 4.669942432 | 2.223404765 |
| POGLUT1 | up | 2.20823E-05 | 4.27808E-06 | 2.192977069 | 1.132890727 |
| IGFN1 | up | 7.05959E-32 | 1.82604E-33 | 3.547030105 | 1.826611575 |
| TMEM169 | up | 0.01211848 | 0.004185816 | 2.607788716 | 1.382826987 |
| FBLN2 | up | 0.000121868 | 2.71018E-05 | 3.572240818 | 1.836829341 |
| NFASC | up | 1.41717E-06 | 2.2821E-07 | 259.4634253 | 8.019387376 |
| IFI16 | up | 1.7174E-208 | 1.8058E-211 | 39.57647973 | 5.306571387 |
| PRICKLE2 | up | 0.003169152 | 0.000935534 | 4.764541016 | 2.252337241 |
| PTX3 | up | 2.7035E-05 | 5.31148E-06 | 4.827129201 | 2.271165442 |
| CFAP20DC | up | 1.82868E-12 | 1.55074E-13 | 3.609878488 | 1.851950275 |
| APBB2 | up | 1.01154E-31 | 2.64837E-33 | 2.194208878 | 1.13370087 |
| CXCL3 | up | 0.04383525 | 0.017855818 | 3.570846529 | 1.83626613 |
| CXCL1 | up | 0.045355192 | 0.018601328 | 2.202544526 | 1.139171185 |
| PLB1 | up | 2.395E-27 | 7.36593E-29 | 5.639747698 | 2.495630623 |
| TGM4 | up | 0.042568217 | 0.017270329 | 5.752794924 | 2.524263042 |
| NMNAT3 | up | 0.00038277 | 9.38362E-05 | 101.3357845 | 6.66299991 |
| HEYL | up | 1.98715E-18 | 1.03635E-19 | 4.709936817 | 2.235707706 |
| ABLIM2 | up | 1.39452E-12 | 1.17157E-13 | 3.608154743 | 1.851261213 |
| CLDN19 | up | 0.043226879 | 0.017560281 | 19.32574666 | 4.272452249 |
| BSN | up | 5.01053E-06 | 8.72186E-07 | 3.437109364 | 1.781195757 |
| DUSP7 | up | 2.15849E-57 | 2.2242E-59 | 4.759525381 | 2.250817715 |
| PRSS12 | up | 1.6658E-35 | 3.63444E-37 | 6.704473371 | 2.745124014 |
| GASK1B | up | 3.14864E-05 | 6.27212E-06 | 6.763968898 | 2.757870026 |
| ITGA2 | up | 5.1097E-129 | 1.3969E-131 | 3.487175852 | 1.802059119 |
| ADGRV1 | up | 6.21109E-10 | 6.60914E-11 | 9.188720233 | 3.199863943 |
| F2RL2 | up | 4.61033E-48 | 6.03529E-50 | 15.00546786 | 3.907416397 |
| ANKRD33B | up | 6.09619E-40 | 1.08328E-41 | 2.624292397 | 1.391928473 |
| ESM1 | up | 1.36242E-05 | 2.54276E-06 | 180.2715253 | 7.494027724 |
| TERT | up | 2.35661E-12 | 2.01206E-13 | 3.800205894 | 1.926077585 |
| CSF2 | up | 1.11028E-08 | 1.34429E-09 | 14.152675 | 3.823002858 |
| DCBLD1 | up | 9.75778E-10 | 1.05781E-10 | 2.411785241 | 1.270101447 |
| IL31RA | up | 0.000161036 | 3.66686E-05 | 6.44090042 | 2.687262387 |
| STK17A | up | 2.56929E-70 | 1.87756E-72 | 5.211136985 | 2.381598179 |
| ELAPOR2 | up | 7.23097E-35 | 1.63087E-36 | 5.629345925 | 2.492967305 |
| HEY1 | up | 1.12896E-05 | 2.07974E-06 | 2.009404508 | 1.006768019 |
| SHH | up | 8.20813E-15 | 5.61421E-16 | 32.96042384 | 5.042662889 |
| ADCY1 | up | 1.30528E-20 | 5.9702E-22 | 6.370002279 | 2.671293889 |
| EN2 | up | 2.53417E-11 | 2.36217E-12 | 4.411991627 | 2.141430053 |
| CSMD3 | up | 0.012040092 | 0.004153677 | 48.76099326 | 5.607655609 |
| ALDH7A1 | up | 5.57724E-45 | 7.9168E-47 | 2.479120061 | 1.309828141 |
| ABCA1 | up | 8.81929E-22 | 3.7742E-23 | 2.70131979 | 1.43366444 |
| LETM2 | up | 1.64005E-09 | 1.81586E-10 | 2.432860629 | 1.282653675 |
| MAMDC2 | up | 1.00307E-32 | 2.49963E-34 | 9.782704863 | 3.290233417 |
| CPA6 | up | 0.021166813 | 0.007809721 | 41.3555934 | 5.370010564 |
| NOL6 | up | 4.46395E-30 | 1.24618E-31 | 2.820394862 | 1.495897158 |
| AQP3 | up | 3.71026E-42 | 5.89086E-44 | 3.581473078 | 1.840553097 |
| HECTD2 | up | 8.53897E-05 | 1.8334E-05 | 2.075845318 | 1.053698945 |
| INTS6L | up | 0.000244281 | 5.77279E-05 | 109.0352869 | 6.768651297 |
| ZNF22 | up | 6.36561E-27 | 2.02136E-28 | 2.108888502 | 1.076482821 |
| FAAH2 | up | 1.4423E-10 | 1.44147E-11 | 2.438474955 | 1.285979155 |
| TMEM52B | up | 3.69846E-29 | 1.07526E-30 | 2.989031955 | 1.579678321 |
| ENTR1 | up | 7.58146E-15 | 5.17362E-16 | 2.052497752 | 1.037380642 |
| DIPK1B | up | 7.7364E-15 | 5.28341E-16 | 2.131031918 | 1.091552202 |
| STOX1 | up | 0.000869279 | 0.000227454 | 2.011077981 | 1.007969024 |
| NDRG2 | up | 5.73464E-13 | 4.63089E-14 | 2.578517331 | 1.366541743 |
| RNASE7 | up | 5.62011E-12 | 4.9166E-13 | 1521.575742 | 10.57135044 |
| ARHGEF40 | up | 1.6532E-15 | 1.06209E-16 | 2.110415524 | 1.077527082 |
| HSPA12A | up | 7.47943E-19 | 3.82603E-20 | 2.451971182 | 1.293942023 |
| SMCO2 | up | 0.040556705 | 0.016362555 | 2.389870211 | 1.256932271 |
| HTRA1 | up | 6.7781E-102 | 2.6726E-104 | 23.29978257 | 4.542244587 |
| GPR176 | up | 9.44048E-13 | 7.75746E-14 | 2.015657783 | 1.01125072 |
| ADAMTS15 | up | 0.000392684 | 9.64109E-05 | 6.546973261 | 2.710828087 |
| GPT2 | up | 1.41609E-59 | 1.3773E-61 | 3.077177366 | 1.621607602 |
| IKBIP | up | 1.57673E-11 | 1.43822E-12 | 2.671495771 | 1.417647733 |
| CLMP | up | 1.00736E-20 | 4.58143E-22 | 3.418197153 | 1.77323561 |
| APBB1 | up | 1.43965E-13 | 1.09974E-14 | 4.541101941 | 2.183042423 |
| SYNPO2L | up | 1.99845E-08 | 2.4869E-09 | 4.849575745 | 2.277858542 |
| SERPINB8 | up | 1.19939E-11 | 1.08394E-12 | 3.345841944 | 1.742369295 |
| RIMKLB | up | 1.7786E-11 | 1.6345E-12 | 2.003784998 | 1.002727718 |
| A2ML1 | up | 1.85461E-11 | 1.70728E-12 | 25.15887794 | 4.652995676 |
| TK2 | up | 4.14589E-13 | 3.30433E-14 | 2.215490943 | 1.147626429 |
| MMP10 | up | 6.6697E-08 | 8.96961E-09 | 90.28872474 | 6.49647393 |
| BMERB1 | up | 4.01205E-26 | 1.31197E-27 | 12.58674406 | 3.65383323 |
| RBPMS2 | up | 0.000395188 | 9.71894E-05 | 2.146065258 | 1.101693946 |
| NAV2 | up | 2.3775E-111 | 8.2496E-114 | 5.269621576 | 2.397699362 |
| ANKDD1A | up | 0.00015407 | 3.49109E-05 | 2.367164692 | 1.243160083 |
| ZBTB39 | up | 7.71578E-12 | 6.84323E-13 | 2.044525204 | 1.031765848 |
| MTMR10 | up | 1.00824E-10 | 9.95997E-12 | 2.306270928 | 1.205562003 |
| SCG5 | up | 0.004923288 | 0.001534888 | 5.291046986 | 2.403553229 |
| GREM1 | up | 0.001883889 | 0.000530571 | 2.538239536 | 1.343828224 |
| NYAP1 | up | 1.38669E-07 | 1.94652E-08 | 3.363165306 | 1.749819692 |
| SMAD3 | up | 1.56126E-36 | 3.21757E-38 | 2.049540772 | 1.03530069 |
| MARS1 | up | 1.63185E-34 | 3.7577E-36 | 2.077266791 | 1.054686519 |
| B4GALNT2 | up | 6.67421E-05 | 1.41021E-05 | 3.948432578 | 1.981280056 |
| SAMD14 | up | 0.001964067 | 0.00055563 | 2.845970402 | 1.508920658 |
| PIP5KL1 | up | 7.3895E-07 | 1.14644E-07 | 2.121933616 | 1.085379523 |
| KATNAL2 | up | 0.02526441 | 0.009544716 | 6.866719216 | 2.779620972 |
| IGF2 | up | 4.45577E-07 | 6.70908E-08 | 2.606847022 | 1.382305923 |
| CYP2S1 | up | 2.4581E-123 | 7.3663E-126 | 6.117648194 | 2.612977145 |
| AXL | up | 2.9962E-206 | 3.308E-209 | 6.125513924 | 2.61483089 |
| NLRP7 | up | 9.14991E-37 | 1.86645E-38 | 61.2343009 | 5.936268112 |
| NXN | up | 8.61127E-57 | 9.00921E-59 | 2.589205119 | 1.372509261 |
| TLCD3A | up | 1.7083E-24 | 6.15206E-26 | 2.555870373 | 1.353814668 |
| SERPINF2 | up | 2.70665E-06 | 4.5265E-07 | 2.306004312 | 1.205395211 |
| TRPV3 | up | 0.000692906 | 0.000178463 | 3.196598738 | 1.676537657 |
| GGT6 | up | 7.17023E-40 | 1.27791E-41 | 16.14439393 | 4.012961377 |
| KLK13 | up | 2.92167E-07 | 4.28858E-08 | 30.14716841 | 4.913950597 |
| GSDMA | up | 0.00247789 | 0.00071558 | 5.310189618 | 2.408763378 |
| ABCA3 | up | 4.57274E-37 | 9.13538E-39 | 2.316062872 | 1.211674417 |
| ENTPD3 | up | 5.22735E-15 | 3.5067E-16 | 12.27779183 | 3.61797921 |
| SLC22A11 | up | 7.77772E-21 | 3.51246E-22 | 14.27179122 | 3.835094511 |
| CCDC88B | up | 9.36976E-17 | 5.43831E-18 | 2.427055408 | 1.279207044 |
| SCARA5 | up | 0.021959005 | 0.008150993 | 6.245046826 | 2.642712389 |
| KCNJ4 | up | 7.15305E-10 | 7.65659E-11 | 8.147854374 | 3.026420195 |
| DDIT4 | up | 9.8004E-05 | 2.1378E-05 | 2.28690964 | 1.193399363 |
| GNG4 | up | 1.0233E-41 | 1.657E-43 | 2.520778324 | 1.333869254 |
| FOXI1 | up | 0.000559381 | 0.000141455 | 11.9275687 | 3.576228091 |
| KIF5C | up | 2.18905E-11 | 2.02897E-12 | 22.69752812 | 4.504463284 |
| MFSD2A | up | 2.97238E-11 | 2.78782E-12 | 3.58403256 | 1.841583744 |
| BDKRB2 | up | 1.80084E-07 | 2.57141E-08 | 3.387143251 | 1.760069004 |
| PHYHIP | up | 6.53825E-06 | 1.16115E-06 | 4.09219567 | 2.03287513 |
| CAVIN2 | up | 2.61609E-05 | 5.12463E-06 | 161.683216 | 7.337026113 |
| MUCL3 | up | 0.040335153 | 0.016259252 | 5.080256515 | 2.344901344 |
| INPP5D | up | 3.0961E-135 | 7.8131E-138 | 35.93003811 | 5.167118561 |
| AFAP1L2 | up | 3.35566E-47 | 4.46339E-49 | 2.687413902 | 1.426218535 |
| ATF5 | up | 2.67912E-23 | 1.03807E-24 | 2.746384391 | 1.457533563 |
| CPT1C | up | 0.001119112 | 0.000300179 | 2.343929545 | 1.228929205 |
| MN1 | up | 3.88026E-29 | 1.13015E-30 | 6.738641696 | 2.752457817 |
| RAB3B | up | 1.56825E-76 | 1.00587E-78 | 3.521044651 | 1.816003522 |
| SH3TC2 | up | 9.7506E-16 | 6.15147E-17 | 2.003035117 | 1.002187714 |
| ADRB2 | up | 2.7883E-06 | 4.67038E-07 | 2.475114201 | 1.307495092 |
| B3GALNT1 | up | 7.73162E-05 | 1.65111E-05 | 2.089039875 | 1.06284003 |
| CXCL8 | up | 1.10309E-62 | 9.68485E-65 | 5.297598628 | 2.405338542 |
| SPRR1B | up | 0.000318693 | 7.69547E-05 | 6.238930173 | 2.641298663 |
| ZEB2 | up | 0.02662914 | 0.010130301 | 3.596071887 | 1.846421861 |
| BNC1 | up | 9.88636E-14 | 7.47415E-15 | 9.025289334 | 3.173973183 |
| ANTXR1 | up | 5.07593E-18 | 2.72997E-19 | 2.018389368 | 1.013204512 |
| APLF | up | 3.64698E-09 | 4.17789E-10 | 3.549533384 | 1.827629383 |
| BCRP2 | up | 0.012798013 | 0.004443409 | 3.155127141 | 1.657698142 |
| NRG4 | up | 0.00863738 | 0.002872618 | 52.53761001 | 5.715278667 |
| TMEM266 | up | 9.21428E-08 | 1.26242E-08 | 5.024684742 | 2.329033082 |
| P2RY1 | up | 5.46411E-05 | 1.13672E-05 | 6.996873299 | 2.806710367 |
| MUC3A | up | 0.00010227 | 2.2437E-05 | 2.351386913 | 1.233511949 |
| ITGAM | up | 9.4481E-14 | 7.12296E-15 | 6.012601584 | 2.587989364 |
| KLF13 | up | 2.20891E-10 | 2.25408E-11 | 2.191675655 | 1.132034311 |
| ZNF768 | up | 1.1615E-20 | 5.29427E-22 | 2.066126271 | 1.046928427 |
| IFFO2 | up | 1.53405E-25 | 5.15941E-27 | 2.478377396 | 1.309395891 |
| MYO7B | up | 1.29924E-25 | 4.35107E-27 | 31.80313317 | 4.991096998 |
| KCNAB3 | up | 0.000506281 | 0.00012675 | 2.945383324 | 1.558455405 |
| ADRA1B | up | 8.23347E-23 | 3.26811E-24 | 2.842591606 | 1.507206843 |
| FRMPD2 | up | 1.43408E-07 | 2.01907E-08 | 77.1733114 | 6.270030106 |
| OR2A20P | up | 0.047016256 | 0.019380267 | 2.008278805 | 1.00595957 |
| SLC30A1 | up | 1.74478E-21 | 7.61352E-23 | 2.42462463 | 1.277761413 |
| KRT78 | up | 0.000364031 | 8.89935E-05 | 2.70567761 | 1.435989948 |
| KRT86 | up | 8.74142E-06 | 1.58551E-06 | 3.73268468 | 1.900213642 |
| DENND5B | up | 1.5108E-28 | 4.47975E-30 | 2.37986075 | 1.250877161 |
| KRT74 | up | 0.003364705 | 0.001002636 | 36.88270848 | 5.204872699 |
| TMC7 | up | 1.01544E-05 | 1.86154E-06 | 2.059217938 | 1.042096526 |
| SMAGP | up | 1.83272E-17 | 1.01652E-18 | 2.430260689 | 1.281111077 |
| EMB | up | 8.19691E-13 | 6.70112E-14 | 864.457341 | 9.755650962 |
| RASA4B | up | 3.63998E-07 | 5.4214E-08 | 2.488919163 | 1.315519374 |
| HOXB9 | up | 1.8736E-09 | 2.08922E-10 | 4.314006278 | 2.109028276 |
| CEL | up | 1.44845E-17 | 7.9481E-19 | 17.47273877 | 4.127033857 |
| MTSS1 | up | 9.59899E-48 | 1.26163E-49 | 6.667165898 | 2.737073626 |
| CAVIN3 | up | 5.25753E-05 | 1.08932E-05 | 2.709882068 | 1.438230068 |
| PLAC1 | up | 2.84771E-07 | 4.17553E-08 | 3.210959209 | 1.683004338 |
| PKIA | up | 0.021918305 | 0.008133084 | 23.70479751 | 4.567107164 |
| SOX7 | up | 1.42425E-31 | 3.74389E-33 | 5.004593056 | 2.323252762 |
| FAM86JP | up | 0.000637109 | 0.000162953 | 2.059308514 | 1.042159982 |
| NRTN | up | 2.37038E-05 | 4.62011E-06 | 2.564983357 | 1.358949465 |
| PRKCE | up | 1.25078E-27 | 3.82712E-29 | 2.532855007 | 1.340764492 |
| SOCS5 | up | 2.39107E-39 | 4.38716E-41 | 2.455686373 | 1.296126319 |
| NETO2 | up | 8.12022E-33 | 2.01927E-34 | 2.351610581 | 1.233649174 |
| NPTX1 | up | 1.26487E-24 | 4.52189E-26 | 2.165333464 | 1.114589219 |
| APLN | up | 1.38744E-16 | 8.13309E-18 | 3.021492869 | 1.595261537 |
| MCC | up | 2.32078E-43 | 3.48953E-45 | 4.707917873 | 2.235089154 |
| LRRC8C | up | 9.89447E-30 | 2.77259E-31 | 3.763064388 | 1.911907975 |
| LPAR3 | up | 0.031307438 | 0.012153626 | 37.50559169 | 5.229033797 |
| BCL2L1 | up | 9.03415E-40 | 1.6291E-41 | 2.18127267 | 1.125170125 |
| P2RY6 | up | 8.31164E-24 | 3.12872E-25 | 2.27737536 | 1.187372098 |
| PLEKHG5 | up | 1.25947E-40 | 2.17847E-42 | 2.863913243 | 1.51798779 |
| KNDC1 | up | 2.45162E-18 | 1.28504E-19 | 2.685757115 | 1.425328841 |
| COL8A2 | up | 1.35482E-22 | 5.4703E-24 | 26.81604033 | 4.745024319 |
| NINJ2 | up | 1.23827E-10 | 1.2317E-11 | 3.06098246 | 1.613994778 |
| PRNP | up | 2.76037E-91 | 1.3061E-93 | 3.15510082 | 1.657686107 |
| FRMD5 | up | 2.85855E-10 | 2.94707E-11 | 2.507096091 | 1.326017292 |
| CSDC2 | up | 0.017630009 | 0.006360187 | 25.23538576 | 4.657376236 |
| RASGRP1 | up | 6.65302E-08 | 8.94368E-09 | 3.311486241 | 1.727478864 |
| SMPDL3A | up | 0.009378159 | 0.003150048 | 2.141254687 | 1.098456404 |
| BNC2 | up | 0.00363198 | 0.001092019 | 2.809498805 | 1.490312787 |
| ADAMTS20 | up | 1.09404E-05 | 2.01253E-06 | 3.648542562 | 1.867320284 |
| SNCG | up | 6.34551E-17 | 3.62629E-18 | 2.503859184 | 1.324153428 |
| MMRN2 | up | 2.54516E-08 | 3.21674E-09 | 3.406239986 | 1.768180083 |
| ADGRG2 | up | 0.000310824 | 7.48422E-05 | 2.206350427 | 1.141661947 |
| HAP1 | up | 1.00792E-14 | 6.96818E-16 | 2.676976673 | 1.420604568 |
| PHOSPHO1 | up | 0.034095428 | 0.013397257 | 7.019143605 | 2.81129502 |
| C1QTNF1 | up | 1.58745E-23 | 6.04234E-25 | 2.799671231 | 1.485257419 |
| CBY2 | up | 4.99937E-06 | 8.69981E-07 | 24.39720922 | 4.608644223 |
| FAM53A | up | 0.044445947 | 0.018153649 | 2.641803548 | 1.401523188 |
| REP15 | up | 3.69726E-05 | 7.46022E-06 | 13.10436358 | 3.711975385 |
| CHRNA9 | up | 6.08257E-08 | 8.11287E-09 | 8.189216229 | 3.033725381 |
| SNHG11 | up | 1.09869E-06 | 1.74615E-07 | 2.250952459 | 1.170535587 |
| C11orf45 | up | 1.33642E-09 | 1.47054E-10 | 3.725554898 | 1.897455322 |
| CNTNAP2 | up | 1.38185E-06 | 2.22159E-07 | 4.811309187 | 2.266429514 |
| GCSAM | up | 0.029985825 | 0.01158382 | 37.54262118 | 5.230457476 |
| SLCO2A1 | up | 8.22368E-08 | 1.12064E-08 | 2.475246808 | 1.307572384 |
| SH3PXD2B | up | 3.42824E-34 | 8.05649E-36 | 2.22612022 | 1.154531507 |
| RESF1 | up | 2.1139E-127 | 6.0013E-130 | 4.494344372 | 2.168110673 |
| RIN1 | up | 1.29953E-33 | 3.1291E-35 | 2.585967236 | 1.370703997 |
| PDZK1 | up | 4.03504E-05 | 8.19057E-06 | 2.361427545 | 1.23965927 |
| FUT1 | up | 2.25645E-22 | 9.27684E-24 | 3.190728454 | 1.673885834 |
| DES | up | 6.57806E-10 | 7.01692E-11 | 5.41180708 | 2.436110411 |
| PPM1E | up | 1.69327E-16 | 1.0006E-17 | 7.59827033 | 2.925671041 |
| CSRP2 | up | 5.22733E-05 | 1.08224E-05 | 2.092627427 | 1.065315476 |
| DDIT3 | up | 2.90581E-06 | 4.88249E-07 | 2.194793249 | 1.134085043 |
| VWA3A | up | 0.00260182 | 0.000755063 | 8.024071079 | 3.004334386 |
| CATSPER1 | up | 5.17933E-06 | 9.05382E-07 | 2.083763816 | 1.059191765 |
| MCTP1 | up | 2.92138E-12 | 2.50501E-13 | 5.938540141 | 2.57010832 |
| CLCF1 | up | 8.70359E-09 | 1.0387E-09 | 2.302566353 | 1.203242731 |
| FOSL1 | up | 2.77852E-23 | 1.07805E-24 | 5.464015344 | 2.449961535 |
| GPR156 | up | 0.00025914 | 6.13893E-05 | 2.154759428 | 1.107526806 |
| TTLL11 | up | 0.000270646 | 6.44435E-05 | 2.365266641 | 1.24200283 |
| SFN | up | 7.084E-82 | 4.32019E-84 | 2.887479035 | 1.529810472 |
| FAM172BP | up | 3.94406E-06 | 6.75349E-07 | 14.70615579 | 3.878348268 |
| TSPEAR | up | 0.001661238 | 0.000463236 | 75.1588371 | 6.23187084 |
| ARL4D | up | 2.30837E-17 | 1.28398E-18 | 2.726581214 | 1.447093128 |
| DOK7 | up | 1.4666E-23 | 5.56691E-25 | 6.492621912 | 2.698801199 |
| TUBB6 | up | 7.38633E-52 | 8.62081E-54 | 2.611925178 | 1.38511357 |
| NUPR1 | up | 0.010576866 | 0.003599949 | 10.12682374 | 3.340109842 |
| GPX2 | up | 1.476E-136 | 3.5696E-139 | 5.651876809 | 2.49873002 |
| ACBD7 | up | 4.60772E-05 | 9.44025E-06 | 2.154653388 | 1.107455806 |
| KBTBD11 | up | 9.47521E-07 | 1.49244E-07 | 2.135173231 | 1.094353124 |
| RNF152 | up | 4.57189E-07 | 6.8988E-08 | 308.0049414 | 8.266809686 |
| C20orf197 | up | 1.5607E-05 | 2.9399E-06 | 96.66644624 | 6.5949433 |
| ZNF843 | up | 0.036950155 | 0.014707409 | 2.908830028 | 1.540438998 |
| FKBP9P1 | up | 0.000112935 | 2.4943E-05 | 2.615409295 | 1.387036737 |
| GCNT4 | up | 0.000302092 | 7.26285E-05 | 2.078962428 | 1.055863685 |
| SCN4B | up | 0.009129848 | 0.003056085 | 2.113289736 | 1.079490577 |
| PUS1 | up | 7.79178E-12 | 6.91473E-13 | 2.025366365 | 1.018182898 |
| ZBED2 | up | 2.47841E-79 | 1.56358E-81 | 4.782431387 | 2.25774427 |
| TENM3-AS1 | up | 8.23652E-05 | 1.76327E-05 | 3.17971932 | 1.668899422 |
| ZNF620 | up | 0.000494477 | 0.000123535 | 2.545056335 | 1.347697591 |
| ZNF518A | up | 1.25701E-15 | 7.99635E-17 | 2.077955649 | 1.055164863 |
| LRRC75B | up | 9.55586E-13 | 7.87237E-14 | 2.144710852 | 1.100783158 |
| GLDC | up | 3.78751E-34 | 8.96051E-36 | 5.35673667 | 2.421354377 |
| TUBAL3 | up | 0.000173205 | 3.97204E-05 | 8.305273 | 3.054027591 |
| PSAPL1 | up | 1.76604E-07 | 2.51708E-08 | 5.52518036 | 2.46602156 |
| CSRNP3 | up | 5.46141E-05 | 1.13587E-05 | 142.9119314 | 7.158982559 |
| KCTD12 | up | 2.75673E-07 | 4.02908E-08 | 2.463929734 | 1.300961114 |
| THBD | up | 4.88441E-32 | 1.25314E-33 | 9.767105163 | 3.28793103 |
| OPLAH | up | 1.11331E-23 | 4.21421E-25 | 2.618382453 | 1.388675839 |
| LGALS7B | up | 1.61491E-06 | 2.61242E-07 | 139.0560432 | 7.119522633 |
| TRIML2 | up | 1.02215E-40 | 1.74648E-42 | 7.492748937 | 2.905495111 |
| ALOXE3 | up | 9.60089E-11 | 9.45905E-12 | 2.444955515 | 1.289808216 |
| CDH4 | up | 8.82852E-05 | 1.90253E-05 | 12.15442649 | 3.603409916 |
| HLA-DQB1 | up | 9.92723E-05 | 2.17166E-05 | 5.368470516 | 2.424511121 |
| EGR3 | up | 2.87156E-10 | 2.96199E-11 | 4.269773536 | 2.094159553 |
| FJX1 | up | 5.4036E-39 | 1.00851E-40 | 4.309580923 | 2.107547584 |
| ALOX12B | up | 3.20361E-05 | 6.39678E-06 | 3.24056696 | 1.696246245 |
| ARL14 | up | 0.003885092 | 0.001177313 | 2.188071086 | 1.129659609 |
| FOXS1 | up | 0.029811491 | 0.011507069 | 37.61601371 | 5.233275065 |
| AKAP5 | up | 0.03365876 | 0.013199132 | 2.839634879 | 1.50570544 |
| TIGD5 | up | 3.26417E-06 | 5.51894E-07 | 2.036952288 | 1.026412188 |
| PSTK | up | 0.00105402 | 0.000280614 | 2.884128739 | 1.528135564 |
| ZNF816 | up | 0.018785296 | 0.00682141 | 2.511608311 | 1.328611491 |
| HCLS1 | up | 9.06443E-07 | 1.4244E-07 | 22.88147 | 4.516107835 |
| MCFD2 | up | 4.73901E-88 | 2.41672E-90 | 3.470514673 | 1.795149628 |
| SLC47A2 | up | 1.10162E-69 | 8.28202E-72 | 11.71781172 | 3.550631269 |
| SHISA2 | up | 0.001816258 | 0.000509519 | 4.237791728 | 2.083312686 |
| ARSJ | up | 9.24506E-07 | 1.45376E-07 | 7.728715448 | 2.950228651 |
| CXCR2 | up | 0.000680115 | 0.000174811 | 20.29478263 | 4.343036983 |
| OXTR | up | 0.000209406 | 4.88469E-05 | 2.114929251 | 1.080609403 |
| ZNF572 | up | 0.00031459 | 7.58978E-05 | 3.192109593 | 1.674510184 |
| AEN | up | 3.81659E-15 | 2.5262E-16 | 2.461925862 | 1.299787318 |
| FKRP | up | 2.04373E-06 | 3.35983E-07 | 2.038059374 | 1.027196081 |
| RPH3AL | up | 1.0019E-70 | 7.16356E-73 | 4.634149867 | 2.212304701 |
| F2R | up | 1.40867E-34 | 3.22155E-36 | 2.866355133 | 1.519217366 |
| TLCD5 | up | 4.23433E-06 | 7.28837E-07 | 2.82794663 | 1.499754893 |
| DDN | up | 2.99085E-07 | 4.39955E-08 | 4.278904895 | 2.097241613 |
| SOX2 | up | 0.026037922 | 0.009869797 | 10.07416637 | 3.332588556 |
| TMIE | up | 7.22366E-08 | 9.75257E-09 | 6.539897969 | 2.709268128 |
| TNFSF15 | up | 4.66017E-50 | 5.75753E-52 | 5.847416595 | 2.547799379 |
| GPR3 | up | 0.001277212 | 0.000346817 | 3.323075382 | 1.73251902 |
| ODF3L2 | up | 0.014137879 | 0.004979959 | 5.449119593 | 2.446023154 |
| SLC9A9 | up | 0.013103367 | 0.004564088 | 3.696422685 | 1.886129738 |
| OGFRP1 | up | 0.001071158 | 0.000285683 | 3.124577507 | 1.643661127 |
| FAM89A | up | 1.08414E-11 | 9.75785E-13 | 2.73845395 | 1.45336162 |
| TP53TG1 | up | 4.39259E-15 | 2.92131E-16 | 2.243789268 | 1.165937187 |
| EXT1 | up | 4.09916E-51 | 4.93511E-53 | 2.937196754 | 1.554439911 |
| PRR34 | up | 0.005166213 | 0.001618954 | 4.857530284 | 2.28022299 |
| SPACA6 | up | 0.002027676 | 0.000575544 | 2.125275397 | 1.087649801 |
| FAM87A | up | 1.0418E-05 | 1.9137E-06 | 187.9704668 | 7.554362199 |
| CACNB4 | up | 1.31031E-05 | 2.44138E-06 | 12.23236188 | 3.612631087 |
| TEX19 | up | 7.29428E-30 | 2.04014E-31 | 5.355144286 | 2.420925447 |
| FES | up | 8.43406E-06 | 1.52177E-06 | 5.036501439 | 2.332421926 |
| MXRA7 | up | 3.28188E-32 | 8.35092E-34 | 4.45413404 | 2.155144974 |
| LIMK2 | up | 2.33511E-18 | 1.22151E-19 | 2.183529693 | 1.12666215 |
| PLCB1 | up | 0.004578974 | 0.001415507 | 10.98496883 | 3.457458871 |
| LINC01006 | up | 3.40382E-12 | 2.93658E-13 | 2.366092984 | 1.24250677 |
| LCK | up | 1.03198E-61 | 9.38606E-64 | 22.77306821 | 4.509256773 |
| WFDC10B | up | 0.000286629 | 6.86067E-05 | 3.916881343 | 1.969705424 |
| SLC25A21 | up | 0.027797874 | 0.01061291 | 2.912858165 | 1.542435455 |
| NKX2-5 | up | 1.02412E-12 | 8.47467E-14 | 2.672798142 | 1.418350885 |
| RIPPLY3 | up | 0.023360685 | 0.008751813 | 6.127461004 | 2.615289398 |
| C2CD4C | up | 8.61281E-06 | 1.55856E-06 | 2.089749475 | 1.063329999 |
| FHL3 | up | 5.95399E-18 | 3.22413E-19 | 2.453044774 | 1.294573567 |
| TNFAIP8L3 | up | 0.037261106 | 0.014858603 | 7.38267495 | 2.88414364 |
| GOLGA8G | up | 0.000418395 | 0.000103229 | 28.91166132 | 4.853579606 |
| RP1L1 | up | 1.22991E-07 | 1.7135E-08 | 7.013194781 | 2.810071797 |
| RFLNB | up | 3.9403E-06 | 6.74497E-07 | 2.193748611 | 1.133398212 |
| LHFPL6 | up | 7.42955E-19 | 3.7966E-20 | 12.09702602 | 3.596580508 |
| ST6GALNAC3 | up | 9.4913E-05 | 2.06083E-05 | 9.973337892 | 3.318076429 |
| ADAP2 | up | 9.55996E-05 | 2.08026E-05 | 3.38263638 | 1.758148101 |
| KCNJ12 | up | 6.94181E-55 | 7.48157E-57 | 6.374215013 | 2.672247684 |
| GPR173 | up | 1.44231E-30 | 3.94301E-32 | 22.65400403 | 4.50169416 |
| POU6F1 | up | 1.56179E-05 | 2.9435E-06 | 2.307953022 | 1.206613858 |
| MAP7D2 | up | 6.81277E-15 | 4.62399E-16 | 9.896485983 | 3.306916348 |
| NCMAP | up | 0.000118278 | 2.62226E-05 | 5.878553141 | 2.555461116 |
| PTP4A3 | up | 5.51117E-24 | 2.04847E-25 | 2.737818995 | 1.453027069 |
| GAST | up | 1.82115E-07 | 2.60424E-08 | 6.887305189 | 2.783939607 |
| STING1 | up | 6.33583E-24 | 2.36832E-25 | 2.656681897 | 1.409625494 |
| PDE4B | up | 0.000102846 | 2.25741E-05 | 6.651877182 | 2.733761531 |
| TAFA3 | up | 6.62775E-05 | 1.39935E-05 | 4.874852069 | 2.28535844 |
| SNN | up | 3.85236E-45 | 5.42786E-47 | 3.15425986 | 1.65730152 |
| SDR42E1 | up | 3.82217E-06 | 6.53071E-07 | 2.159436462 | 1.110654868 |
| FLRT2 | up | 0.000115877 | 2.56292E-05 | 3.823790447 | 1.935003462 |
| INPP5J | up | 2.02129E-06 | 3.32082E-07 | 2.42511106 | 1.278050818 |
| RBM11 | up | 0.034433061 | 0.013560699 | 2.028911045 | 1.020705613 |
| TMEM105 | up | 2.6922E-06 | 4.49949E-07 | 2.354255956 | 1.23527118 |
| SOCS1 | up | 0.020516142 | 0.007538369 | 2.123351372 | 1.086343128 |
| RAD51D | up | 3.86894E-07 | 5.78684E-08 | 2.388987554 | 1.256399338 |
| INKA1 | up | 6.51085E-05 | 1.37227E-05 | 11.37898279 | 3.50829969 |
| PBX1 | up | 4.79623E-15 | 3.20489E-16 | 2.0248447 | 1.017811262 |
| SHC4 | up | 0.010012616 | 0.003387897 | 27.26531034 | 4.768994672 |
| ZNF696 | up | 5.80183E-15 | 3.90734E-16 | 2.666098051 | 1.414729839 |
| ADARB2 | up | 0.000844944 | 0.00022042 | 86.37776463 | 6.432588076 |
| KCNQ5 | up | 2.1533E-09 | 2.41696E-10 | 22.44442836 | 4.488285448 |
| NAT8L | up | 1.57437E-15 | 1.00732E-16 | 2.744988394 | 1.45680005 |
| THNSL1 | up | 9.38956E-11 | 9.2311E-12 | 2.563991265 | 1.358391347 |
| SDHAP3 | up | 0.00021683 | 5.07735E-05 | 2.405349993 | 1.26624683 |
| MATN1-AS1 | up | 0.000168461 | 3.85084E-05 | 3.390614715 | 1.761546856 |
| KRT5 | up | 7.12534E-91 | 3.44635E-93 | 9.184786223 | 3.199246143 |
| POLR3C | up | 1.76052E-11 | 1.61326E-12 | 2.07162913 | 1.05076575 |
| EDARADD | up | 0.006132752 | 0.001963216 | 8.035461003 | 3.006380795 |
| GPAT2 | up | 1.89188E-21 | 8.2952E-23 | 2.741399772 | 1.454912728 |
| KRT3 | up | 0.021461048 | 0.007937462 | 2.37842481 | 1.250006417 |
| GNG2 | up | 0.001094864 | 0.000292524 | 13.98096298 | 3.805391829 |
| ANKRD20A5P | up | 0.04988579 | 0.020763672 | 2.384259118 | 1.253541035 |
| FOXD2 | up | 7.39452E-05 | 1.57563E-05 | 2.742525671 | 1.455505125 |
| PDE2A | up | 1.5653E-243 | 1.0698E-246 | 30.82644428 | 4.946096584 |
| KRT14 | up | 0 | 0 | 97.7608035 | 6.611184238 |
| TRABD2A | up | 0.00045114 | 0.000111902 | 7.812882475 | 2.965854913 |
| C1QL4 | up | 9.45561E-05 | 2.05229E-05 | 3.218336844 | 1.686315332 |
| FAM183A | up | 7.87051E-06 | 1.41513E-06 | 7.09357686 | 2.826513274 |
| EMID1 | up | 4.97566E-27 | 1.5643E-28 | 5.793348171 | 2.534397371 |
| CCK | up | 0.040827844 | 0.016482678 | 2.975177851 | 1.572975913 |
| MT1X | up | 2.29022E-08 | 2.87512E-09 | 6.3757773 | 2.672601238 |
| GCNT1 | up | 1.02905E-18 | 5.30185E-20 | 4.042965177 | 2.015413777 |
| PLEKHN1 | up | 6.28497E-08 | 8.41257E-09 | 2.345101387 | 1.229650297 |
| SPRY4 | up | 1.82388E-44 | 2.64649E-46 | 3.373866077 | 1.754402708 |
| GABRD | up | 0.007899622 | 0.002596106 | 19.7057927 | 4.300547881 |
| DNAH17 | up | 1.21704E-06 | 1.94639E-07 | 3.124451172 | 1.643602794 |
| DNER | up | 0.000282745 | 6.7561E-05 | 3.146046875 | 1.653540167 |
| RINL | up | 0.001347645 | 0.000367501 | 2.313539008 | 1.210101424 |
| C17orf99 | up | 0.00015548 | 3.52631E-05 | 66.04835604 | 6.045450748 |
| TPRG1 | up | 2.46401E-32 | 6.23095E-34 | 17.71289285 | 4.146727945 |
| TMEM221 | up | 0.004488029 | 0.001384798 | 2.526397203 | 1.337081479 |
| NHS | up | 1.61966E-23 | 6.17347E-25 | 2.434734099 | 1.283764222 |
| LINC00265 | up | 8.47612E-06 | 1.52981E-06 | 2.272705106 | 1.1844105 |
| NCR3LG1 | up | 2.13001E-08 | 2.66741E-09 | 2.094996404 | 1.066947767 |
| SBK1 | up | 2.52886E-18 | 1.32818E-19 | 2.198827857 | 1.136734662 |
| SLC38A3 | up | 0.023244853 | 0.008701086 | 3.438835825 | 1.781920241 |
| CIBAR1 | up | 0.025815484 | 0.009775981 | 2.01932979 | 1.013876546 |
| SELL | up | 0.04008419 | 0.016144524 | 4.657875204 | 2.219671987 |
| SRP72P2 | up | 0.006454136 | 0.002075937 | 32.57636263 | 5.025753621 |
| INSC | up | 0.011390889 | 0.003909948 | 48.90824386 | 5.612005758 |
| PAQR9 | up | 0.000218029 | 5.11115E-05 | 6.582916921 | 2.718726991 |
| PARVB | up | 2.91421E-25 | 1.00046E-26 | 2.678087808 | 1.421203264 |
| FSIP2 | up | 0.006284109 | 0.002017615 | 56.42410885 | 5.818239823 |
| SPRED3 | up | 5.82122E-08 | 7.73674E-09 | 2.716743092 | 1.441878144 |
| KLRG2 | up | 4.52017E-10 | 4.75995E-11 | 3.019347952 | 1.594237024 |
| LRRK2 | up | 0.026834544 | 0.010215495 | 22.25602627 | 4.476124123 |
| SBSN | up | 5.26955E-09 | 6.13641E-10 | 15.47486279 | 3.951854713 |
| ANKDD1B | up | 0.005867385 | 0.00186747 | 15.07577512 | 3.914160275 |
| ALKBH2 | up | 6.2918E-21 | 2.82818E-22 | 2.832545927 | 1.502099348 |
| RELN | up | 0.000391316 | 9.60134E-05 | 2.511763335 | 1.328700536 |
| FAM25G | up | 2.68405E-08 | 3.40639E-09 | 15.65750572 | 3.968782501 |
| MAOA | up | 1.03171E-60 | 9.70902E-63 | 8.440589886 | 3.077343828 |
| LINC01164 | up | 6.41073E-58 | 6.50476E-60 | 383.4550024 | 8.58291348 |
| GJB5 | up | 5.90578E-23 | 2.32244E-24 | 3.437551364 | 1.781381271 |
| SLC35E2B | up | 8.24881E-21 | 3.72955E-22 | 2.124920864 | 1.087409114 |
| NEMP2 | up | 2.65E-05 | 5.19941E-06 | 2.184999364 | 1.127632859 |
| ALG1L | up | 3.51629E-05 | 7.07289E-06 | 6.488120315 | 2.697800573 |
| SPATA41 | up | 0.043961107 | 0.017925574 | 3.273793448 | 1.710963301 |
| RASSF10 | up | 5.23723E-34 | 1.24453E-35 | 25.02449484 | 4.645269041 |
| GJB4 | up | 6.97661E-05 | 1.48108E-05 | 2.037176924 | 1.02657128 |
| PAX5 | up | 1.70303E-07 | 2.42384E-08 | 356.9498905 | 8.479577749 |
| SPOCK3 | up | 0.008370294 | 0.002770149 | 52.57447869 | 5.716290735 |
| PLEKHG4 | up | 3.561E-29 | 1.02968E-30 | 2.742854523 | 1.455678106 |
| FAT4 | up | 0.044296705 | 0.018081048 | 2.855636075 | 1.513812133 |
| CTSE | up | 0.039074651 | 0.015688613 | 2.173057611 | 1.119726423 |
| SEMA4A | up | 3.25317E-15 | 2.14301E-16 | 2.171033799 | 1.118382187 |
| HRCT1 | up | 0.025934618 | 0.009825186 | 2.50366223 | 1.324039941 |
| RYR1 | up | 1.46762E-08 | 1.80163E-09 | 3.593355635 | 1.845331726 |
| FAM217B | up | 5.9994E-10 | 6.37443E-11 | 2.008367647 | 1.00602339 |
| CGB7 | up | 0.000194092 | 4.49491E-05 | 3.465361162 | 1.793005719 |
| NTNG2 | up | 5.29858E-25 | 1.8441E-26 | 108.0313293 | 6.755305947 |
| CRYBA4 | up | 0.029811491 | 0.011507069 | 37.61601371 | 5.233275065 |
| MME | up | 1.24273E-18 | 6.42894E-20 | 13.65497425 | 3.771354689 |
| CACNA1H | up | 1.39553E-61 | 1.29861E-63 | 6.448531968 | 2.688970763 |
| MMP1 | up | 3.26302E-23 | 1.27461E-24 | 159.462083 | 7.31706961 |
| HRH1 | up | 1.76125E-10 | 1.77504E-11 | 2.351628728 | 1.233660307 |
| LINC00173 | up | 0.000877745 | 0.000229854 | 2.001515073 | 1.00109248 |
| ZNF431 | up | 1.65868E-06 | 2.6902E-07 | 5.417296604 | 2.437573082 |
| COL27A1 | up | 1.6267E-115 | 5.5589E-118 | 27.80138009 | 4.797084597 |
| S100A2 | up | 7.65397E-83 | 4.54707E-85 | 14.6686785 | 3.874666999 |
| SNHG17 | up | 2.21605E-13 | 1.72195E-14 | 2.45447019 | 1.295411645 |
| ADA | up | 1.09444E-21 | 4.71241E-23 | 2.136471854 | 1.095230311 |
| GAL3ST4 | up | 0.000413072 | 0.000101851 | 2.801383506 | 1.4861395 |
| SRC | up | 1.62676E-78 | 1.03484E-80 | 3.101384708 | 1.632912495 |
| ACSL5 | up | 1.46389E-12 | 1.2337E-13 | 2.306526113 | 1.205721626 |
| SERPINA1 | up | 0.000391766 | 9.61445E-05 | 54.73201676 | 5.774313113 |
| ZNF655 | up | 3.34278E-51 | 3.98934E-53 | 3.126487535 | 1.644542765 |
| ZNF786 | up | 1.65497E-06 | 2.68331E-07 | 2.309995212 | 1.207889862 |
| MYO5A | up | 1.59837E-33 | 3.85705E-35 | 2.555649065 | 1.353689743 |
| SSPOP | up | 0.000140667 | 3.16447E-05 | 3.334032914 | 1.737268347 |
| COL4A6 | up | 7.95888E-38 | 1.56073E-39 | 2.062820549 | 1.044618322 |
| SERPINB2 | up | 0.004805873 | 0.00149424 | 15.65158533 | 3.968236888 |
| HOXC6 | up | 0.03847368 | 0.01540889 | 2.07366637 | 1.052183799 |
| PRB3 | up | 0.000213129 | 4.98396E-05 | 11.17600854 | 3.482333124 |
| ZNF71 | up | 4.7519E-10 | 5.01396E-11 | 4.195960154 | 2.069000978 |
| DNM3 | up | 0.000475149 | 0.000118431 | 3.746424311 | 1.905514305 |
| ENTPD7 | up | 7.38304E-13 | 6.01248E-14 | 2.232536457 | 1.158683735 |
| NPIPB13 | up | 0.003430789 | 0.001025215 | 2.380301413 | 1.251144271 |
| TMEM229B | up | 5.56939E-10 | 5.89996E-11 | 5.416891424 | 2.437465174 |
| ZXDA | up | 1.95874E-07 | 2.81439E-08 | 2.392466837 | 1.258498927 |
| CSF2RA | up | 7.22577E-11 | 7.02025E-12 | 2.405230497 | 1.266175156 |
| ZNF485 | up | 6.96491E-05 | 1.47823E-05 | 2.460169643 | 1.298757801 |
| HOXC4 | up | 0.035822028 | 0.014186811 | 2.400820076 | 1.263527288 |
| NTRK1 | up | 0.000765249 | 0.000198021 | 10.93989279 | 3.451526694 |
| MT1F | up | 1.17931E-17 | 6.44186E-19 | 2.418157441 | 1.273908178 |
| TPM2 | up | 2.88981E-10 | 2.98385E-11 | 2.183103595 | 1.126380592 |
| ZNF511 | up | 3.55352E-17 | 2.00272E-18 | 3.71091374 | 1.891774466 |
| ARC | up | 0.000582895 | 0.000147953 | 4.630149461 | 2.211058764 |
| CCDC69 | up | 5.37926E-36 | 1.14819E-37 | 5.641655734 | 2.496118632 |
| DLL1 | up | 1.5263E-35 | 3.31404E-37 | 100.4692818 | 6.650610659 |
| SMOC1 | up | 0.030958985 | 0.011993941 | 37.65304415 | 5.234694602 |
| APCDD1L | up | 1.34892E-80 | 8.36828E-83 | 5.553157571 | 2.473308333 |
| RASSF9 | up | 0.018830294 | 0.0068427 | 5.013953684 | 2.325948669 |
| FAM169A | up | 4.33305E-08 | 5.67003E-09 | 2.129897388 | 1.090783927 |
| MUC2 | up | 3.06351E-08 | 3.93791E-09 | 35.52527704 | 5.150773995 |
| ALPK2 | up | 0.008329695 | 0.002752771 | 2.839012848 | 1.505389378 |
| RYR3 | up | 3.78654E-07 | 5.65364E-08 | 10.95156958 | 3.453065747 |
| GRK5 | up | 2.34826E-22 | 9.6666E-24 | 3.1043999 | 1.634314414 |
| DMD | up | 3.96873E-22 | 1.66294E-23 | 6.119955607 | 2.613521188 |
| TGM2 | up | 1.62661E-68 | 1.25709E-70 | 2.564359933 | 1.358598772 |
| LINC00862 | up | 6.80435E-07 | 1.05101E-07 | 2.595512995 | 1.376019711 |
| SAMD5 | up | 7.91012E-39 | 1.49295E-40 | 119.9272885 | 6.90601616 |
| BVES-AS1 | up | 0.036437431 | 0.014465014 | 3.517985932 | 1.814749714 |
| CHIC1 | up | 3.74822E-10 | 3.90173E-11 | 2.504116504 | 1.324301685 |
| NHSL2 | up | 0.007271502 | 0.002368657 | 31.02904783 | 4.955547521 |
| SPIN3 | up | 1.77263E-11 | 1.62622E-12 | 2.392647114 | 1.258607633 |
| NBDY | up | 8.26016E-36 | 1.78049E-37 | 3.330308916 | 1.735656007 |
| LST1 | up | 0.030535881 | 0.011812366 | 2.29825838 | 1.200541001 |
| CDSN | up | 0.042294389 | 0.017143669 | 2.084144033 | 1.059454984 |
| TRIM15 | up | 0.033623173 | 0.013179874 | 4.710888174 | 2.235999086 |
| INSYN2B | up | 0.001438675 | 0.000394821 | 78.78776987 | 6.299899794 |
| LINC01291 | up | 1.07299E-15 | 6.7975E-17 | 10.01712173 | 3.324396127 |
| ZBTB48 | up | 8.87608E-08 | 1.21328E-08 | 2.021577248 | 1.015481333 |
| ZNF783 | up | 6.66569E-13 | 5.41428E-14 | 2.395629313 | 1.26040469 |
| ZNF425 | up | 1.13497E-14 | 7.90618E-16 | 5.17233306 | 2.370815177 |
| PRSS1 | up | 0.019411605 | 0.007080475 | 25.28937437 | 4.660459442 |
| CCDC144NL | up | 2.28018E-06 | 3.76893E-07 | 20.9959262 | 4.392037526 |
| VIT | up | 6.54452E-05 | 1.38074E-05 | 11.51482152 | 3.525420144 |
| MUC12 | up | 0.000280612 | 6.69774E-05 | 4.606242976 | 2.203590514 |
| KRT6A | up | 1.81242E-15 | 1.1682E-16 | 4.387678512 | 2.133457822 |
| KRT81 | up | 1.012E-68 | 7.76784E-71 | 17.91680537 | 4.163241518 |
| CALML3-AS1 | up | 3.5362E-138 | 8.1801E-141 | 20.55218884 | 4.361220146 |
| TECRL | up | 0.010355347 | 0.003518564 | 13.48054242 | 3.752806643 |
| DPF3 | up | 3.19395E-16 | 1.91929E-17 | 7.439400912 | 2.895186447 |
| MANSC4 | up | 1.38455E-09 | 1.52497E-10 | 5.059226089 | 2.338916712 |
| ETFRF1 | up | 2.00855E-34 | 4.64624E-36 | 4.531023441 | 2.179836955 |
| PKD1P4 | up | 5.67574E-08 | 7.52846E-09 | 2.354117101 | 1.235186086 |
| C16orf96 | up | 0.036065094 | 0.014292555 | 20.85768417 | 4.382507079 |
| SERPINB5 | up | 1.34091E-43 | 2.0021E-45 | 2.923811952 | 1.547850526 |
| ANKUB1 | up | 0.001313233 | 0.000357564 | 78.8984965 | 6.301925903 |
| STK38L | up | 1.0909E-145 | 2.3515E-148 | 5.518964876 | 2.464397704 |
| LINC02693 | up | 2.07174E-41 | 3.44183E-43 | 4.807780185 | 2.265370937 |
| ZNF254 | up | 7.87828E-05 | 1.68368E-05 | 5.332939449 | 2.414930948 |
| KLHL23 | up | 2.43423E-10 | 2.49425E-11 | 2.216255927 | 1.14812449 |
| MLLT11 | up | 1.71555E-30 | 4.70805E-32 | 3.423225106 | 1.775356165 |
| PCNPP3 | up | 0.000543318 | 0.000136908 | 30.65685817 | 4.938137947 |
| LCAT | up | 0.00012542 | 2.79443E-05 | 2.252696731 | 1.171653104 |
| HNRNPA1P33 | up | 1.4624E-07 | 2.06279E-08 | 2.414396566 | 1.271662659 |
| FIRRE | up | 1.07298E-08 | 1.29574E-09 | 3.959157955 | 1.985193626 |
| LBH | up | 3.6821E-146 | 7.7432E-149 | 25.95245592 | 4.697799164 |
| GPSM3 | up | 3.03934E-29 | 8.72445E-31 | 4.473093263 | 2.161272838 |
| RPS16P2 | up | 0.006327865 | 0.002032662 | 56.31405658 | 5.815423174 |
| ZNF888 | up | 0.026971124 | 0.010271743 | 2.492781894 | 1.31775666 |
| PRCD | up | 0.03201244 | 0.01246097 | 6.637348657 | 2.73060706 |
| SCART1 | up | 1.11731E-11 | 1.00741E-12 | 3.500874424 | 1.807715313 |
| KRT16P3 | up | 2.01285E-05 | 3.86675E-06 | 4.900958486 | 2.293063926 |
| CXorf49 | up | 0.028908389 | 0.011087091 | 10.13006602 | 3.340571672 |
| OR7E122P | up | 3.04953E-07 | 4.4939E-08 | 8.059251285 | 3.010645817 |
| MUC5AC | up | 0.000578815 | 0.000146735 | 18.14774933 | 4.181718732 |
| MIR99AHG | up | 0.002794992 | 0.000816265 | 6.835819515 | 2.773114306 |
| MIR17HG | up | 0.00110787 | 0.000296698 | 3.673874394 | 1.877302303 |
| TNFRSF25 | up | 5.12139E-24 | 1.89282E-25 | 3.117608979 | 1.640439992 |
| LINC01356 | up | 4.87818E-05 | 1.00559E-05 | 2.737092567 | 1.452644227 |
| TTC34 | up | 0.003579359 | 0.001074316 | 63.90328506 | 5.997818192 |
| ATAD3C | up | 0.001410639 | 0.000386237 | 3.588991448 | 1.843578485 |
| RAET1K | up | 0.001464552 | 0.000402615 | 2.724870989 | 1.446187926 |
| MTMR9LP | up | 0.023003033 | 0.008597264 | 2.070056263 | 1.04966998 |
| PLXNA4 | up | 1.75206E-29 | 4.94642E-31 | 36.56811199 | 5.192514237 |
| NPTXR | up | 1.85914E-22 | 7.59452E-24 | 2.303341439 | 1.203728287 |
| LINC01118 | up | 0.016591241 | 0.005937468 | 2.526579298 | 1.33718546 |
| SMPD4P1 | up | 0.000505293 | 0.000126476 | 5.896676848 | 2.559902133 |
| CKMT1A | up | 1.91063E-05 | 3.65533E-06 | 44.94807812 | 5.490187525 |
| C1GALT1C1L | up | 0.000768261 | 0.000198881 | 3.458293048 | 1.790060125 |
| CHROMR | up | 0.000126004 | 2.80812E-05 | 2.037461828 | 1.02677303 |
| LINC02561 | up | 0.01583027 | 0.005639341 | 2.429833637 | 1.280857541 |
| HAGLR | up | 0.031307438 | 0.012153626 | 37.50559169 | 5.229033797 |
| PINCR | up | 0.00363222 | 0.001092282 | 63.93998136 | 5.998646418 |
| LINC00365 | up | 3.79172E-05 | 7.65681E-06 | 5.432145499 | 2.441522122 |
| LINC01117 | up | 0.00208332 | 0.000592434 | 12.82123702 | 3.680463557 |
| RPL29P19 | up | 0.044110904 | 0.017995932 | 2.12984245 | 1.090746715 |
| RNF32-AS1 | up | 0.000119265 | 2.64602E-05 | 2.311177554 | 1.208628098 |
| PRRT4 | up | 8.52357E-21 | 3.85826E-22 | 4.510888765 | 2.173411711 |
| DUXAP9 | up | 0.005251868 | 0.001650855 | 6.722421536 | 2.748981011 |
| RFPL1S | up | 3.4628E-05 | 6.95982E-06 | 2.582576709 | 1.368811201 |
| LINC01980 | up | 3.76837E-08 | 4.90536E-09 | 447.1404467 | 8.804584243 |
| NTF4 | up | 4.24731E-09 | 4.88571E-10 | 5.614192128 | 2.489078437 |
| ELFN1 | up | 0.003949829 | 0.00119963 | 3.030852441 | 1.599723615 |
| LINC02660 | up | 2.29377E-05 | 4.45826E-06 | 165.4595618 | 7.370334856 |
| LINC00623 | up | 4.99446E-16 | 3.06689E-17 | 21.22445477 | 4.407655588 |
| KRT16P6 | up | 0.029437447 | 0.01133019 | 5.106663916 | 2.352381114 |
| LINC01748 | up | 0.0002506 | 5.9274E-05 | 108.8887502 | 6.7667111 |
| CYP4F26P | up | 0.000190885 | 4.4126E-05 | 3.234616759 | 1.69359479 |
| PSMD10P2 | up | 0.013485604 | 0.004717586 | 2.310710598 | 1.208336583 |
| CACTIN-AS1 | up | 0.003377078 | 0.001006856 | 3.181471567 | 1.669694228 |
| ERVMER34-1 | up | 9.41611E-10 | 1.0178E-10 | 23.70852009 | 4.567333706 |
| MUC12-AS1 | up | 0.031568404 | 0.012264892 | 3.349123022 | 1.743783371 |
| KRT16P2 | up | 0.017348246 | 0.00624121 | 4.367811466 | 2.126910585 |
| RBM26-AS1 | up | 0.000814635 | 0.000211999 | 2.758036777 | 1.463641695 |
| TP73-AS1 | up | 2.8493E-08 | 3.64157E-09 | 2.740559971 | 1.454470705 |
| LTB | up | 6.10205E-11 | 5.86754E-12 | 4.03838276 | 2.013777657 |
| PHBP11 | up | 0.015413002 | 0.005472868 | 45.13189385 | 5.496075414 |
| FRMD8P1 | up | 0.04287177 | 0.017400245 | 6.919734081 | 2.790716597 |
| MIR646HG | up | 3.96717E-06 | 6.79514E-07 | 221.5165629 | 7.791270763 |
| LINC01546 | up | 4.56593E-05 | 9.34742E-06 | 146.5781482 | 7.195526233 |
| NUTM2E | up | 0.038224473 | 0.015295014 | 4.952354755 | 2.308114664 |
| FNDC10 | up | 6.51135E-07 | 1.00164E-07 | 2.032075418 | 1.022953947 |
| LINC00659 | up | 0.005839688 | 0.001857427 | 2.089767629 | 1.063342531 |
| ACTBP1 | up | 0.002835216 | 0.000828907 | 67.7163505 | 6.081432318 |
| RUNX3-AS1 | up | 0.048606693 | 0.020145712 | 9.760146249 | 3.286902766 |
| MYOSLID | up | 2.00128E-20 | 9.25887E-22 | 61.51587903 | 5.942886955 |
| ITGB1-DT | up | 0.04496382 | 0.018405357 | 2.603286378 | 1.380334026 |
| KIF25-AS1 | up | 0.022650159 | 0.008441563 | 41.42963703 | 5.372591276 |
| KRT16P5 | up | 0.011538975 | 0.003966238 | 11.29094557 | 3.497094406 |
| TMEM254-AS1 | up | 1.56456E-07 | 2.2151E-08 | 3.847246731 | 1.943826355 |
| LINC02028 | up | 0.037292218 | 0.014874931 | 5.321117826 | 2.41172935 |
| LINC02643 | up | 0.025463861 | 0.009634793 | 3.737034849 | 1.901894017 |
| LINC01721 | up | 0.004105091 | 0.001251965 | 7.022515478 | 2.811987899 |
| CNOT6LP1 | up | 0.005125794 | 0.001604488 | 2.731508268 | 1.44969779 |
| LINC00856 | up | 0.031307438 | 0.012150306 | 10.83013365 | 3.436979141 |
| LRRC37A6P | up | 0.001411672 | 0.000386668 | 2.685117873 | 1.424985422 |
| ANKRD18B | up | 5.04689E-06 | 8.80109E-07 | 2.20213434 | 1.138902482 |
| PSMG3-AS1 | up | 0.001033178 | 0.000274468 | 2.135616174 | 1.094652381 |
| LINC02580 | up | 0.04187187 | 0.016952593 | 3.141015657 | 1.651231134 |
| RHOQP3 | up | 9.8725E-05 | 2.15606E-05 | 6.213158916 | 2.635326955 |
| LINC01293 | up | 2.33255E-23 | 8.97655E-25 | 8.11159989 | 3.019986492 |
| MIR205HG | up | 5.79781E-33 | 1.43261E-34 | 2.051742617 | 1.036849762 |
| LINC02541 | up | 1.09706E-13 | 8.31693E-15 | 15.22442403 | 3.928315744 |
| SPRY4-AS1 | up | 0.005461661 | 0.001723405 | 3.808940611 | 1.929389794 |
| APCDD1L-DT | up | 7.04944E-23 | 2.79072E-24 | 5.732105097 | 2.519065061 |
| MANCR | up | 3.4443E-06 | 5.84342E-07 | 118.6687516 | 6.890796278 |
| LINC00574 | up | 0.049663812 | 0.020658224 | 13.54411715 | 3.759594452 |
| LINC01819 | up | 2.66047E-08 | 3.37227E-09 | 6.477529878 | 2.695443765 |
| TRAF3IP2-AS1 | up | 0.000651552 | 0.000166977 | 3.340381326 | 1.740012805 |
| HS1BP3-IT1 | up | 0.012510656 | 0.004339036 | 18.21637527 | 4.187164012 |
| LRRC8C-DT | up | 0.005348418 | 0.001685141 | 7.396215145 | 2.886787191 |
| IMPDH1P10 | up | 0.00303208 | 0.000890766 | 5.041893304 | 2.333965588 |
| MXRA7P1 | up | 0.002716316 | 0.000791146 | 4.576604463 | 2.194277611 |
| LINC02777 | up | 2.84444E-06 | 4.77488E-07 | 3.914617786 | 1.968871453 |
| RHOQP2 | up | 0.001207492 | 0.000326044 | 13.99172205 | 3.80650163 |
| RPS11P5 | up | 0.000274565 | 6.54332E-05 | 2.133604662 | 1.093292882 |
| SNHG15 | up | 2.4284E-13 | 1.89461E-14 | 2.366483257 | 1.242744715 |
| ERVH48-1 | up | 0.000204524 | 4.75799E-05 | 112.8115894 | 6.817771476 |
| CCDC144NL-AS1 | up | 1.34692E-45 | 1.87653E-47 | 112.5458485 | 6.814369031 |
| LINC01687 | up | 0.005008028 | 0.001563939 | 7.018036377 | 2.811067426 |
| CNIH3-AS2 | up | 0.035578721 | 0.014075489 | 5.786432007 | 2.532674037 |
| AGGF1P2 | up | 0.003478881 | 0.001040866 | 4.604510208 | 2.203047702 |
| LINC00513 | up | 0.001481588 | 0.000407844 | 2.706506761 | 1.436431992 |
| GAS6-AS1 | up | 0.013930221 | 0.004896559 | 3.123400468 | 1.643117557 |
| SLC26A4-AS1 | up | 0.009709974 | 0.003273753 | 2.475654755 | 1.307810136 |
| LINC01503 | up | 1.9458E-86 | 1.03321E-88 | 15.53876464 | 3.957799906 |
| LINC01694 | up | 0.005161086 | 0.001616621 | 2.457122693 | 1.296969898 |
| LINC02535 | up | 0.000268769 | 6.39246E-05 | 4.806000227 | 2.264836716 |
| LNCTAM34A | up | 0.001055361 | 0.000281026 | 4.573054659 | 2.193158163 |
| SEMA3F-AS1 | up | 0.011475663 | 0.003942666 | 48.79811141 | 5.608753408 |
| ANKRD65 | up | 6.92913E-28 | 2.10394E-29 | 58.2216496 | 5.863483811 |
| C12orf75 | up | 1.00196E-42 | 1.55396E-44 | 13.17264471 | 3.719473124 |
| L3MBTL2-AS1 | up | 0.011835167 | 0.004078625 | 6.805616287 | 2.766725812 |
| LINC01828 | up | 0.029534401 | 0.011381481 | 37.57931756 | 5.231866962 |
| ELFN1-AS1 | up | 9.3427E-13 | 7.66729E-14 | 5.784407176 | 2.53216911 |
| SCN1A-AS1 | up | 9.36783E-05 | 2.03205E-05 | 12.44347328 | 3.637317328 |
| ZNF853 | up | 0.006503919 | 0.002095027 | 5.15293479 | 2.365394335 |
| MAP3K2-DT | up | 0.000496264 | 0.000124086 | 2.202393792 | 1.139072449 |
| LINC01010 | up | 0.029534401 | 0.011381481 | 37.57931756 | 5.231866962 |
| LINC01844 | up | 1.44927E-06 | 2.33532E-07 | 6.79586847 | 2.76465793 |
| LINC01186 | up | 0.004198408 | 0.001283611 | 2.166735139 | 1.11552281 |
| LINC01792 | up | 1.23229E-07 | 1.71747E-08 | 5.131913487 | 2.35949685 |
| CKMT1B | up | 9.86686E-06 | 1.8052E-06 | 8.42756557 | 3.075115947 |
| CECR7 | up | 1.90278E-05 | 3.63929E-06 | 93.64064792 | 6.549063012 |
| C10orf143 | up | 1.5985E-32 | 4.01704E-34 | 52.36431703 | 5.710512136 |
| PKN2-AS1 | up | 0.021530854 | 0.00796894 | 10.40721822 | 3.379512592 |
| GPAT2P1 | up | 2.60343E-05 | 5.09708E-06 | 3.881890972 | 1.956759599 |
| UNC5B-AS1 | up | 6.65011E-10 | 7.10077E-11 | 6.452750782 | 2.689914307 |
| PRKCQ-AS1 | up | 3.83939E-07 | 5.73658E-08 | 3.777630935 | 1.917481761 |
| LINC00649 | up | 4.29603E-05 | 8.75197E-06 | 12.86927041 | 3.685858361 |
| LINC02806 | up | 1.16161E-08 | 1.40805E-09 | 14.54413746 | 3.862365835 |
| LINC00707 | up | 1.08578E-08 | 1.31235E-09 | 2.554595349 | 1.353094784 |
| LINC01119 | up | 7.80671E-10 | 8.3932E-11 | 3.249030156 | 1.700009134 |
| AQP1 | up | 1.69887E-20 | 7.80616E-22 | 4.748899563 | 2.247593244 |
| AADACP1 | up | 6.56096E-05 | 1.38456E-05 | 3.42865755 | 1.777643817 |
| PCDHGC5 | up | 0.010073801 | 0.003410189 | 11.11799144 | 3.474824271 |
| ARHGEF25 | up | 2.41649E-16 | 1.4394E-17 | 2.448697303 | 1.292014445 |
| CD302 | up | 1.37986E-06 | 2.21768E-07 | 4.854089275 | 2.279200642 |
| HOGA1 | up | 0.00011364 | 2.51119E-05 | 4.053048204 | 2.019007334 |
| LASTR | up | 6.75095E-34 | 1.61489E-35 | 6.030242185 | 2.592215945 |
| CRYZL2P | up | 6.40756E-05 | 1.34747E-05 | 2.284918981 | 1.192143011 |
| STON1 | up | 5.4787E-125 | 1.613E-127 | 27.57596518 | 4.785339477 |
| AOX2P | up | 0.047824238 | 0.019777375 | 2.75458214 | 1.461833484 |
| LINC01322 | up | 1.33225E-07 | 1.86729E-08 | 371.834655 | 8.538517426 |
| DBNDD2 | up | 0.008918341 | 0.002976907 | 2.199395698 | 1.137107186 |
| DUXAP10 | up | 1.05433E-30 | 2.87126E-32 | 16.46917798 | 4.041696643 |
| KCNK15-AS1 | up | 0.012972836 | 0.004509563 | 3.697077845 | 1.886385421 |
| PTCHD4 | up | 0.029985825 | 0.01158382 | 37.54262118 | 5.230457476 |
| USP2-AS1 | up | 0.021974339 | 0.008159653 | 2.523941319 | 1.335678369 |
| DDX11-AS1 | up | 1.86411E-19 | 9.15345E-21 | 4.195143266 | 2.06872008 |
| KLRK1-AS1 | up | 0.002160867 | 0.000616644 | 10.33243248 | 3.369108031 |
| CASC8 | up | 0.002305547 | 0.000661689 | 2.146297958 | 1.101850371 |
| PICART1 | up | 0.002648462 | 0.00077013 | 2.869621645 | 1.520860533 |
| RASSF8-AS1 | up | 2.5193E-20 | 1.17746E-21 | 4.671371937 | 2.223846318 |
| SOCS2-AS1 | up | 3.55132E-11 | 3.34949E-12 | 3.740750762 | 1.903327846 |
| UBA6-AS1 | up | 8.36163E-09 | 9.96134E-10 | 2.676868467 | 1.420546251 |
| RRN3P1 | up | 0.00015367 | 3.47962E-05 | 2.639830827 | 1.400445478 |
| TNXA | up | 8.01664E-13 | 6.5411E-14 | 14.73019066 | 3.880704199 |
| LINC02434 | up | 1.76812E-14 | 1.2577E-15 | 28.27319222 | 4.821362877 |
| FAM198B-AS1 | up | 0.001577749 | 0.000437715 | 10.46268132 | 3.38718072 |
| LINC02428 | up | 0.043261562 | 0.017576644 | 2.474065787 | 1.306883863 |
| LINC02057 | up | 0.012129148 | 0.004190139 | 2.387313614 | 1.255388101 |
| LINC00964 | up | 0.021166813 | 0.007809721 | 41.3555934 | 5.370010564 |
| SH3TC2-DT | up | 0.003437899 | 0.001027701 | 2.207759425 | 1.142582973 |
| FZD10-AS1 | up | 1.03431E-09 | 1.12398E-10 | 3.623779679 | 1.857495244 |
| GPR162 | up | 2.41884E-21 | 1.06947E-22 | 16.86969011 | 4.076361567 |
| ATP6V1E2 | up | 1.03741E-61 | 9.48997E-64 | 6.357947167 | 2.668561028 |
| ZBED3-AS1 | up | 0.003298665 | 0.000980009 | 5.440567461 | 2.443757135 |
| ZNF674 | up | 0.000546378 | 0.000137851 | 2.782124512 | 1.476186988 |
| ALG1L7P | up | 0.004704901 | 0.00145963 | 60.16366564 | 5.910820565 |
| LINC00958 | up | 0.007338107 | 0.002391896 | 31.06039362 | 4.957004208 |
| LINC01267 | up | 0.007666303 | 0.002510965 | 19.75358587 | 4.304042665 |
| MSNP1 | up | 4.457E-55 | 4.78012E-57 | 5.396854446 | 2.432118778 |
| LINC01605 | up | 2.36341E-33 | 5.77775E-35 | 27.26050758 | 4.76874052 |
| TRNP1 | up | 1.76569E-41 | 2.92409E-43 | 4.579898094 | 2.195315498 |
| IKBKB-DT | up | 0.021223317 | 0.007834222 | 41.3189467 | 5.368731572 |
| LNCOC1 | up | 0.002446602 | 0.00070603 | 2.834700369 | 1.503196249 |
| ALG1L13P | up | 0.021775282 | 0.008074469 | 41.28215391 | 5.367446341 |
| CASC19 | up | 4.24324E-13 | 3.38415E-14 | 187.0535518 | 7.54730755 |
| LINC02584 | up | 0.004892786 | 0.001524092 | 33.96365678 | 5.085919893 |
| NAV2-AS2 | up | 0.015270617 | 0.005420704 | 45.09522263 | 5.494902698 |
| RASSF10-DT | up | 9.19869E-05 | 1.99101E-05 | 14.30871945 | 3.83882266 |
| MIR100HG | up | 2.27205E-08 | 2.84887E-09 | 3.079954233 | 1.622908913 |
| HCAR3 | up | 4.67684E-13 | 3.75455E-14 | 2.312650385 | 1.209547183 |
| MIR9-3HG | up | 1.24907E-19 | 6.06116E-21 | 2.077924314 | 1.055143107 |
| AGAP2-AS1 | up | 6.22701E-09 | 7.33321E-10 | 2.250397527 | 1.170179873 |
| CAPNS2 | up | 0.001587014 | 0.000440438 | 18.23674113 | 4.18877604 |
| ODC1-DT | up | 0.020410394 | 0.007494148 | 4.599821505 | 2.201577879 |
| LINC02457 | up | 5.59412E-15 | 3.76157E-16 | 20.82587027 | 4.380304879 |
| NBEAP1 | up | 0.006758578 | 0.002185509 | 7.011537617 | 2.809730859 |
| LINC01629 | up | 3.27439E-12 | 2.81975E-13 | 11.43529091 | 3.515421163 |
| ITGB3 | up | 0.018906988 | 0.006874545 | 2.833980664 | 1.502829915 |
| LINC01833 | up | 3.5725E-18 | 1.89885E-19 | 5.358675068 | 2.421876339 |
| DDX59-AS1 | up | 0.039058823 | 0.015680205 | 7.802290595 | 2.963897732 |
| CARMAL | up | 0.003342876 | 0.000995253 | 20.53843929 | 4.360254651 |
| CCDC187 | up | 1.57636E-13 | 1.20748E-14 | 4.63912012 | 2.213851202 |
| KDM7A-DT | up | 0.007641953 | 0.002502588 | 3.626108089 | 1.85842193 |
| KCNJ18 | up | 9.91219E-14 | 7.49889E-15 | 3.904265733 | 1.965051249 |
| LINC00622 | up | 0.004393607 | 0.001351506 | 2.522244597 | 1.334708189 |
| TBILA | up | 1.43325E-07 | 2.01639E-08 | 3.714097983 | 1.893011876 |
| LINC01976 | up | 0.001890219 | 0.000532453 | 75.01094254 | 6.229029165 |
| SSTR5-AS1 | up | 2.75462E-05 | 5.42117E-06 | 12.72034041 | 3.669065374 |
| LINC02582 | up | 5.34891E-06 | 9.36995E-07 | 4.567536583 | 2.191416284 |
| LINC02141 | up | 0.021775282 | 0.008074469 | 41.28215391 | 5.367446341 |
| LINC01569 | up | 0.017381406 | 0.006256795 | 2.23908789 | 1.162911159 |
| LINC01977 | up | 0.000311502 | 7.50382E-05 | 2.841752582 | 1.506780952 |
| IKBKE | up | 4.18701E-26 | 1.37138E-27 | 2.554572226 | 1.353081726 |
| RN7SL4P | up | 0.011174094 | 0.003824958 | 52.42305813 | 5.712129611 |
| ANXA8L1 | up | 1.37719E-61 | 1.27431E-63 | 2.590874332 | 1.373439041 |
| ZNF407-AS1 | up | 9.55996E-05 | 2.08E-05 | 2.645883686 | 1.403749642 |
| ANXA8 | up | 1.69493E-88 | 8.5544E-91 | 2.97726415 | 1.573987227 |
| MAFG-DT | up | 0.024457138 | 0.00920759 | 2.161286693 | 1.111890457 |
| ZNF561-AS1 | up | 0.00093484 | 0.000245986 | 5.575071528 | 2.478990315 |
| SCAT1 | up | 1.04548E-07 | 1.44227E-08 | 14.55166255 | 3.863112087 |
| LINC01842 | up | 0.013004448 | 0.004523286 | 11.59969 | 3.536014345 |
| MIR2117HG | up | 0.00236184 | 0.000679832 | 71.27171726 | 6.155257781 |
| DNAH17-AS1 | up | 0.000812955 | 0.00021152 | 2.53819526 | 1.343803058 |
| SINHCAFP2 | up | 1.36762E-17 | 7.48484E-19 | 4.917208685 | 2.297839584 |
| LINC01711 | up | 5.3984E-06 | 9.468E-07 | 5.665947944 | 2.502317347 |
| TRABD2B | up | 3.54534E-38 | 6.87781E-40 | 11.87701255 | 3.570100093 |
| NAV2-AS6 | up | 0.033092793 | 0.012952833 | 14.25873249 | 3.833773836 |
| MMP28 | up | 1.444E-14 | 1.01879E-15 | 23.08575302 | 4.528930886 |
| LINC01145 | up | 2.91973E-06 | 4.90741E-07 | 2.638176146 | 1.399540894 |
| HK2-DT | up | 1.88762E-07 | 2.70625E-08 | 11.99339583 | 3.584168299 |
| APP-DT | up | 0.00467271 | 0.00144866 | 60.12703137 | 5.909941826 |
| LINC01138 | up | 0.007072032 | 0.002297732 | 2.028971246 | 1.02074842 |
| CBSL | up | 0.009035256 | 0.003021569 | 8.033895931 | 3.006099773 |
| ADRA2B | up | 0.0259441 | 0.009830142 | 2.483618564 | 1.31244362 |
| ZNF280B | up | 2.40017E-08 | 3.02088E-09 | 2.157306246 | 1.109230993 |
| PRAG1 | up | 1.48724E-20 | 6.82594E-22 | 2.211683505 | 1.145144949 |
| FLJ16779 | up | 0.006525813 | 0.002103109 | 13.1221992 | 3.713937622 |
| ARHGAP23 | up | 1.2056E-51 | 1.41978E-53 | 2.903626286 | 1.537855782 |
| RN7SL1 | up | 0.029340197 | 0.011288132 | 14.41860114 | 3.849859299 |
| FAM25C | up | 8.95775E-05 | 1.9318E-05 | 6.709293798 | 2.74616092 |
| RAB7B | up | 3.99789E-21 | 1.78445E-22 | 12.80431128 | 3.67855775 |
| LINC00869 | up | 0.017604377 | 0.006350015 | 4.2925544 | 2.101836418 |
| NLRP3P1 | up | 5.88606E-09 | 6.90075E-10 | 32.89398916 | 5.039752074 |
| CHMP1B2P | up | 0.000217695 | 5.10103E-05 | 18.22663475 | 4.187976311 |
| NR2E3 | up | 0.023594756 | 0.008849429 | 3.604871607 | 1.849947877 |
| RN7SL3 | up | 0.003423132 | 0.001022566 | 23.15410658 | 4.533196185 |
| LINC00624 | up | 0.032936014 | 0.012882811 | 6.707870449 | 2.745854826 |
| LINC01451 | up | 2.84906E-05 | 5.63042E-06 | 165.6861467 | 7.372309172 |
| LINC01126 | up | 0.005214189 | 0.001637092 | 2.805464587 | 1.488239702 |
| SH3PXD2A-AS1 | up | 7.10717E-07 | 1.09927E-07 | 2.41097006 | 1.269613735 |
| LSP1P5 | up | 2.86963E-11 | 2.68542E-12 | 2.851435476 | 1.511688387 |
| C13orf46 | up | 0.019200291 | 0.006991284 | 2.503436128 | 1.323909647 |
| EXOC3L2 | up | 0.000474291 | 0.000118143 | 54.34158199 | 5.763984661 |
