# Supplementary material for: Restoring expression of tumour suppressor PTEN by engineered circular RNA‐enhanced Osimertinib sensitivity in non‐small cell lung cancer
Source: Clin Transl Med. 2024 Aug 21;14(8):e1792. doi: 10.1002/ctm2.1792 (PMC11337465; doi:10.1002/ctm2.1792)
Supplement: Supplementary file 4 — TABLE S3. List of down‐regulated genes in the group of PC9OR_cPTEN_NeoAna versus PC9 Osimertinib‐resistance (PC9OR). [file CTM2-14-e1792-s003.docx]

Table S3. The list of down-regulated genes in the group of PC9OR_cPTEN_NeoAna vs. PC9OR.

| Gene name | Style | FDR | P value | Fold Change | log2FC |
| --- | --- | --- | --- | --- | --- |
| ALDH3B1 | down | 4.56E-22 | 7.79E-24 | 0.45504 | -1.13593 |
| ACP3 | down | 0.016025 | 0.004006 | 0.373946 | -1.4191 |
| ANK1 | down | 4.89E-07 | 3.81E-08 | 0.346969 | -1.52712 |
| DNAH5 | down | 0.001172 | 0.000199 | 0.48909 | -1.03183 |
| TG | down | 0.034317 | 0.009957 | 0.422451 | -1.24314 |
| ALX4 | down | 1.93E-07 | 1.41E-08 | 0.436371 | -1.19637 |
| CCN5 | down | 0.043172 | 0.013172 | 0.350397 | -1.51294 |
| FECH | down | 2.75E-23 | 4.41E-25 | 0.451576 | -1.14696 |
| PRR11 | down | 4.06E-50 | 2.43E-52 | 0.416521 | -1.26354 |
| ST6GALNAC2 | down | 2.61E-09 | 1.37E-10 | 0.433774 | -1.20498 |
| SLC24A1 | down | 6.48E-11 | 2.75E-12 | 0.456238 | -1.13214 |
| SEMA5B | down | 7.65E-08 | 5.17E-09 | 0.336158 | -1.57279 |
| BCKDHB | down | 6.33E-08 | 4.21E-09 | 0.434258 | -1.20338 |
| FAT2 | down | 6.07E-68 | 2.29E-70 | 0.28187 | -1.8269 |
| BLNK | down | 0.004102 | 0.000828 | 0.372087 | -1.42629 |
| EFHC1 | down | 0.002879 | 0.000553 | 0.40293 | -1.3114 |
| SCD | down | 2.9E-105 | 6.9E-108 | 0.365228 | -1.45313 |
| P2RX6 | down | 0.000197 | 2.72E-05 | 0.433418 | -1.20617 |
| RAB36 | down | 2.12E-11 | 8.52E-13 | 0.403292 | -1.3101 |
| ASB9 | down | 0.000411 | 6.15E-05 | 0.456665 | -1.13079 |
| IL21R | down | 0.002657 | 0.000506 | 0.073479 | -3.76653 |
| ALDH3A1 | down | 6.45E-14 | 1.98E-15 | 0.34755 | -1.52471 |
| MAP2K6 | down | 9.42E-08 | 6.5E-09 | 0.197263 | -2.34181 |
| MAPK10 | down | 0.009896 | 0.002272 | 0.284847 | -1.81174 |
| CPE | down | 4.3E-12 | 1.59E-13 | 0.436889 | -1.19466 |
| CRYAB | down | 0.003724 | 0.000741 | 0.270489 | -1.88636 |
| SIAE | down | 2E-11 | 7.99E-13 | 0.480124 | -1.05852 |
| SELPLG | down | 0.014746 | 0.003639 | 0.487009 | -1.03798 |
| TRPV4 | down | 0.016205 | 0.004062 | 0.418467 | -1.25682 |
| EFEMP1 | down | 1.3E-47 | 8.19E-50 | 0.486358 | -1.03991 |
| RPL22 | down | 1.84E-23 | 2.88E-25 | 0.489806 | -1.02972 |
| OSCP1 | down | 3E-05 | 3.44E-06 | 0.268908 | -1.89482 |
| NID1 | down | 9.73E-10 | 4.85E-11 | 0.466393 | -1.10038 |
| ALDH6A1 | down | 2.09E-14 | 6.14E-16 | 0.490949 | -1.02635 |
| EGR1 | down | 3.71E-05 | 4.34E-06 | 0.423775 | -1.23863 |
| ACO1 | down | 2.51E-20 | 4.9E-22 | 0.446135 | -1.16445 |
| NRK | down | 0.004237 | 0.000859 | 0.437092 | -1.19399 |
| FNDC11 | down | 4.54E-09 | 2.48E-10 | 0.301877 | -1.72797 |
| FLRT1 | down | 9.19E-06 | 9.4E-07 | 0.316614 | -1.6592 |
| FGD3 | down | 1.12E-11 | 4.37E-13 | 0.495936 | -1.01177 |
| IFT22 | down | 6.73E-05 | 8.38E-06 | 0.480078 | -1.05866 |
| ATP8B3 | down | 0.000322 | 4.68E-05 | 0.498825 | -1.00339 |
| H19 | down | 1.08E-17 | 2.39E-19 | 0.289279 | -1.78947 |
| SERPINF1 | down | 4.93E-09 | 2.71E-10 | 0.415609 | -1.2667 |
| ALDH3B2 | down | 9.21E-20 | 1.84E-21 | 0.315653 | -1.66359 |
| ZBED3 | down | 1.37E-13 | 4.34E-15 | 0.38024 | -1.39502 |
| VAV3 | down | 1.47E-15 | 3.99E-17 | 0.411625 | -1.2806 |
| VTCN1 | down | 9.23E-10 | 4.58E-11 | 0.174991 | -2.51465 |
| ZFHX2 | down | 0.021406 | 0.005658 | 0.479621 | -1.06003 |
| TMPRSS4 | down | 3.4E-09 | 1.82E-10 | 0.475088 | -1.07373 |
| CLCA2 | down | 4.93E-10 | 2.37E-11 | 0.1983 | -2.33424 |
| IFT172 | down | 5.84E-13 | 1.96E-14 | 0.253655 | -1.97906 |
| EMILIN1 | down | 0.028947 | 0.008107 | 0.367162 | -1.44551 |
| MMAB | down | 1.28E-11 | 5.03E-13 | 0.435755 | -1.19841 |
| CLBA1 | down | 0.048621 | 0.015232 | 0.480851 | -1.05634 |
| RNF157 | down | 0.018113 | 0.004636 | 0.373002 | -1.42274 |
| CFAP74 | down | 0.004567 | 0.000936 | 0.362701 | -1.46315 |
| WDR31 | down | 2.59E-06 | 2.36E-07 | 0.443384 | -1.17337 |
| TTC12 | down | 0.000114 | 1.5E-05 | 0.479735 | -1.05969 |
| ANK3 | down | 4.66E-29 | 5.7E-31 | 0.499479 | -1.0015 |
| **AKR1C2** | **down** | **6.3E-143** | **1E-145** | **0.394502** | **-1.34189** |
| IGSF10 | down | 0.016701 | 0.004206 | 0.192505 | -2.37703 |
| HYDIN | down | 0.025908 | 0.007099 | 0.396661 | -1.33402 |
| PAFAH2 | down | 2.21E-05 | 2.43E-06 | 0.458591 | -1.12472 |
| ALDH4A1 | down | 6.08E-10 | 2.96E-11 | 0.406939 | -1.29711 |
| FAM86C2P | down | 0.007767 | 0.001729 | 0.498002 | -1.00578 |
| CBS | down | 0.003766 | 0.000752 | 0.448536 | -1.1567 |
| C9orf116 | down | 9.63E-05 | 1.24E-05 | 0.361318 | -1.46866 |
| MEIOB | down | 0.008358 | 0.001882 | 0.451711 | -1.14653 |
| UBXN10 | down | 1.27E-08 | 7.49E-10 | 0.317658 | -1.65445 |
| OBSCN-AS1 | down | 0.000944 | 0.000157 | 0.408087 | -1.29305 |
| VSNL1 | down | 0.014748 | 0.003641 | 0.372879 | -1.42322 |
| GLB1L | down | 0.001553 | 0.000275 | 0.348831 | -1.5194 |
| LZTFL1 | down | 7.34E-07 | 5.9E-08 | 0.394365 | -1.3424 |
| IFT122 | down | 1.99E-10 | 9.04E-12 | 0.485278 | -1.04312 |
| HPGD | down | 0.021699 | 0.005751 | 0.366267 | -1.44903 |
| GASK1B | down | 0.017872 | 0.004557 | 0.348363 | -1.52134 |
| F2RL2 | down | 8.23E-11 | 3.56E-12 | 0.410414 | -1.28485 |
| DEFB1 | down | 0.040367 | 0.012115 | 0.330477 | -1.59738 |
| TMEM67 | down | 8E-06 | 8.1E-07 | 0.366057 | -1.44986 |
| ARMC3 | down | 6.2E-05 | 7.65E-06 | 0.350878 | -1.51096 |
| SYNPO2L | down | 0.000239 | 3.38E-05 | 0.357881 | -1.48245 |
| SERPINF2 | down | 6.45E-05 | 7.99E-06 | 0.479588 | -1.06013 |
| CYB5D2 | down | 2.91E-05 | 3.32E-06 | 0.496823 | -1.0092 |
| GGT6 | down | 8.75E-08 | 5.98E-09 | 0.454571 | -1.13742 |
| TCTN2 | down | 3.83E-07 | 2.93E-08 | 0.422231 | -1.2439 |
| LCMT2 | down | 0.006171 | 0.001322 | 0.468379 | -1.09425 |
| ZNF608 | down | 9.92E-08 | 6.88E-09 | 0.314041 | -1.67097 |
| INPP5D | down | 4.32E-28 | 5.58E-30 | 0.414415 | -1.27085 |
| PCSK9 | down | 0.034852 | 0.010162 | 0.349099 | -1.51829 |
| FRMPD2 | down | 0.000367 | 5.41E-05 | 0.243081 | -2.04049 |
| CEL | down | 1.86E-06 | 1.64E-07 | 0.296992 | -1.7515 |
| KRT13 | down | 0.002802 | 0.000536 | 0.465638 | -1.10272 |
| LIPT2 | down | 0.011581 | 0.002735 | 0.412774 | -1.27658 |
| DLEU1 | down | 0.01103 | 0.002585 | 0.490075 | -1.02893 |
| GPX2 | down | 8.14E-46 | 5.62E-48 | 0.469293 | -1.09144 |
| ACBD7 | down | 3.57E-08 | 2.27E-09 | 0.325824 | -1.61784 |
| DNAJC30 | down | 0.017185 | 0.004351 | 0.433619 | -1.2055 |
| CCDC121 | down | 0.021009 | 0.005537 | 0.201244 | -2.31298 |
| TENM3-AS1 | down | 0.018294 | 0.00469 | 0.487921 | -1.03528 |
| MCMDC2 | down | 0.008097 | 0.001816 | 0.332172 | -1.59 |
| PSAPL1 | down | 0.000547 | 8.46E-05 | 0.314317 | -1.66971 |
| ZNF771 | down | 0.023884 | 0.006443 | 0.478817 | -1.06245 |
| SLC47A2 | down | 8.7E-22 | 1.51E-23 | 0.354494 | -1.49617 |
| ZFP3 | down | 0.010816 | 0.002519 | 0.467167 | -1.09799 |
| TMIE | down | 0.016434 | 0.004129 | 0.453576 | -1.14058 |
| FAM87A | down | 0.007136 | 0.001566 | 0.18595 | -2.42702 |
| FANCF | down | 4.56E-11 | 1.88E-12 | 0.442486 | -1.1763 |
| GOLGA8G | down | 0.041067 | 0.012372 | 0.23923 | -2.06353 |
| RP1L1 | down | 5.97E-06 | 5.79E-07 | 0.186321 | -2.42414 |
| MAP7D2 | down | 0.000119 | 1.56E-05 | 0.375543 | -1.41295 |
| PPIL6 | down | 0.042659 | 0.01297 | 0.439054 | -1.18753 |
| METTL7A | down | 5.43E-06 | 5.24E-07 | 0.098455 | -3.34439 |
| PBX1 | down | 6.13E-33 | 6.34E-35 | 0.404221 | -1.30678 |
| FAM86B1 | down | 0.009792 | 0.002244 | 0.445697 | -1.16586 |
| DYNC2H1 | down | 8.36E-09 | 4.81E-10 | 0.422329 | -1.24356 |
| PAX5 | down | 0.005893 | 0.001252 | 0.335857 | -1.57408 |
| TTC30B | down | 0.000661 | 0.000104 | 0.154274 | -2.69644 |
| WDR5B | down | 0.004138 | 0.000837 | 0.428488 | -1.22267 |
| TTC30A | down | 0.032477 | 0.009324 | 0.201094 | -2.31406 |
| AKR1B10 | down | 1.58E-84 | 4.85E-87 | 0.45342 | -1.14108 |
| PAPSS2 | down | 8.8E-08 | 6.02E-09 | 0.442937 | -1.17483 |
| MUC2 | down | 0.008809 | 0.001997 | 0.348329 | -1.52148 |
| MT-ND3 | down | 3.68E-15 | 1.01E-16 | 0.417043 | -1.26173 |
| CES1 | down | 0.031256 | 0.008917 | 0.322097 | -1.63443 |
| MT-CO3 | down | 7.58E-97 | 2E-99 | 0.472619 | -1.08125 |
| PPP1R10 | down | 9.46E-29 | 1.19E-30 | 0.457839 | -1.12709 |
| VIT | down | 0.00139 | 0.000243 | 0.131093 | -2.93134 |
| LNP1 | down | 0.004436 | 0.000906 | 0.276598 | -1.85414 |
| DIO2 | down | 0.008967 | 0.002037 | 0.497823 | -1.00629 |
| MAP10 | down | 0.000433 | 6.52E-05 | 0.388577 | -1.36373 |
| PLXNA4 | down | 8.03E-07 | 6.53E-08 | 0.367912 | -1.44257 |
| C5orf66 | down | 0.049787 | 0.015652 | 0.420127 | -1.2511 |
| LINC00365 | down | 0.030383 | 0.008626 | 0.414953 | -1.26898 |
| EIF4BP3 | down | 0.006683 | 0.001448 | 0.439029 | -1.18761 |
| EIF4BP7 | down | 2.54E-05 | 2.85E-06 | 0.476763 | -1.06866 |
| SGMS1-AS1 | down | 0.000594 | 9.27E-05 | 0.31992 | -1.64422 |
| GTF2H2B | down | 0.008365 | 0.001885 | 0.353549 | -1.50002 |
| DANCR | down | 4.5E-09 | 2.45E-10 | 0.4671 | -1.0982 |
| RPL3P2 | down | 7.82E-14 | 2.42E-15 | 0.448428 | -1.15705 |
| LINC01721 | down | 0.003517 | 0.000694 | 0.461371 | -1.116 |
| LRRC37A6P | down | 0.000503 | 7.71E-05 | 0.302198 | -1.72643 |
| PSMG3-AS1 | down | 2.64E-06 | 2.41E-07 | 0.317431 | -1.65548 |
| TMEM44-AS1 | down | 0.024096 | 0.006521 | 0.41874 | -1.25587 |
| PIK3CD-AS2 | down | 0.018021 | 0.004607 | 0.254027 | -1.97694 |
| LINC01819 | down | 0.023746 | 0.006398 | 0.498033 | -1.00569 |
| NPIPA9 | down | 0.004429 | 0.000904 | 0.18977 | -2.39767 |
| GAS6-AS1 | down | 0.029022 | 0.008141 | 0.32721 | -1.61171 |
| LINC01694 | down | 0.028345 | 0.0079 | 0.451833 | -1.14614 |
| BRD3OS | down | 3.12E-07 | 2.35E-08 | 0.478246 | -1.06418 |
| OLMALINC | down | 4.4E-26 | 6.4E-28 | 0.327211 | -1.6117 |
| CKMT1B | down | 0.000316 | 4.57E-05 | 0.159046 | -2.65249 |
| LINC00649 | down | 0.01506 | 0.003727 | 0.25684 | -1.96106 |
| LINC02806 | down | 0.042307 | 0.012841 | 0.470389 | -1.08807 |
| HOGA1 | down | 0.015188 | 0.003765 | 0.41462 | -1.27014 |
| ZNF487 | down | 0.001373 | 0.00024 | 0.405271 | -1.30304 |
| PICART1 | down | 0.045444 | 0.014 | 0.480583 | -1.05714 |
| NRAV | down | 0.026541 | 0.007307 | 0.450794 | -1.14946 |
| PRECSIT | down | 1.75E-06 | 1.53E-07 | 0.422069 | -1.24445 |
| LINC01963 | down | 0.043699 | 0.013354 | 0.462406 | -1.11277 |
| TFIP11-DT | down | 0.042617 | 0.012951 | 0.265174 | -1.91499 |
| LINC01977 | down | 0.000304 | 4.39E-05 | 0.316573 | -1.65939 |
| MAFG-DT | down | 0.022086 | 0.005874 | 0.432359 | -1.2097 |
| HERPUD2-AS1 | down | 0.000318 | 4.61E-05 | 0.484106 | -1.04661 |
| HK2-DT | down | 0.022596 | 0.006036 | 0.40031 | -1.32081 |
| LENG9 | down | 0.000149 | 2.01E-05 | 0.498067 | -1.00559 |
| RN7SL1 | down | 0.006018 | 0.001285 | 0.028868 | -5.1144 |
| RAB7B | down | 0.000194 | 2.68E-05 | 0.45593 | -1.13311 |
