# Supplementary material for: Restoring expression of tumour suppressor PTEN by engineered circular RNA‐enhanced Osimertinib sensitivity in non‐small cell lung cancer
Source: Clin Transl Med. 2024 Aug 21;14(8):e1792. doi: 10.1002/ctm2.1792 (PMC11337465; doi:10.1002/ctm2.1792)
Supplement: Supplementary file 5 — Supporting Information [file CTM2-14-e1792-s002.docx]

Material and Methods

Cells Lines

293T (Institute of Biochemistry and Cell Biology of Chinese Academy), DLD1 and DLD1 PTEN^-/-^ (School of Basic Medical Sciences Peking University)were cultured in Dulbecco’s Modified Eagle’s Medium (4.5g/L glucose) supplemented with 10% fetal bovine serum (FBS, Gibco) and 1% penicillin/streptomycin (P/S). H1299, PC9 and HCC827 (Institute of Biochemistry and Cell Biology of Chinese Academy) were cultured by RPMI 1640 with 10% FBS and 1% P/S. HCC827OR and PC9OR were gifted by Nanjing Medical University and cultured by RPMI 1640 with 10% FBS and 1% P/S. All cells were maintained at 37 °C and 5% CO2.

In vitro RNA synthesis

The designed RNA sequences were cloned into pU57 vector to construct the plasmids and the plasmids were synthesized by Nanjing GenScript Biotech Corporation. The plasmids were extracted by TIANpure Mini Plasmid Kit (TIANGEN, DP104). Then, plasmids were linearized by endonuclease. Next, high-fidelity Taq enzyme kit was used to amplify the designed DNA segments to construct DNA template. RNA was synthesized by in vitro transcription using HiScribe® T7 High Yield RNA Synthesis Kit (NEB, E2040S) from DNA template following the manufacturer’s instructions and incubation at 37℃ for 3 hours. Of note, the complete replacement of uridine with N1-methylpseudouridine (Jiangsu Synthgene Biotechnology Co., Ltd, China) for modified linear RNA. After that, reactions were treated with DNase I (NEB, M0303S) for 10 min. For circRNA, additional GTP was added to a final concentration of 2 mM and reactions were heated at 55 °C for 20 min. then, RNA was column purified by Monarch® RNA Cleanup Kit (NEB, T2040L). For linear RNA, the reactions after DNase I treatment were purified via a Monarch® RNA Cleanup Kit. RNA was capped using mRNA cap-2′-O-methyltransferase (NEB, M0366S) and Vaccinia capping enzyme (NEB, M2080S) in according to the manufacturer’s instructions of One-Step Capping and 2´-O-Methylation. Poly-adenosine tails were added to capped linear RNAs using E. coli PolyA Polymerase (NEB, M0276S) according to manufacturer’s instructions, and completed mRNA was column purified.

RNA transfection in vitro

Cells were seeded in 6-well or 12-well plates overnight. Then, the synthetic RNA was transfected into cells using Lipofectamine^TM^ 2000 (Thermo Fisher Scientific, MA, USA) according to the manufactures’ protocol. After six hours, the cells were covered by fresh medium.

Reverse transcription and real time quantitative polymerase chain reaction (qPCR)

Total RNA was extracted from tumor tissues and cells using TRIzol Reagent (Invitrogen, Carlsbad, CA, USA). The procedure of reverse transcription of RNA was followed the instruction of PrimeScript™ RT Master Mix (Perfect Real Time) (Takara Biomedical Technology, Japan). Each quantitative polymerase chain reaction was performed in Biorad Biosystem with triplicate samples in total reaction volume of 10 ul (Thermo Fisher Scientific, MA, USA). The gene GAPDH or β-actin was used as the internal parameter. All experiments were performed in triplicate. The primers included in this study were showed in Table S1.

Western blot

The protein of tissues or cells was extracted with Western and IP lysis buffer (Beyotime, Shanghai, China) with 1%PMSF on ice for 30 min and centrifuged for 20 min at 12,000 g at 4°C. The supernatants were transferred into other fresh 1.5 ml tube and the protein concentration was measured by bicinchoninic (BCA) kit (LABLEAD, Bejing, China). Protein samples were separated by dodecyl sulfate-polyacrylamide gel electrophoresis for 70 min and transferred to PVDF membrane. After being blocked with 5% non-fat milk or BSA in Tris-buffered saline with Tween-20 (TBST) (Solarbio, Beijing, China), the membranes were incubated at 4°C overnight with primary antibody. Next, the membranes were washed three times with TBST and incubated with HRP-labeled mouse- or rabbit- secondary antibody for 2 hours at room temperature. Finally, the membranes were detected with chemiluminescent HRP substrate (NCM Biotech, Shanghai, China) in an electrogenerated chemiluminescence imaging system (Clinx Science Instruments, Shanghai, China). The primary antibodies included in this study were in Table S1.

Immunohistochemistry (IHC)

After dewaxing at 60°C about 5 hours and rehydration, the paraffin sections were put into EDTA retrievals at 100°C for 7 minutes and allowed to cool to room temperature. Next, the sections were blocked by 3% H2O2 for 25 minutes. Then, 1X Animal-free blocking solution (CST) was used to block the sections for 10 min and incubated with primary antibody overnight in 4°C. The slides were incubated with HRP-labeled secondary antibody (Beyotime, Shanghai, China) for 1 h at room temperature. Then, the slides were stained with diaminobenzidine (Solarbio, Beijing, China) and observed under a microscope. Finally, the sections were stained cell nucleus with hematoxylin and sealed with neutral resin

Cell apoptosis

Cell apoptosis was detected by Flow Cytometry (Annexin V-FITC Apoptosis Detection Kit, Beyotime, Shanghai, China) or Fluorescein (FITC) TUNEL Cell Apoptosis Detection Kit (Servicebio, Wuhan, China). All assays performed according to the manufactures’ instruction.

Cell proliferation

CCK8, EdU and colony formation assay were used to evaluated the proliferation ability of cells. Cells were plated in 96-well plates at a density of 1 × 10^3^ cells/well and cell viability was investigated 72 h. 10 μl of CCK-8 solutions (LABLEAD, Bejing, China) was added to each well and then put the 96-well plate into cell incubator. After two hours, the absorbance at 450 nm was recorded. EdU Cell Proliferation Kit (Beyotime, Shanghai, China) was performed according to the manufacture’s instruction. For colony formation assays, a total of 500 cells was seeded into each well of the 6-well plate and cultured for 10-12 days. Medium was replaced each 2 days. Cells were washed with PBS, fixed with 4% paraformaldehyde, and stained with crystal violet (Beyotime, Shanghai, China). After staining, photographs were taken, and numbers of colonies were counted.

Agarose gel electrophoresis

The RNA, cDNA and PCR products were analyzed by 2% agarose gel electrophoresis with 1×TAE running buffer. RNA or DNA was separated by electrophoresis at 120 V for 30-35 min. The bands were visualized by UV irradiation.

High-Performance Liquid Chromatography (HPLC) purification

HPLC fractionation was performed with a 4.6×300mm size exclusion column (Sepax Technologies, 215980P-4630) with particle size of 5um and pore size of 2000 Å. Nuclease-free PBS buffer (pH=6) was used as the mobile phase at a flow rate of 0.5 mL/minute. RNA was detected by UV absorbance at 260 nm, but was collected without UV detection. RNA fractions were manually collected and re-precipitation with Ammonium acetate (5M).

Ammonium acetate precipitation of RNA

Add 0.15 volumes of 5 M ammonium acetate and mix. After add 2.5 volumes of 100% ethanol, incubate overnight at -80℃. Then, pellet RNA at 12, 000g for 15 min. Carefully remove the supernatant and wash the RNA pellet (do not try to resuspend pellet) by adding 2.5 volumes of 70% ethanol and allowing the pellet to soak for 2 min. Pellet at 12, 000g for 5 min and remove all the ethanol. Allow the pellet to dry in open tube covered with a Kimwipe for 30min at room temperature and dissolve RNA in nuclease-free H_2_O.

Encapsulation of RNA by Lipid Nanoparticle (LNP)

The mRNA or circRNA was diluted in a sodium acetate buffer with a pH of 4 at a concentration of 50 mM. The lipids were dissolved and diluted in ethanol at molar ratios of 50:10:38.5:1.5 for ionizable lipid (SM102), DSPC, cholesterol, and PEG lipid, respectively. The mRNA or circRNA and lipid solutions were mixed using a Microfluidic LNP preparation instrument (NESTAR (SHANGHAI) NANO TECHNOLOGY CO., LTD) at a flow rate of 4.5 mL/min for the aqueous phase and 1.5 mL/min for the organic phase, with a volume ratio of 3:1. Next, the resulting mixture was diluted with PBS and the LNPs were concentrated in Amicon Ultra Centrifugal Filters (Merck Millipore, 50 kDa molecular weight cutoff). Size was measured with a ZetaVIEW (Particle Metrix, Germany). The mRNA or circRNA encapsulation efficiency was determined using the Quant-iT Ribogreen RNA assay from Thermo Fisher. It is performed by comparing the amount of RNA detected in LNPs that were completely lysed in the presence of 1% Triton X-100 (which allows the detection of all RNA, including encapsulated RNA) with the amount of RNA detected in LNPs in TE buffer (where only non-encapsulated RNA is detectable).

Tumor xenograft

The male BALB/c-nu mice (4 to 5-week-old) were purchased from Beijing Charles River Laboratory Animal Technology. All mice were housed under the SPF-grade conditions in the animal facility of Peking University Animal Laboratory and all animal experiments strictly adhered to the compliance standards of Institutional Animal Care and Use Committee. For validation part, mice were subcutaneously injected with 5×10^6^ PC9OR cells together with Matrigel (Corning). The mix ratio of cells in PBS and Matrigel was 1:1 by volume. Osimertinib (MCE, HY-15772) was is administered by Gavage and the dose is 10mg/kg. RNA-LNP (10μg/mouse) were injected intra-tumoral into mouse groups every there days for therapeutic usage. Tumor volume was measured every two days since day 7 by Vernier Calipers. The tumor volume was calculated by the formation: V = length×width×width/2 (mm3). For contrast part, mice were subcutaneously injected with 5×10^6^ PC9OR cells together with Matrigel (Corning). The dose of Osimertinib is 15mg/kg. RNA-LNP (10μg/mouse) were injected intra-tumoral into mouse groups every four days for therapeutic usage. All body weight of mice was recorded every two days. Mice were sacrificed when the tumor volume reached 1,700 mm^3^.

RNA sequencing (rRNA depletion)

(1) sample preparation. Total RNA was extracted from cell or animal tissue by Trizol reagent (Invitrogen) separately. The RNA quality was checked by Bioanalyzer 2200 (Agilent) and kept at -80℃. The RNA with RIN >6.0 is right for rRNA depletion. (2) cDNA Library construction. The cDNA libraries were constructed for each pooled RNA sample using the NEBNext® Ultra™ Directional RNA Library Prep Kit for Illumina according to the manufacturer’s instructions. Briefly, the protocol consists of the following steps: depletion of rRNA and fragmented into 150-200 bp using divalent cations at 94 ℃ for 8 min. The cleaved RNA fragments were reverse-transcribed into first-strand cDNA, second-strand cDNA synthesis, fragments were end repaired, A-tailed and ligated with indexed adapters. Target bands were harvested through AMPure XP Beads(Beckman coulter) .The products were purified and enriched by PCR to create the final cDNA libraries and quantified by Agilent2200. The tagged cDNA libraries were pooled in equal ratio and used for 150 bp paired-end sequencing in a single lane of the Illumina HiSeqXTen. (3) RNA sequencing Mapping. Mapping of pair-end reads. Before read mapping, clean reads were obtained from the raw reads by removing the adaptor sequences, reads with >5% ambiguous bases (noted as N) and low-quality reads containing more than 20% of bases with qualities of <20. The clean reads were then aligned to human genome (version: GRCh38 NCBI) using the hisat2. Differential gene and transcript expression analysis of RNA-seq experiments with TopHat and Cufflinks^1^. HTseqwas used to count gene and lncRNA counts and RPKM method was used to determine the gene expression^2^. (4) Differentially expressed Gene-Finder: We applied EBSeq algorithm to filter the differentially expressed genes, after the significant analysis, p value and FDR analysis under the following criteria^3^. Gene ontology (GO) analysis was performed to facilitate elucidating the biological implications of unique genes in the significant or representative profiles of the target gene of the differentially expressed miRNA in the experiment^4^. We downloaded the GO annotations from NCBI (http://www.ncbi.nlm.nih.gov/), Uni-Prot (http://www.uniprot.org/) and the Gene Ontology (http://www.geneontology.org/). Fisher’s exact test was applied to identify the significant GO categories and FDR was used to correct the p-values. Pathway analysis was used to find out the significant pathway of the differential genes according to KEGG database. We turn to the Fisher’s exact test to select the significant pathway, and the threshold of significance was defined by p-value and FDR^5^.

Co-immunoprecipitation (IP) assay

Co-immunoprecipitation assay was performed with Immunoprecipitation Kit (Proteintech, China). Briefly, the protein was extracted from HCC827OR cells with IP lysis buffer supplemented with protease inhibitor. The protein concentration was quantified using the BCA Protein Assay Kit (LABLEAD, China). Then the cellular lysis was incubated with rabbit anti-PTEN antibody (Proteintech, China), anti-AKR1C2 antibody (Abcam, USA) or rabbit IgG (Proteintech, China) at 4 °C overnight. PTEN, AKR1C2 and its interacting proteins were immunoprecipitated with the antibody-conjugated beads at 4℃ for 3 h. The protein-bead complexes were washed five times with washing buffer, and then eluted twice with 40 μl elution buffer. Elution fractions were then analyzed using Western blot.

Intracellular ROS detection

DCFH-DA (Solarbio, China)was used as a fluorescent indicator to monitor the levels of intracellular ROS in living HCC827OR and PC9OR cells. 1.5 × 10^5^ HCC827OR or PC9OR cells were initially seeded in 6-well plates per well and cultured for 24 h. cPTEN_NeoAna was transfected into cells in the next day. At third day, the cells were then incubated with fresh media or Osimertinib. The cells were stained with DCFH-DA (10 µM) for fluorescence imaging after 24h incubation.

Statistical analysis

Statistical analysis of the results was performed by a two-tailed unpaired Welch’s t-test, assuming unequal variances. Differences were considered significant when p<0.05. For all studies, data presented is representative of one independent experiment.

Reference

1. Trapnell C, Roberts A, Goff L, et al. Differential gene and transcript expression analysis of RNA-seq experiments with TopHat and Cufflinks. *Nature protocols*. Mar 1 2012;7(3):562-78. doi:10.1038/nprot.2012.016

2. Anders S, Pyl PT, Huber W. HTSeq--a Python framework to work with high-throughput sequencing data. *Bioinformatics (Oxford, England)*. Jan 15 2015;31(2):166-9. doi:10.1093/bioinformatics/btu638

3. Benjamini Y, Drai D, Elmer G, Kafkafi N, Golani I. Controlling the false discovery rate in behavior genetics research. *Behavioural brain research*. Nov 1 2001;125(1-2):279-84. doi:10.1016/s0166-4328(01)00297-2

4. Ashburner M, Ball CA, Blake JA, et al. Gene ontology: tool for the unification of biology. The Gene Ontology Consortium. *Nature genetics*. May 2000;25(1):25-9. doi:10.1038/75556

5. Draghici S, Khatri P, Tarca AL, et al. A systems biology approach for pathway level analysis. *Genome research*. Oct 2007;17(10):1537-45. doi:10.1101/gr.6202607
